# Predicting response to multidrug regimens in cancer patients using cell line experiments and regularised regression models

**DOI:** 10.1186/s12885-015-1237-6

**Published:** 2015-04-08

**Authors:** Steffen Falgreen, Karen Dybkær, Ken H Young, Zijun Y Xu-Monette, Tarec C El-Galaly, Maria Bach Laursen, Julie S Bødker, Malene K Kjeldsen, Alexander Schmitz, Mette Nyegaard, Hans Erik Johnsen, Martin Bøgsted

**Affiliations:** 1 Department of Haematology, Research Section, Aalborg University Hospital, Sdr. Skovvej 15, 9000 Aalborg, Denmark; 2 Department of Hematopathology, The University of Texas MD Anderson Cancer Center, Houston, TX USA; 3 Department of Biomedicine, Aarhus University, Aarhus, Denmark; 4 Department of Clinical Medicine, Aalborg University, Aalborg, Denmark; 5 Clinical Cancer Research Center, Aalborg University Hospital, Aalborg, Denmark

**Keywords:** Drug screen, Drug resistance, Preclinical model, Gene expression profiling, Cancer

## Abstract

**Background:**

Patients suffering from cancer are often treated with a range of chemotherapeutic agents, but the treatment efficacy varies greatly between patients. Based on recent popularisation of regularised regression models the goal of this study was to establish workflows for pharmacogenomic predictors of response to standard multidrug regimens using baseline gene expression data and origin specific cell lines. The proposed workflows are tested on diffuse large B-cell lymphoma treated with R-CHOP first-line therapy.

**Methods:**

First, B-cell cancer cell lines were tested successively for resistance towards the chemotherapeutic components of R-CHOP: cyclophosphamide (C), doxorubicin (H), and vincristine (O). Second, baseline gene expression data were obtained for each cell line before treatment. Third, regularised multivariate regression models with cross-validated tuning parameters were used to generate classifier and predictor based resistance gene signatures (REGS) for the combination and individual chemotherapeutic drugs C, H, and O. Fourth, each developed REGS was used to assign resistance levels to individual patients in three clinical cohorts.

**Results:**

Both classifier and predictor based REGS, for the combination CHO, were of prognostic value. For patients classified as resistant towards CHO the risk of progression was 2.33 (95% CI: 1.6, 3.3) times greater than for those classified as sensitive. Similarly, an increase in the predicted CHO resistance index of 10 was related to a 22% (9%, 36%) increased risk of progression. Furthermore, the REGS classifier performed significantly better than the REGS predictor.

**Conclusions:**

The regularised multivariate regression models provide a flexible workflow for drug resistance studies with promising potential. However, the gene expressions defining the REGSs should be functionally validated and correlated to known biomarkers to improve understanding of molecular mechanisms of drug resistance.

**Electronic supplementary material:**

The online version of this article (doi:10.1186/s12885-015-1237-6) contains supplementary material, which is available to authorized users.

## Background

Patients suffering from cancer are usually treated with a range of chemotherapeutic agents, but the treatment efficacy varies greatly between patients. As new therapeutic possibilities emerge, it is becoming increasingly important to identify individual patients who are unlikely to respond satisfactorily and who may benefit from carefully selected agents [1].

Resistance gene signatures (REGSs) for prediction of chemoresistance have been investigated extensively since the development of microarrays. The REGS can be grouped into classifiers and predictors where the classifiers assign a probability for each patient as sensitive or resistant, and the predictors assign each patient a numeric value where higher values indicate greater drug resistance. Studies generating REGS can either be performed by analysis of clinical data generated *in vivo* followed by a prognosis based reverse-translational approach, or by analysis of laboratory data generated *in vitro* followed by a predictive drug screen approach.

Cell line based studies on drug resistance have typically been founded on categorisation of the cell lines into sensitive, resistant, and intermediate groups based on summary statistics for dose response experiments. Subsequently, differentially expressed genes between the sensitive and resistant cell lines are determined and used to generate a REGS classifier typically based on a version of Linear Discriminant Analysis (LDA). Publicly available data from the NCI60 cell line panel generated by the National Cancer Institute (NCI) have been used extensively in such studies [2-7]. However, the approach have been plagued with issues of irreproducibility [8-10] and the results have been ambiguous [3,4]. Several authors have argued that a cancer specific cell line panel could improve performance [4,11-13]. With varying success such an approach was used by Liedtke et al. [12] and Boegsted et al. [4] for breast cancer, and multiple myeloma, respectively. In both articles a variant of LDA was used to establish a REGS classifier neither of which resulted in predictions related to clinical outcome.

The working hypothesis is that the combined expression pattern of a group of genes within a malignant cell determines that cell’s level of resistance towards a specific drug. The aforementioned REGSs have been founded on genes selected by their marginal association with drug resistance. Multivariate regression techniques regularised by a penalty such as elastic net [14] may be utilised to establish REGSs based on genes selected due to their simultaneous capability of predicting drug resistance. In additition to the REGS classifier based on LDA, Boegsted et al. [4] used such an approach to establish a REGS predictor based on multivariate regression for which predictions were associated with treatment outcome. Similarly, by use of the cancer genome project [15] (CGP) and Cancer Cell Line Encyclopedia [16] (CCLE) Papillon-Cavanagh et al. [17] showed that REGS predictors established using multivariate regression techniques seemed to perform better than those based on marginal associations. Recently, Geeleher et al. [18] validated that such an approach could generate REGSs of prognostic value for patients treated with a single chemotherapeutic agent.

The concept of the present work is that multivariate regression techniques enable development of combined REGS for patients treated with a range of drugs. For instance patients with newly diagnosed diffuse large B-cell lymphoma (DLBCL) are usually treated with a multi-agent chemotherapy regimen containing rituximab (R), cyclophosphamide (C), doxorubicin (H), vincristine (O), and prednisolone (P). Hence, in order to predict treatment outcome of such patients it is necessary to combine the developed REGS. However, only a relatively small number of drugs have been tested in either CGP or CCLE and of the three chemotherapeutic agents of R-CHOP (C, H, and O) only H has been tested so far. Thus, in order to develop REGSs for the standard treatment of DLBCL, and many other cancers, it is necessary to develop an in laboratory drug screen of the used chemotherapeutics. Since it is not feasible for small laboratories to perform such experiments in a large-scale a smaller cell line screen of origin specific cell lines is used.

In Falgreen et al. [19] we recently published a method for analysing dose response experiments that accounts for well-known issues such as varying cell line growth kinetics and variation in seeding concentration. By combining this approach with a panel of human B-cell cancer cell lines (HBCCL), the specific aims of this study were to 1) ensure that REGSs developed using carefully selected cell lines analysed to the requirements of Falgreen et al. [19] can be of similar, or even superior, prognostic value as those developed using a large-scale study, 2) generate REGS classifiers and predictors for resistance towards the potent chemotherapeutic agents in R-CHOP, 3) combine them into REGSs for CHO, and 4) compare the performance of REGS classifiers and predictors in clinical data. To support the concept, resistance signatures were tested in three clinical datasets from DLBCL patients treated with R-CHOP therapy [20,21].

## Methods

**Figure 1:**
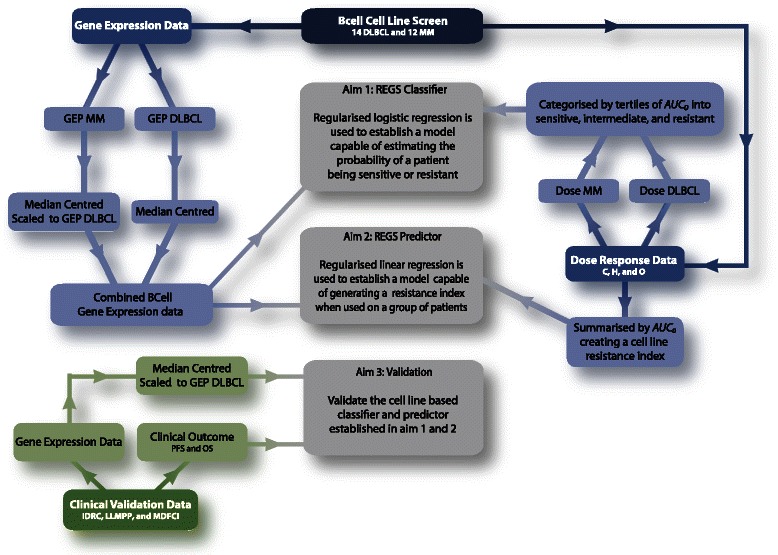
**Flow diagram of the analysis strategy.** The blue and green boxes indicate *in vitro* and *in vivo* data, respectively. The grey boxes indicate the aims of the statistical analysis. First, test the level of resistance towards the three drugs C, H, and O successively on B-cell cancer cell lines by dose response experiments in accordance with [19]. Secondly, obtain baseline gene expression data for each cell line before treatment. Thirdly, establish a REGS classifier capable of estimating the probability of a tumour sample being sensitive or resistant. This was done by grouping the third most sensitive and third most resistant cell lines for each drug and establishing a REGS classifier by regularised logistic regression. Fourth, establish a REGS predictor based on the sensitivity level of each cell line without grouping them into sensitive and resistant. This was done by using the estimated drug specific resistance for each cell line and establishing a REGS predictor by regularised linear regression. Such a REGS predictor is unable to estimate the probability of a tumour sample being sensitive or resistant; however, the statistical analysis may gain power by using all cell lines without categorising them. Fifth, combine the developed REGSs into a classifier and predictor for CHO. Finally, sixth, validate the established REGSs in independent clinical cohorts.

The focus of this study was to develop REGSs for the combination treatment CHO constituting the main part of R-CHOP first line treatment of DLBCL. Thus, the task was not to explain the biological mechanisms leading to chemo-resistance but to establish REGS capable of predicting whether a patient is sensitive or resistant to chemotherapeutic agents [22]. Hence the predictive capabilities of the established REGSs were evaluated in pre-treatment tumour samples. Such a strategy involves intensive data generation in the laboratory and is succeeded by data management and advanced statistical analysis [8]. The analysis workflow is outlined in Figure 1 and each step is described in detail in the following sections. All analyses were performed with R version 3.1.0 [23] and several add-on packages. Detailed session information and full documentation of the statistical analysis is provided by a Knitr document, see Additional file 1: Text S1. Knitr enables the integration of R code into LaTeX providing reproducible data analysis.

### Data acquisition from the CGP screen

Gene expression data on the Affymetrix Genechip HG-U133A array was obtained from ArrayExpress under accession number E-MTAB-783. The CGP dose response data for H contained in the file *gdsc_manova_input_w2.csv* were downloaded from the CGP website: www.cancerrxgene.org. Haibe-Kains et al. [24] found the area under the dose response curve (*AUC*) to be the most consistent summary statistic between the two cell line screens CGP and CCLE, hence all analyses were based on this.

### The HBCCL screen and culture conditions

The HBCCL panel consisted of 11 multiple myeloma (MM), one plasmacytoma, one undifferentiated lymphoma, and 13 DLBCL cell lines. Detailed information on each cell line is available in Additional file 2: Table S1. The plasmacytoma and undifferentiated lymphoma cell lines were treated as an MM and a DLBCL cell line, respectively. The cell lines were cultured under standard conditions at 37°C in a humidified atmosphere of 95% air and 5% CO_2_ with the appropriate medium (RPMI1640 or IMDM), fetal bovine serum (FBS), and 1% penicillin/streptomycin. Penicillin/streptomycin 1%, RPMI1640, IMDM and FBS were purchased from Invitrogen. The origin of the cell lines is as listed: KMM-1 and KMS-11 were obtained from JCRB (Japanese Collection of Research Bioresources). AMO-1, DB, HT, KMS-12-PE, KMS-12-BM, LP-1, MC-116, MOLP-8, NCI-H929, NU-DHL-1, NU-DUL-1, OPM-2, RPMI-8226, SU-DHL-4, SU-DHL-5, and U-266 were purchased from DSMZ (German Collection of Microorganisms and Cell Cultures). FARAGE, HBL-1, OCI-Ly3, OCI-Ly7, OCI-Ly19, RIVA, SU-DHL-8, and U2932 were kindly provided by Dr. Jose A. Martinez-Climent (Molecular Oncology Laboratory University of Navarra, Pamplona, Spain). Finally, Dr. Steven T. Rosen generously provided MM1S [25].

The identity of the cell lines was verified by DNA barcoding performed every time a cell line was thawed and brought into culture. As DNA was not available from each passage, a PCR analysis was performed using 0.2 ng RNA thereby amplifying traces of genomic DNA using the sensitive AmpFISTR Identifiler PCR amplification kit (Applied Biosystems, CA, USA). The amplified products were analysed by capillary electrophoresis (Eurofins Medigenomix GmbH, Applied Genetics, Germany). The resulting FSA file was analysed using the Osiris software (ncbi.nlm.nih.gov/projects/SNP/osiris) and the identity of the cell lines were established by comparing to DNA barcoding profiles for known cell lines obtained with the same markers and made publicly available at DSMZ.

### CHO dose response experiments

The effect of the drugs C, H, and O on viable proliferating cells was measured on 24, 26, and 24 different human B-cell cancer cell lines in concordance with [19]. Because C requires hepatic activation to produce its active metabolite 4-hydroxy-cyclophosphamide, the synthetic oxazaphosphorine derivative mafosfamide with antineoplastic properties was used as surrogate. The number of viable cells in the culture was estimated by absorbance measurements (CellTiter 96 Aqueous One Solution Reagent, Promega, USA) as described by the manufacturer. A linear relationship between the cell count and absorbance measurements was obtained by seeding 15,000-60,000 cells in 120 μl media per well in a 96 well plate for 24 h at standard tissue culture conditions. Subsequently, 18 increasing concentrations of C, H, or O were added to each cell line in triplicates. The absorbance was measured immediately and after 48 hours of drug exposure using the CellTiter reagent (CellTiter 96 Aqueous One Solution Reagent, Promega, USA) and quantified at 492 nm using the Fluostar Optima (BMG LABTECH, Germany). All wells were seeded with cells but border effects were circumvented by only including non-border wells for analysis.

The highest drug concentrations to which the cells were exposed were 80, 10, and 20 μg/ml for C, H, and O, respectively. Twofold dilution series were used. C was purchased as powder from Niomech (Germany) and dissolved in isotonic salt-water aliquots and stored at −80°C for a few weeks. H and O were purchased from PharmaCoDane (Denmark) and TEVA (USA), respectively. The drugs were diluted in isotonic salt-water prior to use.

### RNA microarray analysis

All gene expression profiles (GEP) were performed using the Affymetrix microarray platform and standard procedures. Total RNA was extracted from the 26 drug naïve cell lines using TRIzol Reagent (Invitrogen, UK) and the RNeasy Mini kit (Qiagen, Germany). The quality was checked by Agilent 2100 Bioanalyzer (Agilent, USA) (all RIN values above 9). The samples were labelled using the GeneChip Expression 3′ amplification One-cycle Target Labeling (Affymetrix) (input 5 μg total RNA) and hybridised to the Affymetrix GeneChip HG-U133 Plus 2.0 array according to the manufacturer’s protocol. The .CEL-files were generated by Affymetrix Gene-Chip Command Console Software (AGCC) and deposited at the NCBI Gene Expression Omnibus (GEO) repository. The data fulfils the requirements of being MIAME compliant and the .CEL files for the 26 cell line microarrays are available at http://www.ncbi.nlm.nih.gov/geo/ under GEO accession number GSE53798.

### Clinical cohorts

The workflow for generating REGS is exemplified for the haematological malignancy DLBCL. Patients with newly diagnosed DLBCL are usually treated with a multi-agent chemotherapy regimen containing C, H, O, and P. This so-called CHOP regimen was developed decades ago and has ever since been the backbone of DLBCL treatment. The only significant improvement during the last decade has been the addition of the monoclonal CD20 antibody rituximab (R) to CHOP (R-CHOP), which has led to an increase in overall survival (OS) of 10-15% [26-30]. However, with a 3-year progression free survival (PFS) of 55-87% depending on the number of risk factors there is still room for improvement [31]. An important clinical challenge is how to manage the large number of patients with disease primary refractory to R-CHOP. Currently used treatment algorithms for DLBCL are still based on the International Prognostic Index (IPI) which is derived from simple and easily available clinicopathological features [32,33]. Within the individual risk groups, however, there is great variation in outcome which indicates that additional features such as tumour-biological heterogeneity impact on drug sensitivity [20,34,35]. Three patient cohorts, with GEP datasets available at http://www.ncbi.nlm.nih.gov/geo/, consisting of DLBCL patients treated with R-CHOP were used to validate the generated REGS:

1. The International DLBCL Rituximab-CHOP Consortium MD Anderson (IDRC) cohort of 470 DLBCL patients treated with R-CHOP first-line therapy [36]. Gene expression data are available at GEO under accession number GSE31312. The data collection was approved by the Institutional Review Board at The University of Texas MD Anderson Cancer Center in Houston, Texas [36].
2. The Lymphoma/Leukemia Molecular Profiling Project R-CHOP (LLMPP) cohort of 233 DLBCL patients treated with R-CHOP first-line therapy [20]. GEP data from the tumour before treatment and clinical information is publicly available under GEO accession number GSE10846. Progression free survival on the patients was made available by personal communication with Lenz. The data was studied in accordance with a protocol approved by the Institutional Review Board of the National Cancer Institute [20].
3. The Mayo-Dana-Farber Cancer Institute (MDFCI) cohort of 67 DLBCL patients treated with R-CHOP first-line therapy [21]. GEP data from tumour before treatment and clinical information is available under GEO accession number GSE34171. The data was studied in accordance with protocols approved by the Institutional Review Board from three institutions (Mayo Clinic, Brigham & Women Hospital, and Dana-Farber Cancer Institute) [21].

To determine whether or not the established REGS predict prognosis in DLBCL treated with R-CHOP, a cohort consisting of patients not treated with the combination therapy R-CHOP was used. Here the University of Arkansas for Medical Sciences (UAMS) cohort of 565 multiple myeloma patients was used [37]. The institutional review board of UAMS approved data collection and research [37]. The patients from UAMS received total therapy 2 and 3 (TT2 and TT3). Although these regimens both included doses of C, H, and O in various combinations and in addition to several other drugs (thalidomide, bortezomib, cisplatin, etoposide) the most important disease controlling elements of the TT2 and TT3 regimens were melphalan-based tandem transplants. Thus, for the multiple myeloma patients from UAMS C, H, and O did not form the chemotherapeutic backbone of a curative treatment as for the DLBCL patients. GEP data from plasma cells and clinical information is available under GEO accession number GSE24080. The UAMS.

All GEP data are on the Affymetrix Genechip HG-U133 Plus 2.0 array and all DLBCL cohorts included information on IPI. All research has been performed in compliance with the Helsinki declaration.

### Statistical analysis

#### CHO dose response analysis

Dose response experiments are conventionally summarised by dose response curves where the net growth of a cell line treated with a range of concentrations are compared to the net growth of the same cell line untreated. However, this may lead to dose response curves that are biased so fast growing cell lines appear overly sensitive [19]. Here, we used an alternative method for summarising dose response experiments, which has been described in [19]. This approach generates dose response curves by comparing the growth rates of a treated cell line with the growth rate of the same untreated cell line thereby removing the aforementioned bias under the assumption of exponential growth [19].

According to [38] the area under the dose response curve *AUC* is the overall best performing summary statistic of a dose response experiment. In concordance with this, the area under the dose response curve where this is positive (*AUC*_*0*_*)* was used to summarise the dose response experiments [19].

### Microarray pre-processing

The .CEL-files associated with the HBCCL, CGP, and the clinical cohorts were RMA pre-processed using the Bioconductor package affy [39,40]. The pre-processed GEP data for CGP along with the GEP data for the clinical cohorts were probe-set wise centred to have median equal to zero.

The pre-processed GEP data for HBCCL was split into two datasets consisting of the DLBCL and MM cell lines. The DLBCL GEP dataset was then probe-set wise centred to have median equal to zero. The probe-sets of the MM GEP data were centred to have median equal to zero and scaled to have the same variance as that observed in the DLBCL GEP data. The GEP data of the DLBCL and MM datasets were then merged together resulting in the HBCCL dataset.

In the development of REGS based on both the HBCCL and CGP, each gene interrogated by multiple probe-sets was represented by the most variable within the concerned dataset. In order to homogenize the clinical and cell line based GEP data each gene of the clinical data was scaled to have the same variance as that observed in either CGP or the DLBCL GEP data of HBCCL.

### Establishment of *in vitro* based REGS

For each of the three drugs multivariate regression models were used to establish REGSs that estimate a malignant tumour’s resistance towards the drug. Here the result of the dose response experiments was used as the outcome variable and the GEPs as explanatory variables. However, the vast number of probe-sets present on the microarray greatly outnumbers the cell lines. Additionally, there is collinearity among the genes, and the set of active genes that control the underlying biological process is believed to be small. Regression under these ill-posed circumstances is typically handled by a regularisation parameter, which shrinks the regression coefficients by penalising their size. Increasing the amount of regularisation increases the shrinkage of each coefficient. Here we used the elastic net penalty [14,41] which combines the lasso [42] and ridge regression [43]. Regression with elastic net ensures sparse solutions by forcing small coefficients to be zero and thereby estimates the set of active genes whilst fitting the model. Similar to the lasso this penalty ensures simultaneous variable selection and model estimation. In contrast to the lasso, the elastic net penalty is capable of selecting more variables than samples.

The aforementioned collinearity among genes is partially caused by genes operating in molecular pathways wherefore their expressions are often highly correlated [44]. We may think of such genes as a group for which an ideal selection method will include the entire group if one gene among them is selected. Notably, when using elastic net, we select correlated variables in groups ensuring that genes operating in pathways are selected together.

The elastic net penalty contains two parameters α and λ. The parameter α determines the degree to which the elastic net penalty should resemble the lasso or ridge penalty with values of 0 and 1 resulting in ordinary ridge regression or the lasso, respectively. As the model parameter increases from 0 to 1, the resemblance towards lasso increases. The regularisation parameter λ determines the amount of shrinkage of the coefficients with larger values inducing more shrinkage until no variables are contained in the model. By plotting the coefficient associated with each probe-set against a range of λ values so called regularisations curves are obtained. For both the regularised logistic and linear regression the R-package glmnet [41] was used to establish the REGS.

#### Regularised logistic regression for establishment of REGS classifiers

Combining the elastic net penalty with logistic regression solved the first aim of the statistical analysis (Figure 1). This approach established a REGS classifier capable of assigning each tumour sample an estimate of the probability of being resistant to each of the three drugs.

For each drug the cell lines of the HBCCL screen were categorised as sensitive, intermediate, or resistant based on tertiles of their *AUC*_*0*_ values. This was done separately for the two disease groups DLBCL and MM to avoid comparison of disease type instead of drug resistance. Similarly, each cell line of the CGP screen was categorised as sensitive, intermediate, or resistant based on tertiles of their *AUC* values. Because there are so many different cancers in CGP this grouping was not done disease wise.

The cell lines in the intermediate group were discarded, and the cell lines categorised as either sensitive or resistant were used to establish the classifier. The model parameter α and shrinkage parameter λ were chosen through leave-one-out cross validation for HBCCL and 20 fold cross validation for CGP. The α and λ parameters were varied over a broad range of values ranging from 0.1 to 1 for α and on a log scale between −6 and 3 for λ. The optimal configuration of the parameters was chosen to be the set minimising the number of misclassifications. For ties the smallest value of both α and λ were chosen. Once the optimal parameters for each drug were chosen and the logistic models were fitted internally from the cell lines, it was possible to estimate the probability of a patient being resistant to each drug individually. This final step was done using the median centred and scaled GEP data as described in the section Microarray Pre-processing.

By use of Graham’s formula the HBBCL based REGS classifiers for C, H, and O were combined into a single REGS classifier for CHO. Let *P*_*C*_, *P*_*H*_, and *P*_*O*_ denote the probabilities of being resistant towards the three drugs C, H, and O individually. Then under the assumption of drug independence the posterior probability of being resistant towards the combination therapy CHO was estimated as: *P*_*C*_*P*_*H*_*P*_*O*_*/(P*_*C*_*P*_*H*_*P*_*O*_*+(1-P*_*C*_*)(1-P*_*H*_*)(1-P*_*O*_*))*.

#### Regularised linear regression for establishment of REGS predictors based on HBCCL

Combining the elastic net penalty with linear regression solved the second aim of the statistical analysis (Figure 1). For the HBCCL panel, this approach established REGS predictors for C, H, and O capable of estimating the *AUC*_*0*_ value for a tumour sample, which indicates that higher values are associated with greater resistance. For the CGP panel the developed REGS predictor for H was based on the *AUC* values.

To account for the two disease origins within the HBCCL panel an indicator variable was included in the regression which was 0 and 1 for the DLBCL and MM cell lines, respectively. This variable was not assigned any penalty and was therefore included in all models. Because some diseases are only presented by one cell line in the CGP panel such an approach was not used in the establishment of the CGP based REGS predictor for H.

The model parameter α and shrinkage parameter λ were chosen through leave-one-out cross validation for HBCCL and 20 fold cross validation for CGP. The α and λ parameters were varied from 0.1 to 1 for α and on a log scale between −0.17 and 7.63 for λ. The optimal configuration of the parameters was chosen to be the set minimising the mean squared prediction error (MSPE). Once the optimal parameters for each drug were chosen and the linear models were fitted internally from the cell lines, it was possible to predict resistance indices for the clinical cohorts. This was done using the median centred and scaled GEP data as described in section Microarray Pre-processing. The HBCCL based REGS predictors for the individual drugs were combined into a single CHO predictor by the geometric mean.

### Evaluation in clinical data

The generated REGS classifiers and REGS predictors were validated in three clinical cohorts to solve the third aim of the statistical analysis (Figure 1). The classifications and predictions were tested using PFS and overall survival (OS) as surrogate endpoints for drug resistance.

#### Comparison of REGS developed using CGP and HBCCL

The REGS classifiers for H based on the HBCCL and CGP screens were used to assign each patient of IDRC, LLMPP, and MDFCI a probability of being resistant. The patients within each dataset were categorised by tertiles of the range of assigned probabilities and the resulting categorisations were analysed using Cox proportional hazards both univariately and adjusted for IPI.

The REGS predictors for H based on the HBCCL and CGP screens were used to assign a resistance index for each patient. For each clinical cohort the resistance indices were analysed using Cox proportional hazards both univariately and adjusted for IPI. To ensure comparable risk assessments for the two REGS predictors the CGP based index was further scaled to have standard deviation equal to that of the HBCCL based index.

#### Retrospective validation on clinical samples

The REGS classifier for each drug was used to assign each patient of IDRC, LLMPP, and MDFCI a probability of being resistant and the probabilities were combined as described above. The patients within each dataset were categorised according to tertiles of the range of assigned probabilities. The predicted categories’ connection to clinical outcome was investigated using Kaplan-Meier survival curves and Cox proportional hazards models as univariate and adjusted for IPI.

The drug specific resistance indices predicted for each cohort were continuous variables where larger values indicated more resistance toward the drug. Cox proportional hazards models were used to investigate the predictive capabilities of the resistance indices in clinical cohorts. For IDRC and LLMPP, PFS were modelled with the resistance index as a linear predictor. Since PFS was not available in MDFCI OS was used instead. The indices were both used in univariate analyses and adjusted for IPI. Since the relationship between clinical outcome and the drug resistance indices may be non-linear, restricted cubic splines were used to model the relationship. These models were likewise adjusted for IPI. The Cox proportional hazard analyses were conducted using the R-packages Hmisc and rms.

The sensitivity and specificity of the REGS classifiers and predictors were investigated using time dependent receiver operating characteristics (ROC) curves for cumulative PFS and OS. The performance of the classifier and predictor based REGS for CHO were compared in terms of area under the ROC curve. The analyses of ROC curves were conducted using the R-package timeROC. This package supports estimation of time dependent ROC curves for censored data and tests for comparing AUCs of competing REGSs measured on the same data [45,46].

The patients of UAMS were categorised as being sensitive, intermediate, or resistant toward the three drugs using the REGS classifiers as described above. The predicted categories were analysed using Kaplan-Meier survival curves and univariate Cox proportional hazards models with OS as endpoint. The patients were also assigned drug specific resistance indices using the REGS predictors. The relationship between predicted resistance indices and OS were modelled by restricted cubic splines and analysed by Cox proportional hazards models.

#### Differential expression between sensitive and resistant patient samples

The REGS classifier for CHO performed significantly better than the corresponding predictor hence differential expression was investigated for the former. Differentially expressed genes between tumours classified by the REGS-CHO classifier as sensitive and resistant DLBCL were detected by the moderated t-test implemented in the Bioconductor package Limma [47]. The number of false discoveries was controlled to be 5%. Furthermore, only genes with a log2 fold change exceeding 1 were considered relevant.

#### GO enrichment

Gene ontology (GO) [48] (www.geneontology.org) enrichment of gene lists was performed by the over representation analysis implemented in the Bioconductor package GOStats [49]. The P-values were adjusted by Holm’s method [50].

For all analyses the significance level was set to 0.05 and the estimated hazard ratios (HR) were given with 95% Confidence Intervals (CI).

## Results

### Developing the HBCCL resistance index

**Figure 2:**
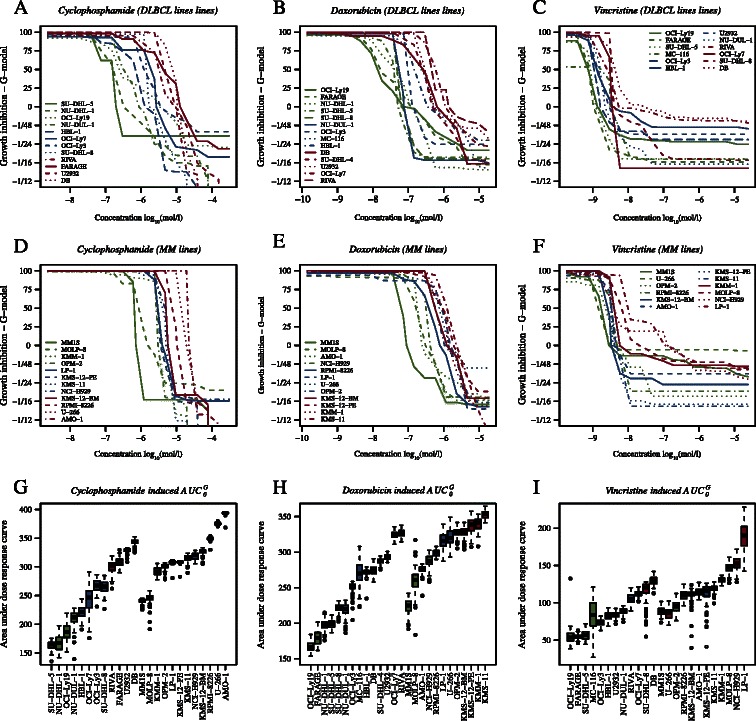
**Dose response curves for the CHO screen.** In panels **A** and **D** dose response curves are shown for the 12 DLBCL and 12 MM cell lines treated with C. In panels **B** and **E** dose response curves are shown for the 14 DLBCL and 12 MM cell lines treated with H. The dose response curves for 12 DLBCL and 12 MM cell lines treated with O are shown in panels **C** and **F**, respectively. Finally, panels **G**, **H**, and **I** show bootstrapped *AUC*_*0*_ values for C, H, and O, respectively. The colours represent the categorisation of the cell lines into tertiles where green, blue, and red denote sensitive, intermediate, and resistant, respectively.

The dose response experiments were analysed in concordance with Falgreen et al. [19] using the area under the positive part of the curve *AUC*_*0*_ as summary statistic. The dose response curves for the three drugs together with boxplots of the bootstrapped *AUC*_*0*_ summary statistics are shown in Figure 2*.* For C the *AUC*_*0*_ values for the DLBCL cell lines ranged from 165 (95% CI: 160, 169) to 346 (339, 348) with SU-DHL-5 and DB as the most sensitive and resistant, respectively. For the MM cell lines the *AUC*_*0*_ values ranged from 242 (CI: 240, 242) to 395 (CI: 391, 394) with MM1S and AMO-1 as the most sensitive and resistant, respectively. For H the *AUC*_*0*_ values for the DLBCL cell lines ranged from 167 (CI: 163, 179) to 327 (CI: 317, 33) with OCI-Ly19 and RIVA as the most sensitive and resistant, respectively. For the MM cell lines the *AUC*_*0*_ values ranged from 227 (CI: 226, 235) to 356 (CI: 342, 358) with MM1S and KMS-11 as the most sensitive and resistant, respectively. For O the *AUC*_*0*_ values for the DLBCL cell lines ranged from 54 (CI: 47, 69) to 131 (CI: 121, 134) with OCI-Ly19 and DB as the most sensitive and resistant, respectively. For the MM cell lines the *AUC*_*0*_ values ranged from 90 (CI: 79, 96) to 187 (CI: 175, 214) with MM1S and LP-1 as the most sensitive and resistant, respectively.

The cell lines were ranked and categorised as sensitive, intermediate, or resistant based on tertiles of the *AUC*_*0*_ values. For C, H, and O the 33% and 66% percentile of the *AUC*_*0*_ were [222, 279], [223, 274], and [71, 96] for DLBCL and [306, 324], [295, 330], and [112, 126] for MM cell lines. For C and O this categorisation gave eight sensitive and resistant cell lines whereas for H nine were categorised as sensitive and resistant (Figure 2G,H, and I).

### Cross validating the elastic net logistic regression models

To avoid over-fitting and limit the number of noise contributing genes the elastic net parameters α and λ were chosen by leave-one-out cross-validation for each of the three drugs. The optimal combination of the parameters and thereby the number of genes were found at the values where the minimum classification error was attained. For the HBCCL screen the results of the cross validation are shown in Additional file 2: Figure S1. For C the minimum 0.31 was attained at α equal to 0.1 and log(λ) equal to −2.27 resulting in a REGS classifier consisting of 73 genes. For H the minimum classification error 0.11 was attained at α equal to 0.1 and log(λ) equal to −6 resulting in a REGS classifier consisting of 118 genes. Finally, the minimum classification error 0.31 was attained at α equal to 0.1 and log(λ) equal to 0.54 for O resulting in a REGS classifier consisting of 32 genes. For the α value resulting in the minimum classification error the regularisation curves are shown in Additional file 2: Figure S2. For the CGP based REGS classifier for H the minimum classification error 0.31 was attained at α equal to 0.45 and log(λ) equal to −2.21 resulting in a REGS classifier consisting of 88 genes. The complete list of genes used in the classifiers is found in Additional file 2: Table S2.

### Cross validating the elastic net linear regression models

Similar to the regularised logistic regression the elastic net parameters α and λ were chosen by leave-one-out cross-validation for each of the three drugs. The optimal combination of the parameters was found at the values where the minimum mean square prediction error (MSPE) was attained. The results of the cross-validation for the HBCCL screen are shown in Additional file 2: Figure S3. For C the minimum 2421 was attained at α equal to 0.3 and log(λ) equal to 1.69 resulting in a REGS predictor consisting of 27 genes. For H the minimum MSPE 2083 was attained at α equal to 0.1 and log(λ) equal to 2.51 resulting in a REGS predictor consisting of 52 genes. Finally, the minimum MSPE 777 was attained at α equal to 0.1 and log(λ) equal to 45 for O resulting in a REGS predictor consisting of 21 genes. For the α value resulting in the minimum classification error the regularisation curves are shown in Additional file 2: Figure S4. For the CGP based REGS classifier for H the minimum classification error 0.03 was attained at α equal to 0.85 and log(λ) equal to −4.23 resulting in a REGS classifier consisting of 141 genes. The complete list of genes used in the predictors is found in Additional file 2: Table S3.

### Comparison of REGSs developed using CGP and HBCCL

The performance of the REGS classifiers and predictors for H based on HBCCL and CGP were compared using the three clinical cohorts IDRC, LLMPP, and MDFCI. The resistance categorisations and indices assigned by the REGS classifiers and predictors, respectively, were analysed using Cox proportional hazards models with progression free survival (PFS) and overall survival (OS) as clinical endpoints with the results shown in Table 1. In none of the three datasets did the REGS classifier nor predictor developed based on CGP perform better than that developed using HBCCL.

**Table 1:** **Cox proportional hazards analyses of the association between PFS and OS and the classification of the clinical cohorts for doxorubcin REGS developed using HBCCL or CGP cell line panels**

	HBCCL			CGP		
	N	HR (95% CI)	P-value	N	HR (95% CI)	P-value
**REGS classifier (univariate)**						
IDRC (PFS)	470	2.58 (1.72,3.86)	4.37E-06	470	1.54 (1.07,2.22)	0.0211
LLMPP (PFS)	220	2.28 (1.10,4.73)	0.0269	220	1.70 (0.89,3.22)	0.105
MDFCI (OS)	67	4.56 (1.29,16.19)	0.0188	67	0.93 (0.28,3.04)	0.899
**REGS classifier (adjusted for IPI)**						
IDRC (PFS)	424	2.52 (1.64,3.87)	2.65E-05	424	1.46 (0.99,2.16)	0.0562
LLMPP (PFS)	180	2.52 (1.13,5.64)	0.0237	180	1.37 (0.68,2.74)	0.375
MDFCI (OS)	63	4.05 (1.13,14.51)	0.0318	63	0.84 (0.25,2.76)	0.769
**REGS predictor (univariate)**						
IDRC (PFS)	470	1.11 (1.04,1.17)	0.000572	470	1.07 (1.01,1.13)	0.0131
LLMPP (PFS)	220	1.10 (1.00,1.20)	0.0438	220	1.09 (0.99,1.19)	0.0794
MDFCI (OS)	67	1.32 (1.14,1.54)	0.000318	67	0.96 (0.83,1.12)	0.63
**REGS predictor (adjusted for IPI)**						
IDRC (PFS)	424	1.09 (1.03,1.16)	0.00595	424	1.05 (1.00,1.11)	0.0733
LLMPP (PFS)	180	1.11 (1.00,1.23)	0.0433	180	1.04 (0.94,1.15)	0.467
MDFCI (OS)	63	1.34 (1.15,1.57)	0.000237	63	0.95 (0.83,1.07)	0.387

In the multivariate analysis the Cox proportional hazards regression is adjusted for IPI. The estimated HR’s compare patients classified as resistant to patients classified as sensitive.

### Retrospective validation on clinical samples

The HBCCL based REGS classifier for each drug was used to assign each patient of IDRC, LLMPP, and MDFCI a probability of being resistant. Next, the probabilities were combined into a single REGS classifier for CHO by use of Graham’s formula. The patients within each dataset were categorised by tertiles of the range of assigned probabilities for C, H, O, and CHO.

**Figure 3:**
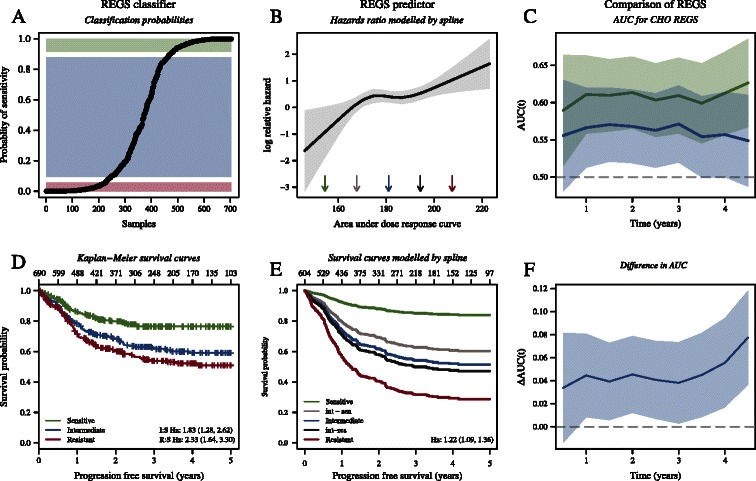
**The association between PFS and the predicted level of sensitivity for the combined REGS for CHO in the merged IDRC and LLMPP cohort.** In panel **A** the probability of being sensitive (one minus the probability of being resistant) according to the REGS classifier is plotted for each patient. Based on the probabilities the patients are categorised into tertiles with those deemed sensitive, intermediate, and resistant indicated by green, blue, and red. Kaplan-Meier curves for PFS are shown in panel **D**. Panel **B** shows estimated log HR versus predicted resistance index modelled by an RCS-model with four knots for the REGS predictor for CHO adjusted for IPI. Panel **E** shows the corresponding survival curves generated by the fitted Cox proportional hazards regression. The survival curves are generated for the values marked by arrows in Panel **B**. Panels **C** and **F** illustrate an analysis of ROC curves for prediction of the combination therapy CHO where all curves are shown with 95% CI. Panel **C** shows AUC under the ROC curves plotted against time for the CHO REGS classifier (green) and predictor (blue). Panel **(F)** shows the difference in AUC plotted against time.

The two large cohorts IDRC and LLMPP were merged into a single dataset and a likelihood ratio test was used to determine that cohort origin was not a significant factor in a Cox proportional hazards model. For CHO the probabilities and Kaplan-Meier curves for the resistance categorisations are shown for the merged IDRC and LLMPP cohort in Figure 3A and D. For MDFCI similar plots are shown in Additional file 2: Figure S5A and B. The categorisations were further analysed using Cox proportional hazards models with PFS and OS as clinical endpoints. The results for the merged IDRC and LLMPP cohort are listed in Table 2. For patients classified as CHO resistant in the merged IDRC and LLMPP cohort, the risk of progression was 2.3 (95% CI: 1.57, 3.37) times greater than for those classified as sensitive when adjusted for IPI. The results for the individual datasets IDRC, LLMPP, and MDFCI are listed in Additional file 2: Table S4, showing that the classifications are of prognostic value in all datasets for H, O, and CHO but not for C solely.

**Table 2:** **Cox proportional hazards analyses of the association between PFS and the predicted level of resistance to the considered drugs in the merged IDRC and LLMPP cohort**

	Univariate (N = 690)		Multivariate (N = 604)	
	HR (95% CI)	P-value	HR (95% CI)	P-value
**REGS classifier**				
CHO	2.33 (1.64,3.30)	2.05e-06	2.30 (1.57,3.37)	1.78e-05
Cyclophosphamide (C)	0.95 (0.70,1.30)	0.764	0.99 (0.71,1.37)	0.949
Doxorubicin (H)	2.52 (1.77,3.59)	2.81e-07	2.55 (1.74,3.72)	1.29e-06
Vincristine (O)	2.07 (1.48,2.88)	2.04e-05	1.69 (1.19,2.40)	0.003
**REGS predictor**				
CHO	1.22 (1.09,1.36)	0.000353	1.22 (1.09,1.37)	0.0009
Cyclophosphamide (C)	0.97 (0.92,1.02)	0.184	0.98 (0.93,1.03)	0.438
Doxorubicin (H)	1.10 (1.05,1.16)	5.53e-05	1.10 (1.04,1.16)	0.0005
Vincristine (O)	1.67 (1.38,2.01)	9.3e-08	1.59 (1.30,1.93)	4.79e-06

In the multivariate analysis the Cox proportional hazards regression is adjusted for IPI. The estimated HR’s for the REGS classifiers compare patients classified as resistant to patients classified as sensitive. In contrast, the estimated HR’s for the REGS predictors are based on an increase of 10 in the predicted *AUC*_*0*_.

For each patient of IDRC, LLMPP, and MDFCI the HBCCL based REGS predictor for each drug was used to assign a resistance index towards each of the three drugs. The REGS predictors for the individual drugs were combined into a single CHO predictor by the geometric mean. Cox proportional hazards models were used to analyse the relationship between the clinical endpoints PFS and OS and the predicted resistance indices. Again the two large cohorts IDRC and LLMPP were merged into a single dataset and a likelihood ratio test was used to determine that cohort origin was not a significant factor. For the merged IDRC and LLMPP cohort the results are shown in Table 2 with PFS as endpoint revealing that an increase in the predicted resistance index of 10 for CHO was related to a 22% (CI: 9%, 37%) increased risk of progression. For the individual drugs an increase in the predicted resistance index of 10 was related to a 10% (CI: 4%, 16%) and 59% (CI: 30%, 93%) increased risk of progression for H and O, respectively. Similarly to the REGS classifier, the REGS predictor for C was not associated with prognosis. In Additional file 2: Table S5 the results of these analyses are listed for the individual datasets. Figure 3B and E show the estimated log relative hazards and associated survival curves for the resistance indices modelled by an RCS and adjusted for IPI with PFS as endpoint for the merged IDRC and LLMPP cohort. For MDFCI a similar plot is shown in Additional file 2: Figure S5 with OS as endpoint.

In order to determine which of the REGS for prediction of resistance to the combination therapy CHO performs best, the AUC of the ROC curves were calculated for the merged IDRC and LLMPP cohort. In terms of two year PFS the AUC of the REGS classifier was 0.61 (CI: 0.56, 0.66) and 0.57 (CI: 0.52, 0.62) for the REGS predictor. The performance of the REGS classifier was significantly better than that of the predictor with a difference in AUC of 0.045 (CI: 0.01, 0.08). For both the REGS classifier and REGS predictor the AUC is plotted against time in Figure 3C and the corresponding difference is shown in Figure 3E. For MDFCI a similar plot is shown in Additional file 2: Figure S5 with OS as endpoint.

### Negative control

The University of Arkansas for Medical Sciences (UAMS) cohort of multiple myeloma patients [37] was used as a negative control. The resistance levels estimated by the REGS classifier for CHO are shown in Additional file 2: Figure S6A. Patients were categorised as sensitive, intermediate, or resistant according to tertiles of these probabilities, and Kaplan-Meier survival curves for the resulting categorisations are shown in Figure S6D. Figure S6B and E show the estimated log relative hazards and associated survival curves for the resistance indices established by the REGS predictors modelled by an RCS with OS as endpoint. The performance of the REGS classifier and predictor is compared in Figure S6C and F by analyses of ROC curves. In summary, none of the REGSs were found capable of predicting OS in this independent cohort.

### Differential expression and GO enrichment in clinical data

First differentially expressed genes between clinical tumours were classified as sensitive or resistant according to the REGS classifier for CHO. Next, the GO terms that were overrepresented in these differentially expressed genes were identified. Finally, the coupling of differentially expressed genes with their GO terms allowed identification of biological differences (Additional file 3). As indicated by the high ranking of GO terms associated with activated immune response (listed from left to right in Additional file 3) the tumours classified as CHO-sensitive had a distinct profile of immune response activation as compared to the resistant ones. Hence, T-cell receptor signalling (LCP2, FYB, FYN, LAT, TRBC1), T-cell cytotoxicity (RAB27A, IL7R, CTSC, IL12RB1, CTSH), target cell apoptosis by cytotoxic T-cells and natural killer cells (GZMB, GZMA, LYZ) and immune surveillance (CD58 and genes of the MHCII class; e.g. HLA-DPA1, HLA-DRB1, HLA-DRA, HLA-DQB1, HLA-DQA1) were up-regulated in the CHO-sensitive tumours. Of these genes the LCP2, CD58, and GZMA were also part of the *in vitro* generated CHO-classifier. Markers of leukocyte migration within or between different tissues and organs of the body were more highly expressed in the CHO-sensitive tumours than in the resistant (CCL2, CCL5, ICAM1, VCAM1, ITGAM, ITGB2) just as members of the Chemokine (C-X-C Motif) Ligand family inducing chemotaxis of T-cells (CXCL9, CXCL10) or B-cells (CXCL13) were up-regulated. This illustrated that the location of tumour cells and their response to chemotherapy is important and affects sensitivity. Genes involved in DNA damage response were differentially up-regulated in the sensitive tumours (FBXO6, RFC5, SOD2) suggesting that preparedness for keeping DNA undamaged promotes sensitivity when encountering a drug that causes DNA damage (CHO) [51-53]. Thus, FBXO6 promotes ubiquitination and degradation of activated CHEK1 [54] that controls cell cycle checkpoints, cell cycle arrest, DNA repair, and cell death to prevent damaged cells from progressing through the cell cycle. Increased levels of CHEK1 in tumour cells may therefore provide them with a survival advantage due to the ability to tolerate a higher level of DNA damage and the tumour cells thereby become chemotherapy resistant [55]. Here we did not observe differences in CHEK1 expression levels among resistant and sensitive tumours. However, the increased expression in sensitive tumours of FBXO6, RFC5 that mediates elongation of primed DNA templates by DNA polymerase [56] and SOD2 that prevents DNA oxidation damage by destroying superoxide anion radicals [57] support that the degree of undamaged DNA associates with better chemotherapy sensitivity.

## Discussion

The aim of the article was to establish a workflow for generating REGSs capable of predicting the clinical outcome of cancer patients. The workflow consists of *in vitro* drug screens and microarray data of drug naïve carefully selected cancer cell lines combined with regularised multivariate regression. To exemplify the workflow, REGS were developed for patients suffering from the haematological malignancy DLBCL treated with R-CHOP first line therapy. To do this, gene expression data were established for the HBCCL panel and each cell line were tested successively for resistance toward C, H, and O at least in triplicates.

REGS for H developed using HBCCL was initially compared to REGS developed using CGP [15]. The REGS classifiers for H based on HBCCL and CGP contained 118 and 88 genes, respectively, of which the 7 genes: CDKN2A, KCTD12, DPYD, MEST, TIMP2, VIM, and RIMS3 were in common. The REGS predictors for H based on HBCCL and CGP utilised 52 and 141 genes, respectively, of which the following six were in common: CDKN2A, DPYD, GLUL, NRN1, TIMP2, and VIM. The performance of the REGSs was compared using the clinical cohorts IDRC (n = 470), LLMPP (n = 233), and MDFCI (n = 67) consisting of patients treated with R-CHOP first line therapy. In none of the performed analyses did the REGS based on CGP perform better than those established using HBCCL. Consequently, it seems that REGSs developed using carefully selected origin specific cell lines analysed to the requirements of [19] can be superior in prognostic value as compared to those developed using large-scale studies covering many different cancer forms [15-18].

In order to generate REGS for the combination therapy CHO, the individual drugs C and O were also screened using the HBCCL panel. The regularised logistic regression resulted in REGS classifiers utilising 73 genes for C and 32 for O and regularised linear regression resulted in REGS predictors utilising 27 genes for C and 21 for O. The REGS classifiers for C, H, and O were combined using Graham’s formula whereas the REGS predictors were combined by the geometric mean, as these do not generate probabilities. The REGSs were evaluated for predictive power in clinical cohorts with PFS and OS used as surrogate endpoints for the efficacy of R-CHOP. The classifier and predictor based REGSs for CHO, H, and O were able to predict patient outcome, whereas predictions based on C alone were not. Thus, despite the fact that the roles of the antibody rituximab and the steroid prednisolone have not been taken into account, the REGS for CHO generated predictions significantly associated with prognosis. By use of time dependent ROC curves the performance of the REGS classifier for CHO were found significantly better than that of the REGS predictor. Finally, neither of the established REGSs were able to predict outcome in multiple myeloma patients (UAMS cohort) suggesting that the established REGSs were treatment-specific.

Lack of successful REGS-cyclophosphamide (C) classification could depend on absence of stromal cells in our *in vitro* screening model since it has recently been observed that the cytotoxic mechanism of cyclophosphamide might be through release of stress-related cytokines from the malignant cells, thereby attracting macrophages into the microenvironment of the tumour and potentiating antibody-specific killing [58]; properties that were not included in our *in vitro* dose response experiments and perhaps explain why our REGS classifier for C of clinical *de novo* DLBCL cases did not possess prognostic impact.

### Methodological considerations

Despite of the controversy associated with the development of *in vitro* REGSs for prediction of resistance to chemotherapeutics [8], we were still encouraged to explore the possibility of establishing cell line derived REGSs for prediction of the efficacy of C, H, and O in DLBCL. Contrary to earlier undertakings of the challenge the dose response curves in our study were established using a method that accounts for the individual cell lines’ growth rate and *AUC*_*0*_ was used as summary statistic. This may have aided generation of the successful REGSs with prognostic potential.

The Affymetrix Genechip HG-U133 Plus 2.0 typically contains several probe sets probing the same gene. Handling these redundant probe sets requires careful consideration. However, as studied by Bourgon et al. [59] independent filtering can increase detection power in high-throughput experiments. Similar to [4,60] we chose the most variable probe set for each gene as a representative in this article.

Due to the high dimensionality of the Affymetrix Genechip HG-U133 Plus 2.0 array, genes that contribute only noise to the clinical predictions are unavoidable. To minimise the influence of such genes the elastic net penalty was used to generate sparse REGSs with the number of contributing genes chosen by leave-one-out cross validation.

In our study, the generated REGSs did not have predictive power for C. Hence, these REGSs may require use of other toxicity measures than *AUC*_*0*_ reflecting other biological mechanisms. As drug screen assays used for the dose response experiments depends on inhibition of cell proliferation, other biological functions such as apoptosis and DNA repair are likely to be involved in the efficacy of the drugs. Optimally this should be taken into account when developing REGSs based on other mechanisms of out-read parameters.

The established REGS classifier and predictor for CHO were generated using equal weights for the three individual drugs since dose response screens were performed for individual drugs only and not in combination. Most likely, the performance could be optimised taking into account experimental based weighting schemes where the impact of C, H, and O were ranked individually and where the effects of drug combinations were determined.

The REGS predictors were combined by the geometric mean of the resistance indices. The geometric mean was used, as opposed to the arithmetic mean, because the resistance indices for the three drugs differed in scale. The REGS classifiers were combined using Graham’s formula under the assumption of independence. Other methods for combining probabilities of resistance have previously been considered. Also under the assumption of independent drug effects, Havaleshko et al. [11] suggested combining the probabilities as *P*_*A*_*P*_*B*_ where *P*_*A*_ and *P*_*B*_ are the probabilities of being resistant towards two drugs: A and B. However, as noted by the authors, the resulting probabilities are optimistic. Say the probability of being resistant to drugs A and B is 0.5, then the combined probability of resistance is 0.25. Liedtke et al. [12] suggested to evaluate each drug independently and summarise the result by counting the number of drugs a patient was resistant towards. However, this method disregards the numeric value of the probability, i.e. a probability of 0.51 is given the same weight as 0.99.

The use of logistic regression resulted in REGS classifiers capable of estimating the probability of a patient being sensitive or resistant to each of the three drugs. This can be done for multiple drugs making it possible to find those towards which the patient is most likely to be sensitive. However, to use logistic regression the cell lines were categorised into three groups and only the most sensitive and resistant were used to develop the classifier. Furthermore, the numeric value of the summary statistic *AUC*_*0*_ was discarded in favour of a dichotomous sensitivity score. As described by Royston et al. [61] such an approach may be suboptimal as not all information is utilised, which supported our strategy to also include REGS predictors exploiting the continuum of dose response data. REGS predictors based on linear regression utilises all of the available cell lines without categorising them into groups. However, assignments made from such REGSs do not result in estimates of the probability of patients being sensitive or resistant. Instead the REGSs are used to generate an index for which high numbers are associated with resistance and low numbers with sensitivity. This problematises comparison of individual patients’ resistance levels toward multiple drugs and thereby finding those most likely to be beneficial. Nevertheless, for whole cohorts of patients it is possible to find the drugs where the individual patient is predicted to be within the group of most sensitive. Thus, for cohorts of patients, the REGS predictors also point to the drugs that most likely will be beneficial for the given patient.

Both logistic and linear regressions are thus associated with strengths and weaknesses. Hence, both regression methods were used and compared in this paper. The predictions based on the two approaches were investigated using time dependent ROC curves for cumulative PFS. Logistic regression performed significantly better than linear regression prompting us to encourage the use of regularised logistic regression for developing REGS because of increased performance and interpretation.

## Conclusion

REGSs were developed for the purpose of predicting response to C, H, O, and CHO using a focused B-cell cell line panel and regularised multivariate regression techniques. By applying these REGSs to clinical cohorts with known outcomes, we found that REGSs developed for CHO, H and O, but not C, were capable of predicting a higher risk of disease progression and death. The clinical perspectives of the present study are encouraging. Although the REGS method is far from implication in clinical practice the possibility of predicting treatment response before first cycle of chemotherapy would represent a paradigm shift. The advantages are obvious: 1) currently used clinical risk stratification in DLBCL (IPI) predicts outcome of groups of patients and not for individual patients, 2) early PET/CT assessed chemosensitivity and tumour cell kill after few cycles of chemotherapy is not optimal as patients receive two to three cycles of toxic therapy before awareness of refractoriness. REGSs could potentially enable effective treatment for the individual patient from the beginning.

## Additional files

Additional file 1: Text S1. KnitR document containing the analysis conducted in R.
Additional file 2:
**Contains supplementary figures and tables.**

Additional file 3: **A table containing all genes differentially expressed between CHO resistant and sensitive patients with a log 2 fold change exceeding 1.** The table also contains GO terms where the genes are significantly overrepresented. These GO terms are arranged from left to right according to descending significance.

## Abbreviations

AUC: Area under the curve
AUC_0_: Area under the curve above 0
C: Cyclophosphamide
CCLE: Cancer Cell Line Encyclopedia
CGP: Cancer genome project
CI: 95% confidence interval
DLBCL: Diffuse large B-cell lymphoma
GEP: Gene expression profiles
IDRC: The International DLBCL Rituximab-CHOP Consortium MD Anderson
IPI: The international prognostic index
H: Doxorubicin
HBCCL: Human B-cell cancer cell lines
LDA: Linear discriminant analysis
LLMPP: The Lymphoma/Leukemia Molecular Profiling Project R-CHOP
MDFCI: The Mayo-Dana-Farber Cancer Institute
MM: Multiple myeloma
MSPE: Mean square prediction error
NCI: National cancer institute
O: Vincristine
OS: Overall survival
P: Prednisolone
PFS: Progression free survival
R: Rituximab
REGS: Resistance gene signature
ROC: Receiver operating characteristic
