# Supplementary material for: Predicting response to multidrug regimens in cancer patients using cell line experiments and regularised regression models
Source: BMC Cancer. 2015 Apr 8;15:235. doi: 10.1186/s12885-015-1237-6 (PMC4396063; doi:10.1186/s12885-015-1237-6)
Supplement: Additional file 1: Text S1. — KnitR document containing the analysis conducted in R. [file 12885_2015_1237_MOESM1_ESM.pdf]

Text S1: Knitr Document

Predicting Response to Multidrug Regimens in Cancer Patients using Cell Line Experiments and Regularised Regression Models

February 8, 2015

Authors

Steffen Falgreen, Karen Dybkær, Ken H. Young, Zijun Y. Xu-Monette, Tarec C. El-Galaly, Maria Bach Laursen, Julie S. Bødker, Malene K. Kjeldsen, Alexander Schmitz, Mette Nyegaard, Hans Erik Johnsen, Martin Bøgsted

Contents

I Generation of REGS 4

1 R settings 5
1.1 Library Acquisitions . . . 5
1.2 R-session Information . . . 5

2 GCP Doxorubicin 6
2.1 RMA normalisation of GEPdata . . . 6
2.2 Filtering the GCP Expression Data . . . 6
2.3 Establishment of REGS Predictor . . . 7
2.3.1 Cross Validation . . . 7
2.4 Establishment of REGS Classifier . . . 9
2.4.1 Cross Validation . . . 9

3 Load HBCCL GEP Data 13
3.1 Filtering the HBCCL Expression Data . . . 13
3.2 Scaling of Gene Expression Data . . . 14

4 Cyclophosphamide 15
4.1 Load Cyclophosphamide Dose Response Data . . . 15
4.2 Establishment of REGS Predictor . . . 17
4.2.1 Cross Validation . . . 17
4.3 Establishment of REGS Classifier . . . 20
4.3.1 Cross Validation . . . 20

5 Doxorubicin 24
5.1 Load Doxorubicin Dose Response Data . . . 24
5.2 Establishment of REGS Predictor . . . 26
5.2.1 Cross Validation . . . 26
5.3 Establishment of REGS Classifier . . . 29
5.3.1 Cross Validation . . . 29

6 Vincristine 33
6.1 Load Vincristine Dose Response Data . . . 33
6.2 Establishment of REGS Predictor . . . 35
6.2.1 Cross Validation . . . 35
6.3 Establishment of REGS Classifier . . . 38
6.3.1 Cross Validation . . . 38

II Validation 42

7 IDRC Cohort 44
7.1 Loading the data . . . 44
7.2 REGS Classification . . . 44
7.2.1 Cox Proportional Hazards Regression . . . 45
7.2.2 Kaplan-Meier Analysis . . . 45
7.3 REGS Prediction . . . 48
7.3.1 Cox Proportional Hazards Regression . . . 48

7.3.2 Cox Proportional Hazards Regression based on Restricted Cubic Splines 48

8 LLMPP Cohort **51**
8.1 Loading the data 51
8.2 REGS Classification 51
8.2.1 Cox Proportional Hazards Regression 52
8.2.2 Kaplan-Meier Analysis 52
8.3 REGS Prediction 55
8.3.1 Cox Proportional Hazards Regression 55
8.3.2 Cox Proportional Hazards Regression based on Restricted Cubic Splines 55

9 IDRC and LLMPP Combined **58**
9.1 Cox proportional hazard rate ratios 58
9.2 Kaplan Meier Analysis 60
9.3 Cox Proportional Hazards Regression based on Restricted Cubic Splines 62
9.4 Comparison of REGS Classifiers and Predictors 64

10 MDFCI Cohort **67**
10.1 Loading the data 67
10.2 REGS classification 67
10.2.1 Cox Proportional Hazards Regression 68
10.2.2 Kaplan Meier Analysis 68
10.3 REGS Predictors 71
10.3.1 Cox Proportional Hazards Regression 71
10.3.2 Cox Proportional Hazards Regression based on Restricted Cubic Splines 72
10.4 Comparison of REGS Classifiers and Predictors 74

11 UAMS Cohort **76**
11.1 Loading the data 76
11.2 REGS classification 76
11.2.1 Cox Proportional Hazards Regression 77
11.2.2 Kaplan Meier Analysis 77
11.3 REGS Predictors 80
11.3.1 Cox Proportional Hazards Regression 80
11.3.2 Cox Proportional Hazards Regression based on Restricted Cubic Splines 80
11.4 Comparison of REGS Classifiers and Predictors 82

12 Comparison of GCP and HBCCL **85**
12.1 Cox Proportional Hazards Regression 85

13 Differentially Expressed Genes and GO Enrichment **88**
13.1 Differentially Expression in Clinical Data 88
13.2 GO enrichment 89
13.2.1 GO Enrichment, Step by Step 90

Part I

Generation of REGS

1 R settings

Load the annotation data for the Affymetrix GeneChip Human Genome U133 Plus 2 array.

```
load(file.path(Database, "Annotation/GeneratedData/HGU133Plus2.na33.annot.RData"))
```

1.1 Library Acquisitions

The libraries which are required for the analysis are loaded below

```
require(Biobase)
require(annotate)
require(affy)
require(genefilter)
require(survival)
require(glmnet)
require(DoseR)
require(timeROC)
require(pracma)
require(hthgu133a.db)
require(hgu133plus2.db)
require(VennDiagram)
library(GOstats)
library(GO.db)
library(limma)
```

1.2 R-session Information

Information regarding the R-session is printed below

```
toLatex(sessionInfo())
```

- R version 3.1.2 (2014-10-31), `x86_64-apple-darwin13.4.0`
- Locale: `da_DK.UTF-8/da_DK.UTF-8/da_DK.UTF-8/C/da_DK.UTF-8/da_DK.UTF-8`
- Base packages: base, datasets, graphics, grDevices, grid, methods, parallel, splines, stats, stats4, tcltk, tools, utils
- Other packages: affy 1.44.0, animation 2.3, annotate 1.44.0, AnnotationDbi 1.28.1, Biobase 2.26.0, BiocGenerics 0.12.1, BiocInstaller 1.16.1, bitops 1.0-6, Category 2.32.0, DBI 0.3.1, DoseR 0.1, extrafont 0.17, foreign 0.8-62, Formula 1.1-2, gdata 2.13.3, genefilter 1.48.1, GenomeInfoDb 1.2.4, glmnet 1.9-8, GO.db 3.0.0, GOstats 2.32.0, graph 1.44.1, hgu133plus2.db 3.0.0, Hmisc 3.14-6, hthgu133a.db 3.0.0, IRanges 2.0.1, knitr 1.8, lattice 0.20-29, limma 3.22.3, Matrix 1.1-4, mvtnorm 1.0-2, nlme 3.1-119, org.Hs.eg.db 3.0.0, pec 2.3.7, plyr 1.8.1, pracma 1.7.9, prada 1.42.0, RColorBrewer 1.1-2, RCurl 1.95-4.5, RJSONIO 1.3-0, robustbase 0.92-2, rrcov 1.3-8, RSQLite 1.0.0, S4Vectors 0.4.0, shiny 0.10.2.2, shinyIncubator 0.2.2, survival 2.37-7, timereg 1.8.6, timeROC 0.2, VennDiagram 1.6.9, WriteXLS 3.5.1, XML 3.98-1.1
- Loaded via a namespace (and not attached): acepack 1.3-3.3, affyio 1.34.0, AnnotationForge 1.8.1, cluster 1.15.3, codetools 0.2-9, DEoptimR 1.0-2, digest 0.6.8, evaluate 0.5.5, extrafontdb 1.0, foreach 1.4.2, formatR 1.0, GSEABase 1.28.0, gtools 3.4.1, highr 0.4, htmltools 0.2.6, httpuv 1.3.2, iterators 1.0.7, latticeExtra 0.6-26, lava 1.3, MASS 7.3-37, mime 0.2, nnet 7.3-8, pcaPP 1.9-60, preprocessCore 1.28.0, prodlim 1.5.1, R6 2.0.1, RBGL 1.42.0, Rcpp 0.11.3, rpart 4.1-8, Rttf2pt1 1.3.2, stringr 0.6.2, xtable 1.7-4, zlibbioc 1.12.0

2 GCP Doxorubicin

Gene expression data on the Affymetrix Genechip HG-U133A array was obtained from ArrayExpress under accession number E-MTAB-783. The CGP dose response data for doxorubicin contained in the file gdsc_manova_input_w2.csv were downloaded from the CGP website: www.cancerrxgene.org.

Determine directories for output.
The directories storing R-output are created.
First the metadata for the GCP doxorubicin is loaded.

```
load(GCPmetadata.file)
```

The metadata contains the following information:

```
head(metadataGCP)

##        Cell.line Cosmic.ID                              Tissue Cancer.type
## PFSK-1    PFSK-1    683667                    medulloblastoma         CNS
## A673        A673    684052 rhabdomyosarcoma (putative Ewing's) soft tissue
## ES3          ES3    684055                      Ewings sarcoma        bone
## ES5          ES5    684057                      Ewings sarcoma        bone
## ES7          ES7    684059                      Ewings sarcoma        bone
## EW-11      EW-11    684062                      Ewings sarcoma        bone
##           IC50     AUC                        Cel.file
## PFSK-1 -0.3885 0.83275 5500024032848101507000.D07.CEL
## A673   -1.5334 0.71353 5500024032848101507998.A03.CEL
## ES3    -2.4138 0.60379 5500024034290101707049.A06.CEL
## ES5    -3.2385 0.62409 5500024034290101707049.C06.CEL
## ES7    -2.9680 0.54209 5500024034290101707049.E06.CEL
## EW-11  -1.0742 0.76802 5500024034290101707049.H06.CEL
```

2.1 RMA normalisation of GEPdata

The following code chunk performs RMA pre-processing of the GEP data from GCP. The RMA pre-processing is only performed if it is not already done.

```
if(file.exists(GEPGCP.file)){
    load(GEPGCP.file)
}else{

  GEPGCP <- just.rma(filenames = filenames)

  sampleNames(GEPGCP) <- metadataGCP$Cell.line

  save(GEPGCP, file = GEPGCP.file)
}
```

2.2 Filtering the GCP Expression Data

Only probe sets which are associated with an ensemble gene ID are used for the analysis. Furthermore, for genes represented by multiple probe-sets only the probeset with the highest standard deviation is used.

```
var.probes <-featureNames(nsFilter(GEPGCP, var.func= stats:::sd,
                                   remove.dupEntrez = TRUE, var.cutoff = 0,
                                   filterByQuantile = FALSE)$eset)

GEPGCP.sc <- GEPGCP[var.probes, ]
```

Finally the GEP data is median centred.

```
MedianCenter <- function(x) {
  rowMedians <- apply(x, 1, function(x) median(x, na.rm = TRUE))
  return(x - matrix(rep(rowMedians, ncol(x)), nrow(x), ncol(x)))
}

exprs(GEPGCP.sc) <- MedianCenter(exprs(GEPGCP.sc))
```

2.3 Establishment of REGS Predictor

2.3.1 Cross Validation

```
drug <- "Doxorubicin"

file.Doxorubicin.predict <-
  file.path("GeneratedData/REGS", paste("GCP_Doxorubicin_prediction_genes.RData"))

if(file.exists(file.Doxorubicin.predict)){
  load(file.Doxorubicin.predict)
}else{

  set.seed(10000)

  ## Create a matrix to store the results
  mat <- matrix(NaN, ncol = 4, nrow = length(alphas))
  colnames(mat) <- paste("Doxorubicin",
                         c("", ".lo", ".up", ".n"), sep = "")
  row.names(mat) <- alphas

  ## plot all the cross validation plots for later inspection
  pdf(file.path("Output", "cvplots_prediction.pdf"))

  pred.Doxorubicin.fit <- list()

  metadataGCP <- metadataGCP[!is.na(metadataGCP$AUC), ]
  int <- intersect(sampleNames(GEPGCP.sc), row.names(metadataGCP))

  ## The GEP data is aligned with the dose response
  train.set <- exprs(GEPGCP.sc[,int])

  ## The AUC0 values are extracted from the matrix
  y <- metadataGCP[int, "AUC", drop = FALSE]

  ## The cross validation is conducted for the supplied alpha values
  for(alpha in alphas){
    cat(alpha, "\n")
    fit <-
      cv.glmnet(x       = t(train.set[, rownames(y)]),
                y       = y[,1],
                standardize = FALSE,
                alpha   = alpha,
                nlambda = 600,
                nfolds  = 20,
                keep    = TRUE,
                grouped = FALSE)
```

```
    ## plot the fit

    plot(fit, main = paste(drug, alpha))

    ## find the minimum and save upper and lower boundary
    wh.min <- which.min(fit$cvm)
    mat[paste(alpha), drug] <- min(fit$cvm)
    mat[paste(alpha), paste(drug, ".lo", sep = "")] <- fit$cvlo[wh.min]
    mat[paste(alpha), paste(drug, ".up", sep = "")] <- fit$cvup[wh.min]
    mat[paste(alpha), paste(drug, ".n",  sep = "")] <- fit$nzero[wh.min]

    ## add the cv fit to the list of fits
    pred.Doxorubicin.fit[[paste(alpha)]] <-fit
  }
  dev.off()
  save(pred.Doxorubicin.fit, mat, file = file.Doxorubicin.predict)
}
```

The best fit is found in the code chunck below:

```
GCPDoxorubicinPredictor.cv <-
      pred.Doxorubicin.fit[[names(which.min(mat[paste(alphas), drug]))]]
```

The result of the cross validation is plotted in Figure 2.1. The code chunk below creates this plot.

```
par(mfrow = c(1, 3))

plot.cv.alpha(x  = as.numeric(row.names(mat)),
              y  = mat[, drug],
              lo = mat[,    paste(drug, ".lo", sep = "")],
              up = mat[,    paste(drug, ".up", sep = "")],
              nzero = mat[, paste(drug, ".n",  sep = "")],
              las   = 1,
              main1 = drug)

mtext("(a)", 3,
      line=1.5, adj=-0.145, cex=9/pointsize)

plot.cv.lambda(x  = log(GCPDoxorubicinPredictor.cv$lambda),
               y  = GCPDoxorubicinPredictor.cv$cvm,
               lo = GCPDoxorubicinPredictor.cv$cvlo,
               up = GCPDoxorubicinPredictor.cv$cvup,
               nzero = GCPDoxorubicinPredictor.cv$nzero,
               las   = 1,
               main1 = drug)

mtext("(b)", 3,
      line=1.5, adj=-0.145, cex=9/pointsize)

plot.coef(GCPDoxorubicinPredictor.cv,
          label.pct= 40,
          main = expression(paste("Regularisation Curves for Doxorubicin")))

mtext("(c)", 3,
      line=1.5, adj=-0.145, cex=9/pointsize)
```

The minimum mean squared error for Doxorubicin was 0.03, which was obtained at α =0.85 and $\log(\lambda)$ =−4.23. The resulting GEP classifier for these parameters have non null coefficients for 141 genes. For the α value resulting in the smallest cross validation error, the regularisation curves are plotted in Figure 2.1 Panel C.
The REGS predictor for doxorubicin is established below.

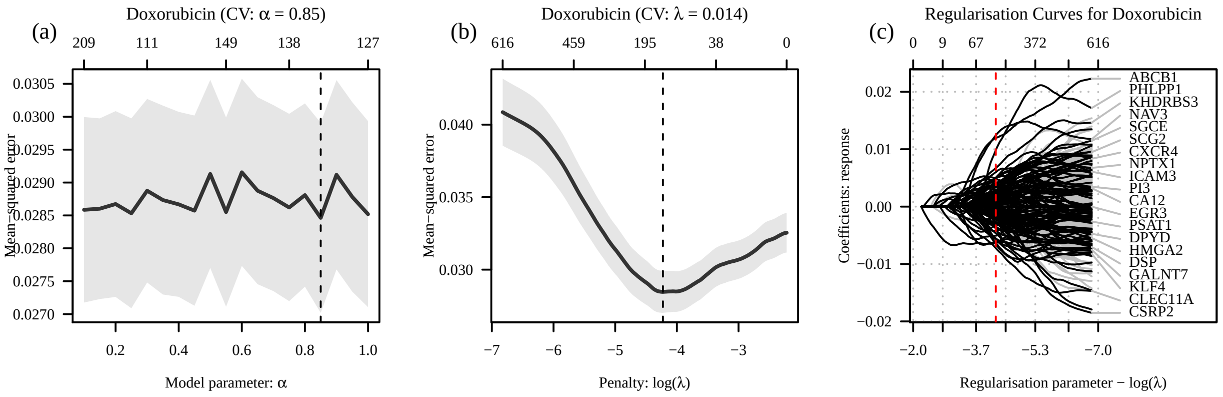

Figure 2.1: Cross validation and regularisation curves. In panel A the minimum mean squared error obtained for various α values are plotted. In panel B the mean-squared error is plotted against λ for the α value resulting in the minimum error and in Panel C the corresponding regularisation curves are shown. The genes for the 20 probe-sets associated with the largest coefficients are shown.

```
COEF <- as.matrix(coef(GCPDoxorubicinPredictor.cv, s = "lambda.min"))

Doxorubicin.reisitance.coef <- COEF[COEF != 0, ]

probes <- names(Doxorubicin.reisitance.coef)[c(-1)]

sd.Doxorubicin <- apply(exprs(GEPGCP.sc[probes, ]), 1, function(x) sd(x, na.rm = TRUE))

GCPDoxorubicin.reisitance.pred.coef <-
  Doxorubicin.reisitance.coef * c(1, sd.Doxorubicin)

GCPDoxorubicinPredictor<- function(newx, coef = GCPDoxorubicin.reisitance.pred.coef){

  x <- rbind(1, newx[names(coef)[-1],,drop= FALSE])

  t(x) %*% as.matrix((coef))
}
```

The REGS predictor is saved for later use.

```
save(GCPDoxorubicinPredictor, GCPDoxorubicin.reisitance.pred.coef,
     file = "GeneratedData/REGS/GCPDoxorubicinPredictor.RData")
```

2.4 Establishment of REGS Classifier

```
cutFun <-function(x) {
  cut(x,
      breaks=c(-Inf, quantile(x, c(1/3, 2/3),na.rm = TRUE), Inf),
      labels = c("Sensitive", "Intermediate","Resistant"))
}
```

2.4.1 Cross Validation

```
drug <- "Doxorubicin"
file.Doxorubicin.class <-
  file.path("GeneratedData/REGS",
```

```r
            paste("GCP_Doxorubicin_classification_genes.RData"))

if(file.exists(file.Doxorubicin.class)){
  load(file.Doxorubicin.class)
}else{

  set.seed(10000)

  mat <- matrix(NaN, ncol = 4, nrow = length(alphas))
  colnames(mat) <- paste("Doxorubicin",
                         c("", ".lo", ".up", ".n"), sep = "")
  row.names(mat) <- alphas

  ## plot all the cross validation plots for later inspection
  pdf(file.path(Doxorubicin.output, "cvplots_classification.pdf"))

  class.Doxorubicin.fit <- list()

  ## The GEP data is aligned with the dose response
  metadataGCP <- metadataGCP[!is.na(metadataGCP$AUC), ]
  int <- intersect(sampleNames(GEPGCP.sc), row.names(metadataGCP))

  ## The GEP data is aligned with the dose response
  train.set <- exprs(GEPGCP.sc[,int])

  ## The AUC values are extracted from the matrix
  y <- cutFun(metadataGCP[int, "AUC"])
  names(y) <- int
  y <- factor(y[y != "Intermediate"], levels = c("Sensitive", "Resistant"))


  for(alpha in alphas){
    cat(drug, alpha, "\n")

    fit <-
      cv.glmnet(x = t(train.set[,names(y)]),
                alpha        = alpha,
                grouped      = FALSE,
                type.measure = "class",
                family       = "multinomial",
                nfolds       = 20,
                keep         = TRUE,
                y            = y,
                lambda       = rev(exp(seq(-6, 3, length.out = 600))),
                standardize  = FALSE,
                nlambda      = 600)



    plot(fit, main = paste(drug, alpha))

    wh.min <- length(fit$cvm) - which.min(rev(fit$cvm))
    mat[paste(alpha), drug] <- min(fit$cvm)
    mat[paste(alpha), paste(drug, ".lo", sep = "")] <-
      fit$cvlo[wh.min]
    mat[paste(alpha), paste(drug, ".up", sep = "")] <-
      fit$cvup[wh.min]
    mat[paste(alpha), paste(drug, ".n", sep = "")] <-
      fit$nzero[wh.min]
```

```
    class.Doxorubicin.fit[[paste(alpha)]] <- fit

  }

  dev.off()
  save(class.Doxorubicin.fit, mat, file = file.Doxorubicin.class)
}
```

The alpha value resulting in the smallest classication error is found in the code chunk below.

```
fit <- class.Doxorubicin.fit[[names(which.min((mat[, "Doxorubicin"])))]]
fit$lambda.min <-
  rev(fit$lambda)[which.min(rev(fit$cvm))]
GCPDoxorubicinClassifier.cv <- fit
```

The result of the cross validation is plotted in Figure 2.2. The code chunk below creates this plot.

```
par(mfrow = c(1,3))
plot.cv.alpha(x  = as.numeric(row.names(mat)),
              y  = mat[, drug],
              lo = mat[, paste(drug, ".lo", sep = "")],
              up = mat[, paste(drug, ".up", sep = "")],
              nzero = mat[,paste(drug, ".n", sep = "")],
              main1 = drug,
              las   = 1,
              ylab = "Misclassification error")

mtext("(a)", 3,
      line=1.5, adj=-0.145, cex=9/pointsize)

plot.cv.lambda(x  = log(GCPDoxorubicinClassifier.cv$lambda),
               y  = GCPDoxorubicinClassifier.cv$cvm,
               lo = GCPDoxorubicinClassifier.cv$cvlo,
               up = GCPDoxorubicinClassifier.cv$cvup,
               nzero = GCPDoxorubicinClassifier.cv$nzero,
               main1 = drug,
               xlim  = c(-6, 0),
               las   = 1,
               ylab = "Misclassification error")

mtext("(b)", 3,
      line=1.5, adj=-0.145, cex=9/pointsize)
plot.coef(GCPDoxorubicinClassifier.cv, xlim = c(0,-6),
          label.pct=33,
          main = paste("Regularisation curves for", drug))

mtext("(c)", 3,
      line=2, adj=-0.145, cex=9/pointsize)
```

The minimum mean squared error for Doxorubicin was 0.31, which was obtained at α =0.45 and $\log(\lambda)$ =–2.21. The resulting GEP classifier for these parameters have non null coefficients for 88 genes. For the α value resulting in the smallest cross validation error, the regularisation curves are plotted in Figure 2.2 Panel C.

The REGS classifier for doxorubicin is established below.

```
COEF <- as.matrix(coef(GCPDoxorubicinClassifier.cv, s = "lambda.min")$Resistant)

GCPDoxorubicinClassifier.coef <- COEF[COEF != 0, ]
```

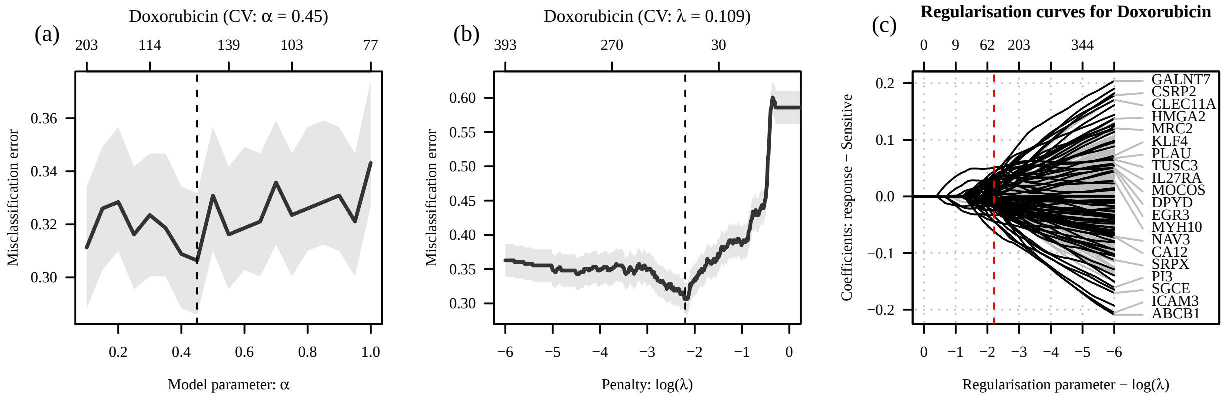

Figure 2.2: Cross validation and regularisation curves. In panel A the misclassification error obtained for various α values are plotted. In panel B the misclassification error is plotted against λ for the α value resulting in the minimum error and in Panel C the corresponding regularisation curves are shown. The genes for the 20 probe-sets associated with the largest coefficients are shown.

```
probes <- names(GCPDoxorubicinClassifier.coef)[-1]

sd.Doxorubicin <- apply(exprs(GEPGCP.sc[probes, ]), 1, function(x) sd(x, na.rm = TRUE))

GCPDoxorubicin.reisitance.class.coef <- GCPDoxorubicinClassifier.coef * c(1, sd.Doxorubicin)

GCPDoxorubicinClassifier <- function(newx, coef = GCPDoxorubicin.reisitance.class.coef){

  x <- rbind(1, newx[names(coef)[-1],,drop= FALSE])

  prob <- t(x) %*% as.matrix((coef)  )

  prob <- exp(prob) / ( exp(prob) + exp(-prob))
  colnames(prob) <- "Resistant"
  prob
}
```

The REGS classifier is saved for later use.

```
save(GCPDoxorubicinClassifier, GCPDoxorubicin.reisitance.class.coef,
     file = "GeneratedData/REGS/GCPDoxorubicinClassifier.RData")
```

3 Load HBCCL GEP Data

The Human B-cell Cancer Cell Line (HBCCL) panel consists of 26 cancer cell lines all originating from patients suffering from a B-cell malignancy. Affymetrix GeneChip HG-U133 Plus 2.0 arrays (HG-U133 Plus 2.0) have been used to obtain the gene expression data of the cell lines.
The gene expression data have been deposited at

- http://www.ncbi.nlm.nih.gov/geo/

under GEO accession number GSE53798.

Functions used for centering and scaling of microarray data.

```
MedianCenter <- function(x) {
  rowMedians <- apply(x, 1, function(x) median(x, na.rm = TRUE))
  return(x - matrix(rep(rowMedians, ncol(x)), nrow(x), ncol(x)))
}

SdScaling <- function(x, y) {
  rowSdx <- apply(x, 1, function(x) sd(x, na.rm = TRUE))
  rowSdy <- apply(y, 1, function(x) sd(x, na.rm = TRUE))
  return( (x / matrix(rep(rowSdx, ncol(x)), nrow(x), ncol(x)))
          * (matrix(rep(rowSdy, ncol(x)), nrow(x), ncol(x))))
}
```

Load metadata for the HBCCL panel.

```
load(file.path(HBCCL.dir, "GeneratedData/Metadata/metadataHBCCL.RData"))

metadataHBCCL$Cellline <- as.character(metadataHBCCL$Cellline)
```

Determine the names of the cell lines to be used.

```
names <- metadataHBCCL$Cellline[metadataHBCCL$Disease %in%
                                  c("MM", "DLBCL")]

names.MM <- metadataHBCCL$Cellline[metadataHBCCL$Disease == "MM"]


names.DLBCL <- metadataHBCCL$Cellline[metadataHBCCL$Disease == "DLBCL"]
```

Load the RMA normalised GEP data.

```
load(file.path(HBCCL.dir, "GeneratedData/PreProcessed/GEPCHO_RMA_affy.RData"))
```

3.1 Filtering the HBCCL Expression Data

Only probe sets which are associated with an ensemble gene ID are used for the analysis. Furthermore, for genes represented by multiple probe-sets only the probeset with the highest standard deviation is used.

```
DLBCL <- as.character(metadataHBCCL[metadataHBCCL$CHO_HGU133Plus2 == 1 &
                                      metadataHBCCL$Disease == "DLBCL",
                          "Cellline"])

MM <- as.character(metadataHBCCL[metadataHBCCL$CHO_HGU133Plus2 == 1 &
                                   metadataHBCCL$Disease == "MM",
                       "Cellline"])
```

```r
var.probes <-featureNames(nsFilter(GEPCHO[ ,DLBCL], var.func= stats:::sd,
                                   remove.dupEntrez = TRUE, var.cutoff = 0,
                                   filterByQuantile = FALSE)$eset)
```

This results in 20155 probe-sets that are used in the analysis.

3.2 Scaling of Gene Expression Data

In order to make the HBCCL GEP data comparable to other datasets it is median centered. This is done for the DLBCL GEP data first.

```r
GEPDLBCL <- GEPCHO[,sampleNames(GEPCHO) %in% DLBCL]

GEPDLBCL.med <- GEPDLBCL[var.probes, ]
exprs(GEPDLBCL.med) <- MedianCenter(exprs(GEPDLBCL.med))
```

The MM cell line data is scaled according to the DLBCL cell line data

```r
GEPMM.sc <- GEPCHO[var.probes, sampleNames(GEPCHO) %in% MM]
exprs(GEPMM.sc) <- MedianCenter(exprs(GEPMM.sc))
exprs(GEPMM.sc) <- SdScaling(exprs(GEPMM.sc),
                             exprs(GEPDLBCL.med))
```

Finally, the two data datasets are combined

```r
GEPCHO.sc <- GEPCHO[var.probes, sampleNames(GEPCHO)]
exprs(GEPCHO.sc) <-
  cbind(exprs(GEPMM.sc), exprs(GEPDLBCL.med))[, sampleNames(GEPCHO.sc)]
```

4 Cyclophosphamide

4.1 Load Cyclophosphamide Dose Response Data

Set seed to ensure reprodicible research.

```
set.seed(1000)
```

Determine directories for output.

```
Cyclophosphamide.figure  <- file.path("Output", "Cyclophosphamide", "Figures/")
Cyclophosphamide.table   <- file.path("Output", "Cyclophosphamide", "Tables/")
Cyclophosphamide.output  <- file.path("Output", "Cyclophosphamide", "Prediction/")
```

The directories storing R-output are created.

```
dir.create(path = file.path(Cyclophosphamide.figure),
           showWarnings = FALSE, recursive = TRUE, mode = "0777")
dir.create(path = file.path(Cyclophosphamide.table),
           showWarnings = FALSE, recursive = TRUE, mode = "0777")
dir.create(Cyclophosphamide.output, recursive=TRUE, showWarnings=FALSE)
```

The dose response data for Cyclophosphamide is loaded.

```
load(Cyclophosphamide.data)
```

The results are investigated using the function **plotGrid** which plots the dose-response curves in panels with one cell line represented in each. The plot is shown in Figure 4.1. The bootstrapped dose-response curves are plotted along with the original curves.

```
plotGrid(DR.Cyclophosphamide, ylim = c(-1/12, 100), names = c(DLBCL, MM),
         drug = "Cyclophosphamide",
         bs.alpha = 50, bs.col=colours[c(1,2)],
         barcol="grey", line.col=colours[c(1,2)],
         ncol = 5)
```

For comparability the dose-response curves are plotted together and the variation of the summary statistics AUC_0 is illustrated by boxplots of the bootstrapped data in Figure 4.2.

```
par(mfrow = c(1,3), oma = c(1.2,0,0,0))

col.sc <- col.data$col[c(1,3,5)]
col = c(rep(col.sc[1], 4), rep(col.sc[2], 4), rep(col.sc[3], 4) )
lty = c(1:5,1:4, 1:5)
ylim <- c(-1/12, 100)

plot.DRdata(DR.Cyclophosphamide,  model = "G",
            ylim = ylim,
            col = col,
            main = "Cyclophosphamide (DLBCL lines lines)",
            lty = lty,
            drug = "Cyclophosphamide",
            n.columns = 1,
            legend.cex = 0.68,
            times = 48,
            names = names.DLBCL)

mtext("(a)", 3, line=1.5, adj=-0.145, cex=9/pointsize)
```

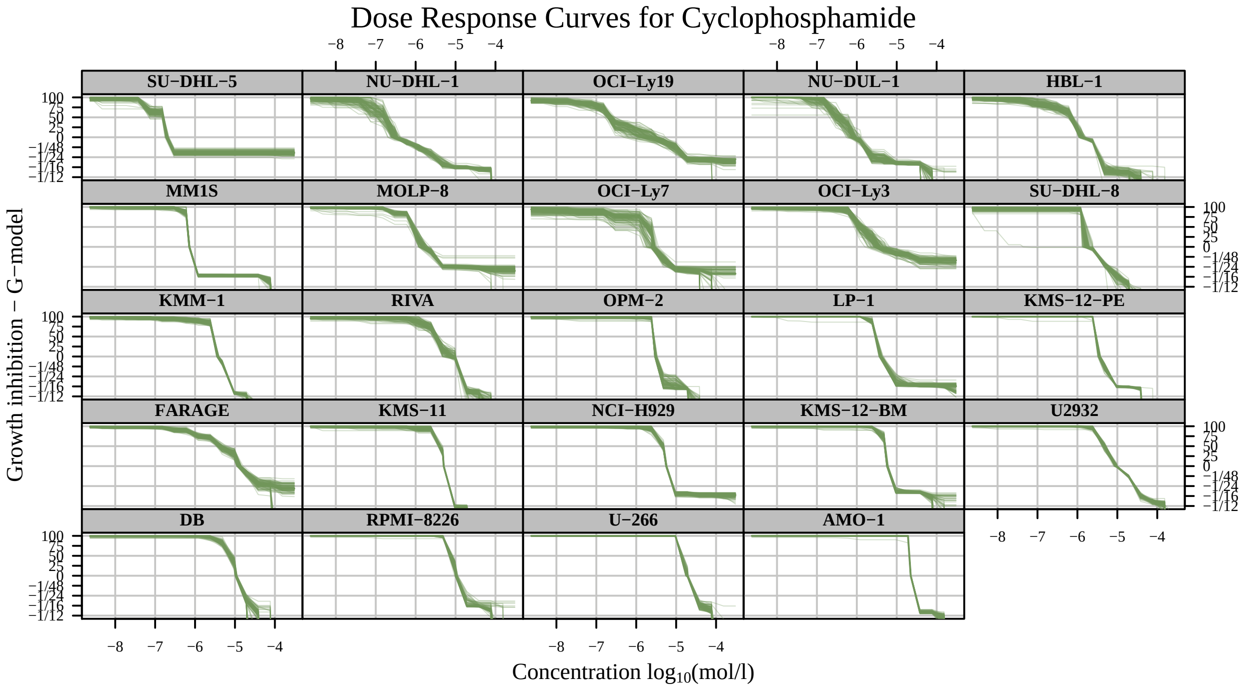

Figure 4.1: Plot of the dose response curves generated for the different cell lines.

```
col = c(rep(col.sc[1], 4), rep(col.sc[2], 4), rep(col.sc[3], 4) )
lty = c(1:4,1:4, 1:4)

plot.DRdata(DR.Cyclophosphamide,  model = "G",
            ylim = ylim,
            col = col,
            main = "Cyclophosphamide (MM lines)",
            lty = lty,
            drug = "Cyclophosphamide",
            n.columns = 1,
            legend.cex = 0.68,
            times = 48,
            names = names.MM)

mtext("(b)", 3, line=1.5, adj=-0.145, cex=9/pointsize)

col = c(rep(col.sc[1], 4), rep(col.sc[2], 4), rep(col.sc[3], 4),
        rep(col.sc[1], 4), rep(col.sc[2], 4), rep(col.sc[3], 4))

DRdataBoxplot(DR.Cyclophosphamide, type = "AUC", model = "G",
              col.all=col,
              splitvar = "Disease",
              drug = "Cyclophosphamide",
              time = 48,
              ylim = c(140,400),
              names = names)
mtext("(c)", 3, line=1.5, adj=-0.145, cex=9/pointsize)
```

The summary statistics shown in Table 4.1 are stored in the object `summary.Cyclophosphamide`. Table 4.1 is created by the following call to `pdfCI`:

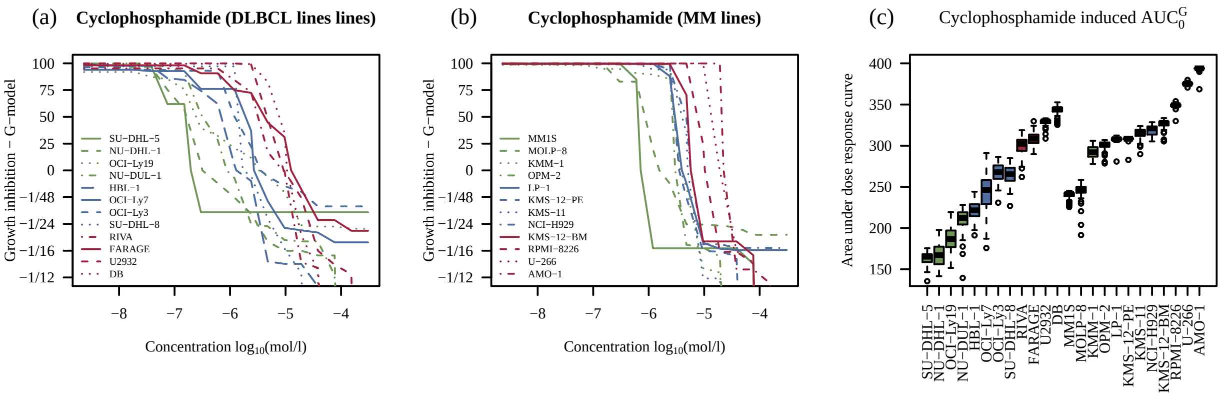

Figure 4.2: Plot of the dose response curves generated for the different cell lines. In panel A the dose response curves for the DLBCL cell lines are shown. Panel B shows the dose response curves for the MM cell lines. Panel C show boxplots of the AUC_0 summary statistic.

Table 4.1: Table created by the function `pdfCI`.

Cell Line	T0	Model G
	Hours	AUC_0
DB	39 (35;46)	346 (329;351)
FARAGE	33 (29;36)	311 (296;323)
HBL-1	50 (43;57)	226 (202;241)
NU-DHL-1	65 (54;89)	172 (142;191)
NU-DUL-1	41 (35;52)	214 (181;227)
OCI-Ly3	41 (33;51)	262 (247;285)
OCI-Ly7	76 (55;163)	258 (191;282)
OCI-Ly19	27 (25;38)	202 (161;214)
RIVA	35 (31;41)	296 (275;318)
SU-DHL-4		
SU-DHL-5	29 (24;36)	165 (148;174)
SU-DHL-8	234 (127;686)	271 (242;283)
U2932	45 (42;47)	330 (321;333)
MC-116		
KMM-1	39 (37;41)	295 (280;305)
KMS-11	49 (45;55)	318 (300;323)
KMS-12-BM	36 (30;41)	328 (316;332)
KMS-12-PE	37 (34;39)	309 (306;310)
LP-1	33 (31;37)	308 (304;311)
MM1S	40 (35;45)	242 (228;245)
MOLP-8	32 (27;38)	249 (225;256)
NCI-H929	26 (24;29)	322 (308;327)
OPM-2	35 (30;40)	303 (289;306)
RPMI-8226	33 (30;35)	350 (345;353)
U-266	63 (55;70)	376 (372;378)
AMO-1	44 (40;50)	395 (391;395)

```
summary.Cyclophosphamide <- CI(DR.Cyclophosphamide)$Cyclophosphamide
```

4.2 Establishment of REGS Predictor

The directory storing output for the signature based on linear regression is defined and created

```
Cyclophosphamide.output <- file.path("Output", "Cyclophosphamide", "Prediction/")
dir.create(Cyclophosphamide.output, recursive=TRUE, showWarnings=FALSE)
```

4.2.1 Cross Validation

```
drug <- "Cyclophosphamide"
file.Cyclophosphamide.predict <-
  file.path("GeneratedData/REGS", paste("Cyclophosphamide_prediction_genes.RData")) #entrez
```

```r
if(file.exists(file.Cyclophosphamide.predict)){
  load(file.Cyclophosphamide.predict)
}else{

  ## Create a matrix to store the results
  mat <- matrix(NaN, ncol = 4, nrow = length(alphas))
  colnames(mat) <- paste("Cyclophosphamide",
                         c("", ".lo", ".up", ".n"), sep = "")
  row.names(mat) <- alphas

  ## plot all the cross validation plots for later inspection
  pdf(file.path(Cyclophosphamide.output, "cvplots_prediction.pdf"))

  pred.Cyclophosphamide.fit <- list()

  int <- intersect(sampleNames(GEPCHO.sc), row.names(summary.Cyclophosphamide))

  # The GEP data is aligned with the dose response
  train.set <- exprs(GEPCHO.sc[,int])

  # The AUC0 values are extracted from the matrix
  y <- summary.Cyclophosphamide[int, "AUC", drop = FALSE]

  disease <- as.numeric(as.factor(as.character(metadataHBCCL[int, "Disease"]))) - 1

  train.set <- rbind(train.set, disease)

  # The cross validation is conducted for the supplied alpha values
  for(alpha in alphas){
    cat(alpha, "\n")
    fit <-
      cv.glmnet(x       = t(train.set[, rownames(y)]),
                y       = y[,1],
                standardize = FALSE,
                alpha   = alpha,
                nlambda = 600,
                nfolds  = ncol(train.set), # leave one out CV
                keep    = TRUE,
                grouped = FALSE,
                penalty.factor = c(rep(1, (nrow(train.set) - 1)), 0))

    ## plot the fit
    plot(fit, main = paste(drug, alpha))

    ## find the minimum and save upper and lower boundary
    wh.min <- which.min(fit$cvm)
    mat[paste(alpha), drug] <- min(fit$cvm)
    mat[paste(alpha), paste(drug, ".lo", sep = "")] <- fit$cvlo[wh.min]
    mat[paste(alpha), paste(drug, ".up", sep = "")] <- fit$cvup[wh.min]
    mat[paste(alpha), paste(drug, ".n",  sep = "")] <- fit$nzero[wh.min]

    ## add the cv fit to the list of fits
    pred.Cyclophosphamide.fit[[paste(alpha)]] <-fit
  }
  dev.off()
  save(pred.Cyclophosphamide.fit, mat, file = file.Cyclophosphamide.predict)
}
```

The best fit is found in the code chunck below:

```r
CyclophosphamidePredictor.cv <-
  pred.Cyclophosphamide.fit[[names(which.min(mat[paste(alphas), drug]))]]
```

The result of the cross validation is plotted in Figure 4.3. The code chunk below creates this plot.

```r
par(mfrow = c(1, 3))

plot.cv.alpha(x  = as.numeric(row.names(mat)),
              y  = mat[,drug],
              lo = mat[, paste(drug, ".lo", sep = "")],
              up = mat[, paste(drug, ".up", sep = "")],
              nzero = mat[,paste(drug, ".n", sep = "")],
              las   = 1,
              main1 = drug)

mtext("(a)", 3,
      line=1.5, adj=-0.145, cex=9/pointsize)

plot.cv.lambda(x  = log(CyclophosphamidePredictor.cv$lambda),
               y  = CyclophosphamidePredictor.cv$cvm,
               lo = CyclophosphamidePredictor.cv$cvlo,
               up = CyclophosphamidePredictor.cv$cvup,
               nzero = CyclophosphamidePredictor.cv$nzero,
               las   = 1,
               main1 = drug)

mtext("(b)", 3,
      line=1.5, adj=-0.145, cex=9/pointsize)

plot.coef(CyclophosphamidePredictor.cv,
          label.pct= 39,
          main = expression(paste("Regularisation Curves for Cyclophosphamide")))

mtext("(c)", 3,
      line=1.5, adj=-0.145, cex=9/pointsize)
```

For the α values resulting in the smallest cross validation error, the regularisation curves for the three drugs are plotted in the code chunk below. The plot is shown in Figure 4.3.
The minimum mean squared error for Cyclophosphamide was 2420.52, which was obtained at α =0.3 and $\log(\lambda)$ =1.69. The resulting GEP classifier for these parameters have non null coefficients for 27 genes.

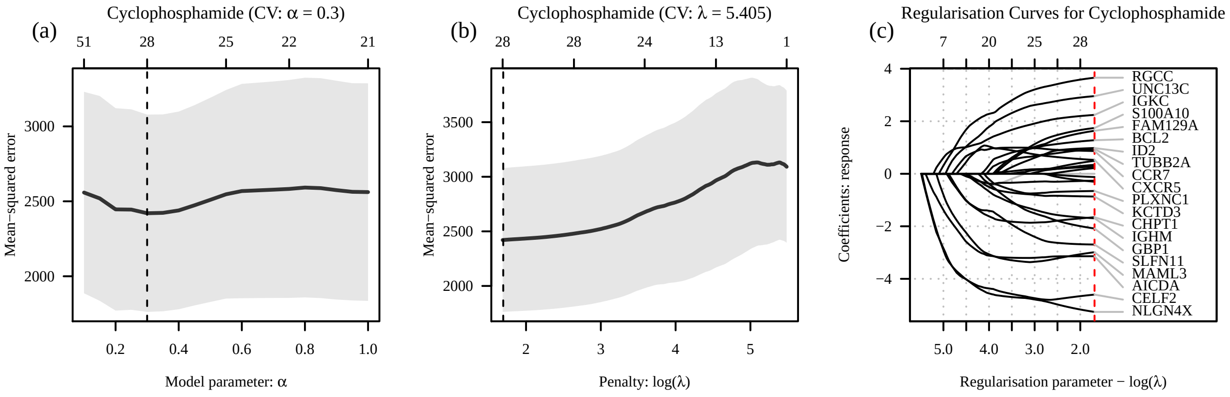

Figure 4.3: Cross validation and regularisation curves. In panel A the minimum mean squared error obtained for various α values are plotted. In panel B the mean-squared error is plotted against λ for the α value resulting in the minimum error and in Panel C the corresponding regularisation curves are shown. The genes for the 20 probe-sets associated with the largest coefficients are shown.

The REGS predictor for cyclophosphamide is established below.

```
COEF <- as.matrix(coef(CyclophosphamidePredictor.cv, s = "lambda.min"))

Cyclophosphamide.reisitance.coef <- COEF[COEF != 0, ]

probes <- names(Cyclophosphamide.reisitance.coef)[c(-1, -length(Cyclophosphamide.reisitance.coef))]

sd.Cyclophosphamide <- apply(exprs(GEPCHO.sc[probes, ]), 1, function(x) sd(x, na.rm = TRUE))

Cyclophosphamide.reisitance.pred.coef <-
  Cyclophosphamide.reisitance.coef[-length(Cyclophosphamide.reisitance.coef)] *
  c(1, sd.Cyclophosphamide)

CyclophosphamidePredictor<- function(newx, coef = Cyclophosphamide.reisitance.pred.coef){

  x <- rbind(1, newx[names(coef)[-1],,drop= FALSE])

  t(x) %*% as.matrix((coef))
}
```

The REGS predictor is saved for later use.

```
save(CyclophosphamidePredictor, Cyclophosphamide.reisitance.pred.coef,
     file = "GeneratedData/REGS/CyclophosphamidePredictor.RData")
```

4.3 Establishment of REGS Classifier

```
cutFun <-function(x) {
  cut(x,
      breaks=c(-Inf, quantile(x, c(1/3, 2/3), na.rm = TRUE), Inf),
      labels = c("Sensitive", "Intermediate","Resistant"))
}
```

4.3.1 Cross Validation

```
drug <- "Cyclophosphamide"
file.Cyclophosphamide.class <-
  file.path("GeneratedData/REGS",
            paste("Cyclophosphamide_classification_genes.RData"))

if(file.exists(file.Cyclophosphamide.class)){
  load(file.Cyclophosphamide.class)
}else{

  mat <- matrix(NaN, ncol = 4, nrow = length(alphas))
  colnames(mat) <- paste("Cyclophosphamide",
                         c("", ".lo", ".up", ".n"), sep = "")
  row.names(mat) <- alphas

  ## plot all the cross validation plots for later inspection
  pdf(file.path(Cyclophosphamide.output, "cvplots_classification.pdf"))

  class.Cyclophosphamide.fit <- list()
```

```r
# The GEP data is aligned with the dose response
train.set <- exprs(GEPCHO.sc)


R.data <- summary.Cyclophosphamide[colnames(train.set), ]
R.data$Disease <- as.character(metadataHBCCL[colnames(train.set), "Disease"])

R.data$AUC.class <-
  factor("Intermediate",
         levels= c("Sensitive", "Intermediate", "Resistant"))

R.data[R.data$Disease == "DLBCL", "AUC.class"] <-
  cutFun(R.data[R.data$Disease == "DLBCL", "AUC"])

R.data[R.data$Disease == "MM", "AUC.class"] <-
  cutFun(R.data[R.data$Disease == "MM", "AUC"])

R.data$AUC.class <- as.character(R.data$AUC.class)

y <- as.factor(R.data[R.data[, "AUC.class"] %in%
                        c("Resistant","Sensitive") ,"AUC.class"])

names(y) <- rownames(R.data[R.data[, "AUC.class"] %in%
                              c("Resistant","Sensitive") ,"AUC.class", drop = FALSE])

drug <- "Cyclophosphamide"
for(alpha in alphas){
  cat(drug, alpha, "\n")

  fit <-
    cv.glmnet(x = t(train.set[, names(y)]),
              alpha        = alpha,
              grouped      = FALSE,
              type.measure = "class",
              family       = "multinomial",
              nfolds       = length(y),
              keep         = TRUE,
              y            = y,
              lambda       = exp(seq(-6,3, length.out = 600)),
              standardize  = FALSE,
              nlambda      = 600)



  plot(fit, main = paste(drug, alpha))

  wh.min <- length(fit$cvm) - which.min(rev(fit$cvm))
  mat[paste(alpha), drug] <- min(fit$cvm)
  mat[paste(alpha), paste(drug, ".lo", sep = "")] <-
    fit$cvlo[wh.min]
  mat[paste(alpha), paste(drug, ".up", sep = "")] <-
    fit$cvup[wh.min]
  mat[paste(alpha), paste(drug, ".n", sep = "")] <-
    fit$nzero[wh.min]

  class.Cyclophosphamide.fit[[paste(alpha)]] <- fit

}
```

```
  dev.off()
  save(class.Cyclophosphamide.fit, mat, file = file.Cyclophosphamide.class)
}
```

The alpha value resulting in the smallest classication error is found in the code chunk below.

```
fit <- class.Cyclophosphamide.fit[[names(which.min((mat[, "Cyclophosphamide"])))]]
fit$lambda.min <-
  rev(fit$lambda)[which.min(rev(fit$cvm))]
CyclophosphamideClassifier.cv <- fit
```

The result of the cross validation is plotted in Figure 4.4. The code chunk below creates this plot.

```
par(mfrow = c(1,3))

plot.cv.alpha(x  = as.numeric(row.names(mat)),
              y  = mat[, drug],
              lo = mat[, paste(drug, ".lo", sep = "")],
              up = mat[, paste(drug, ".up", sep = "")],
              nzero = mat[,paste(drug, ".n", sep = "")],
              main1 = drug,
              las   = 1,
              ylab = "Misclassification error")

mtext("(a)", 3,
      line=1.5, adj=-0.145, cex=9/pointsize)

plot.cv.lambda(x  = log(CyclophosphamideClassifier.cv$lambda),
               y  = CyclophosphamideClassifier.cv$cvm,
               lo = CyclophosphamideClassifier.cv$cvlo,
               up = CyclophosphamideClassifier.cv$cvup,
               nzero = CyclophosphamideClassifier.cv$nzero,
               main1 = drug,
               las   = 1,
               ylab = "Misclassification error")

mtext("(b)", 3,
      line=1.5, adj=-0.145, cex=9/pointsize)

plot.coef(CyclophosphamideClassifier.cv,
          label.pct=32,
          main = paste("Regularisation curves for", drug))

mtext("(c)", 3,
      line=2, adj=-0.145, cex=9/pointsize)
```

The minimum classification error for Cyclophosphamide was 0.31 which was obtained at α =0.1 and $\log(\lambda)$ =−2.27. The resulting GEP classifier for these parameters have non null coefficients for 73 genes. For the α value resulting in the smallest cross validation error, the regularisation curves are plotted in Figure 4.4 Panel C.

The REGS classifier for cyclophosphamide is established below.

```
COEF <- as.matrix(coef(CyclophosphamideClassifier.cv, s = "lambda.min")$Resistant)

Cyclophosphamide.reisitance.coef <- COEF[COEF != 0, ]

probes <- names(Cyclophosphamide.reisitance.coef)[-1]

sd.Cyclophosphamide <- apply(exprs(GEPCHO.sc[probes, ]), 1, function(x) sd(x, na.rm = TRUE))
```

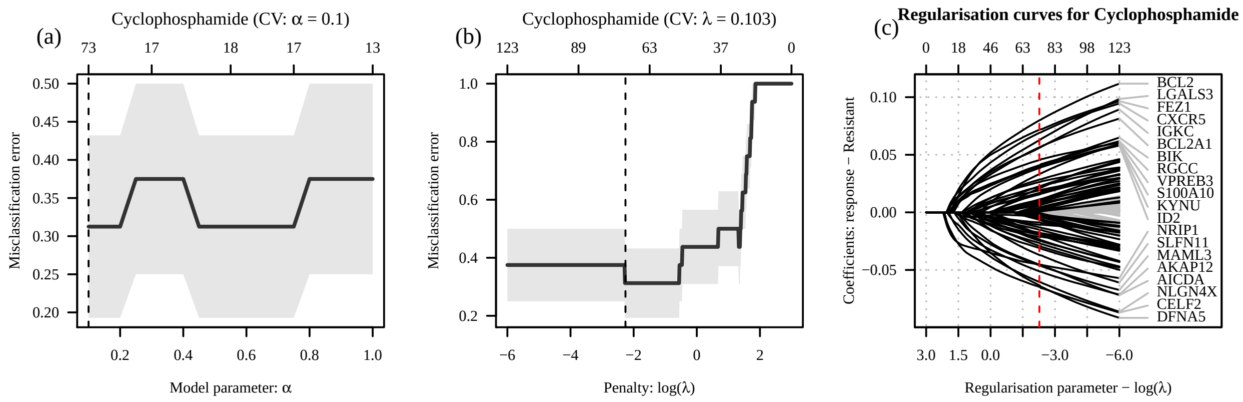

Figure 4.4: Cross validation and regularisation curves. In panel A the misclassification error obtained for various α values are plotted. In panel B the misclassification error is plotted against λ for the α value resulting in the minimum error and in Panel C the corresponding regularisation curves are shown. The genes for the 20 probe-sets associated with the largest coefficients are shown.

```
Cyclophosphamide.reisitance.class.coef <- Cyclophosphamide.reisitance.coef * c(1, sd.Cyclophosphamide)

CyclophosphamideClassifier <- function(newx, coef = Cyclophosphamide.reisitance.class.coef){

  x <- rbind(1, newx[names(coef)[-1],,drop= FALSE])

  prob <- t(x) %*% as.matrix((coef)  )

  prob <- exp(prob) / ( exp(prob) + exp(-prob))
  colnames(prob) <- "Resistant"
  prob
}
```

The REGS classifier is saved for later use.

```
save(CyclophosphamideClassifier, Cyclophosphamide.reisitance.class.coef,
     file = "GeneratedData/REGS/CyclophosphamideClassifier.RData")
```

5 Doxorubicin

5.1 Load Doxorubicin Dose Response Data

Set seed to ensure reprodicible research.

```
set.seed(1000)
```

Determine directories for output.

```
Doxorubicin.figure  <- file.path("Output", "Doxorubicin", "Figures/")
Doxorubicin.table   <- file.path("Output", "Doxorubicin", "Tables/")
Doxorubicin.output  <- file.path("Output", "Doxorubicin", "Prediction/")
```

The directories storing R-output are created.

```
dir.create(path = file.path(Doxorubicin.figure),
           showWarnings = FALSE, recursive = TRUE, mode = "0777")
dir.create(path = file.path(Doxorubicin.table),
           showWarnings = FALSE, recursive = TRUE, mode = "0777")
dir.create(Doxorubicin.output, recursive=TRUE, showWarnings=FALSE)
```

The dose response data for doxorubicin is loaded.

```
load(Doxorubicin.data)
```

The results are investigated using the function `plotGrid` which plots the dose-response curves in panels with one cell line represented in each. The plot is shown in Figure 5.1. The bootstrapped dose-response curves are plotted along with the original curves.

```
plotGrid(DR.Doxorubicin, ylim = c(-1/12, 100), names = c(DLBCL, MM),
         bs.alpha = 50, bs.col=colours[c(1,2)],
         barcol="grey", line.col=colours[c(1,2)],
         ncol = 5)
```

For comparability the dose-response curves are plotted together and the variation of the summary statistics AUC_0 is illustrated by boxplots of the bootstrapped data in Figure 5.2.

```
par(mfrow = c(1,3), oma = c(1.2,0,0,0))

col.sc <- col.data$col[c(1,3,5)]
col = c(rep(col.sc[1], 5), rep(col.sc[2], 4), rep(col.sc[3], 5) )
lty = c(1:5,1:4, 1:5)
ylim <- c(-1/12, 100)

plot.DRdata(DR.Doxorubicin,  model = "G",
            ylim = ylim,
            col = col,
            main = "Doxorubicin (DLBCL lines lines)",
            lty = lty,
           # drug = "Doxorubicin",
            n.columns = 1,
            legend.cex = 0.68,
            times = 48,
            names = names.DLBCL)

mtext("(a)", 3, line=1.5, adj=-0.145, cex=9/pointsize)
```

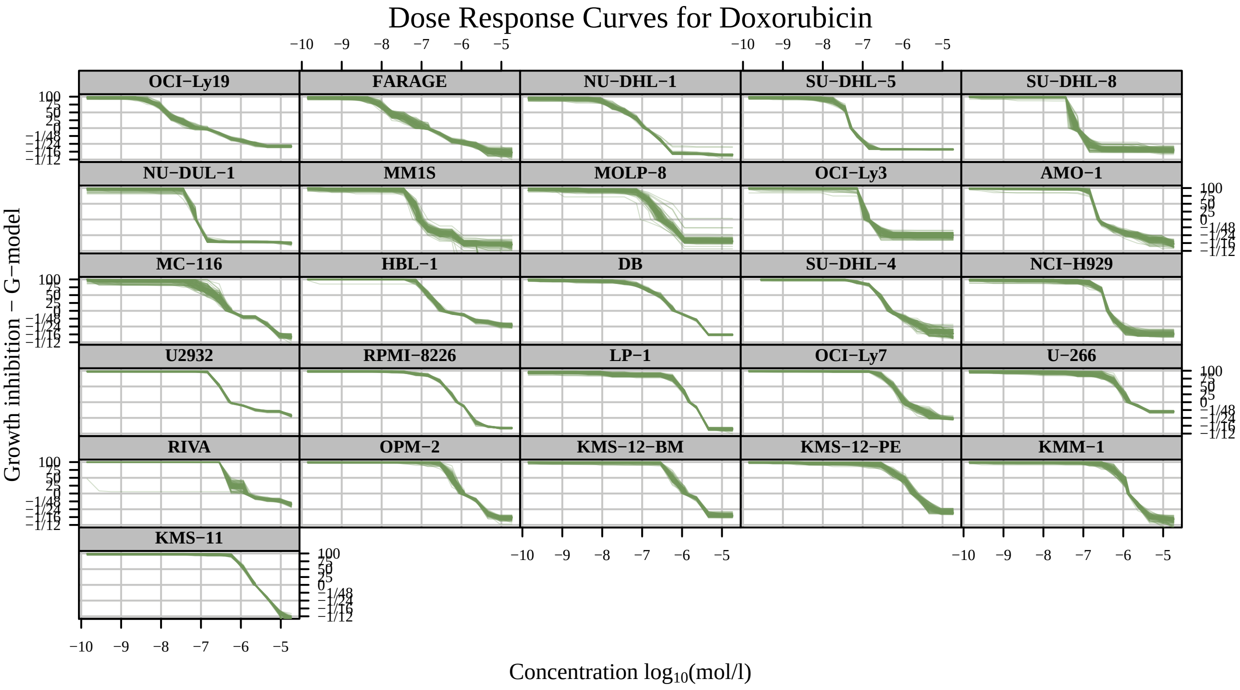

Figure 5.1: Plot of the dose response curves generated for the different cell lines.

```
col = c(rep(col.sc[1], 4), rep(col.sc[2], 4), rep(col.sc[3], 4) )
lty = c(1:4,1:4, 1:4)

plot.DRdata(DR.Doxorubicin,  model = "G",
            ylim = ylim,
            col = col,
            main = "Doxorubicin (MM lines)",
            lty = lty,
            drug = "Doxorubicin",
            n.columns = 1,
            legend.cex = 0.68,
            times = 48,
            names = names.MM)

mtext("(b)", 3, line=1.5, adj=-0.145, cex=9/pointsize)

col = c(rep(col.sc[1], 5), rep(col.sc[2], 4), rep(col.sc[3], 5),
        rep(col.sc[1], 4), rep(col.sc[2], 4), rep(col.sc[3], 4))

DRdataBoxplot(DR.Doxorubicin, type = "AUC", model = "G",
              col.all=col,
              splitvar = "Disease",
              drug = "Doxorubicin",
              time = 48,
              ylim = c(150,360),
              names = names)
mtext("(c)", 3, line=1.5, adj=-0.145, cex=9/pointsize)
```

The summary statistics shown in Table 5.1 are stored in the object `summary.Doxorubicin`. Table 5.1 is created by the following call to `pdfCI`:

```
summary.Doxorubicin <- CI(DR.Doxorubicin)$Doxorubicin
```

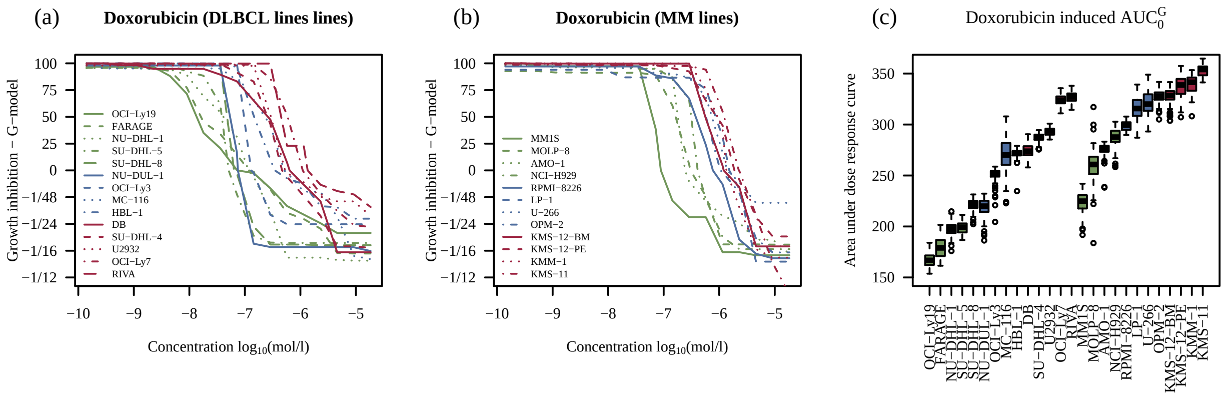

Figure 5.2: Plot of the dose response curves generated for the different cell lines. In panel A the dose response curves for the DLBCL cell lines are shown. Panel B shows the dose response curves for the MM cell lines. Panel C show boxplots of the AUC_0 summary statistic.

Table 5.1: Table created by the function `pdfCI`.

Cell Line	T0	Model G
	Hours	AUC_0
DB	37 (34;39)	276 (261;286)
FARAGE	58 (49;71)	180 (164;198)
HBL-1	46 (42;49)	272 (264;277)
NU-DHL-1	29 (26;32)	201 (184;212)
NU-DUL-1	48 (39;63)	224 (192;229)
OCI-Ly3	91 (70;138)	253 (229;258)
OCI-Ly7	24 (21;26)	325 (312;334)
OCI-Ly19	39 (37;43)	167 (157;179)
RIVA	68 (54;93)	327 (316;337)
SU-DHL-4	36 (34;39)	289 (278;293)
SU-DHL-5	32 (29;35)	202 (188;210)
SU-DHL-8	85 (54;223)	222 (208;230)
U2932	34 (33;36)	295 (286;299)
MC-116	50 (42;69)	272 (235;301)
KMM-1	44 (39;53)	346 (323;352)
KMS-11	48 (46;51)	356 (343;361)
KMS-12-BM	47 (41;55)	331 (308;338)
KMS-12-PE	24 (20;28)	340 (315;352)
LP-1	33 (30;35)	316 (292;335)
MM1S	37 (25;59)	228 (198;238)
MOLP-8	34 (18;56)	253 (226;289)
NCI-H929	28 (26;31)	291 (264;300)
OPM-2	57 (47;75)	329 (314;337)
RPMI-8226	30 (29;32)	297 (291;306)
U-266	48 (43;56)	325 (299;341)
AMO-1	32 (30;35)	269 (262;282)

5.2 Establishment of REGS Predictor

The directory storing output for the signature based on linear regression is defined and created

```r
Doxorubicin.output <- file.path("Output", "Doxorubicin", "Prediction/")
dir.create(Doxorubicin.output, recursive=TRUE, showWarnings=FALSE)
```

5.2.1 Cross Validation

```r
drug <- "Doxorubicin"
file.Doxorubicin.predict <-
  file.path("GeneratedData/REGS", paste("Doxorubicin_prediction_genes.RData")) #entrez

if(file.exists(file.Doxorubicin.predict)){
  load(file.Doxorubicin.predict)
}else{
```

```r
  ## Create a matrix to store the results
  mat <- matrix(NaN, ncol = 4, nrow = length(alphas))
  colnames(mat) <- paste("Doxorubicin",
                         c("", ".lo", ".up", ".n"), sep = "")
  row.names(mat) <- alphas

  ## plot all the cross validation plots for later inspection
  pdf(file.path(Doxorubicin.output, "cvplots_prediction.pdf"))

  pred.Doxorubicin.fit <- list()

  int <- intersect(sampleNames(GEPCHO.sc), row.names(summary.Doxorubicin))

  ## The GEP data is aligned with the dose response
  train.set <- exprs(GEPCHO.sc[,int])

  ## The AUC0 values are extracted from the matrix
  y <- summary.Doxorubicin[int, "AUC", drop = FALSE]

  disease <- as.numeric(as.factor(as.character(metadataHBCCL[int, "Disease"]))) - 1

  train.set <- rbind(train.set, disease)

  # The cross validation is conducted for the supplied alpha values
  for(alpha in alphas){
    cat(alpha, "\n")
    fit <-
      cv.glmnet(x        = t(train.set[, rownames(y)]),
                y        = y[,1],
                standardize = FALSE,
                alpha    = alpha,
                nlambda = 600,
                nfolds   = ncol(train.set), # leave one out CV
                keep     = TRUE,
                grouped = FALSE,
                penalty.factor = c(rep(1, (nrow(train.set) - 1)), 0))

    ## plot the fit
    plot(fit, main = paste(drug, alpha))

    ## find the minimum and save upper and lower boundary
    wh.min <- which.min(fit$cvm)
    mat[paste(alpha), drug] <- min(fit$cvm)
    mat[paste(alpha), paste(drug, ".lo", sep = "")] <- fit$cvlo[wh.min]
    mat[paste(alpha), paste(drug, ".up", sep = "")] <- fit$cvup[wh.min]
    mat[paste(alpha), paste(drug, ".n",  sep = "")] <- fit$nzero[wh.min]

    ## add the cv fit to the list of fits
    pred.Doxorubicin.fit[[paste(alpha)]] <-fit
  }
  dev.off()
  save(pred.Doxorubicin.fit, mat, file = file.Doxorubicin.predict)
}
```

The best fit is found in the code chunck below:

```r
DoxorubicinPredictor.cv <-
      pred.Doxorubicin.fit[[names(which.min(mat[paste(alphas), drug]))]]
```

The result of the cross validation is plotted in Figure 5.3. The code chunk below creates this plot.
The minimum mean squared for Doxorubicin was 2082.78, which was obtained at α =0.1 and $\log(\lambda)$ =2.51. The resulting GEP classifier for these parameters have non null coefficients for 52 genes.

```
par(mfrow = c(1, 3))

plot.cv.alpha(x  = as.numeric(row.names(mat)),
              y  = mat[,drug],
              lo = mat[, paste(drug, ".lo", sep = "")],
              up = mat[, paste(drug, ".up", sep = "")],
              nzero = mat[,paste(drug, ".n", sep = "")],
              las   = 1,
              main1 = drug)

mtext("(a)", 3,
      line=1.5, adj=-0.145, cex=9/pointsize)

plot.cv.lambda(x  = log(DoxorubicinPredictor.cv$lambda),
               y  = DoxorubicinPredictor.cv$cvm,
               lo = DoxorubicinPredictor.cv$cvlo,
               up = DoxorubicinPredictor.cv$cvup,
               nzero = DoxorubicinPredictor.cv$nzero,
               las   = 1,
               main1 = drug)

mtext("(b)", 3,
      line=1.5, adj=-0.145, cex=9/pointsize)

plot.coef(DoxorubicinPredictor.cv,
          label.pct= 41,
          main = expression(paste("Regularisation Curves for Doxorubicin")))

mtext("(c)", 3,
      line=1.5, adj=-0.145, cex=9/pointsize)
```

For the α values resulting in the smallest cross validation error, the regularisation curves for the three drugs are plotted in the code chunk below. The plot is shown in Figure 5.3.

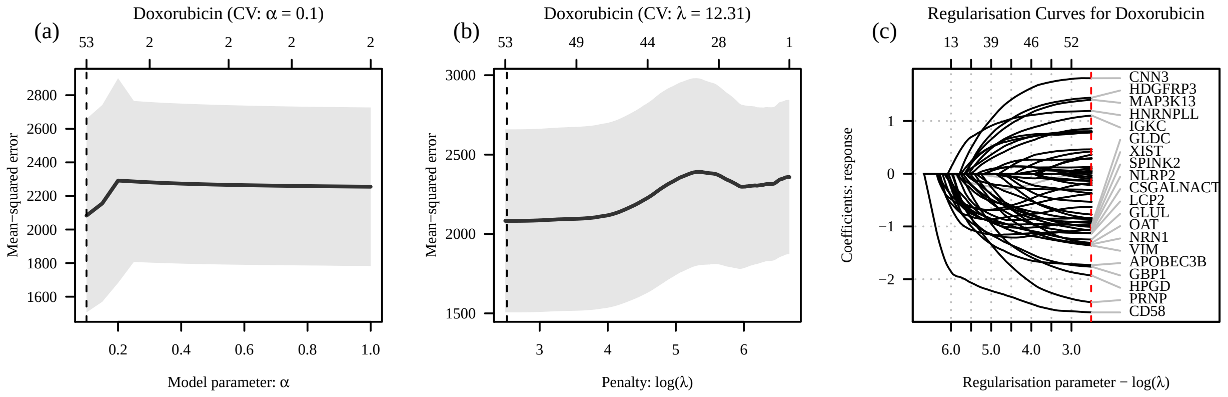

Figure 5.3: Cross validation and regularisation curves. In panel A the minimum mean squared error obtained for various α values are plotted. In panel B the mean-squared error is plotted against λ for the α value resulting in the minimum error and in Panel C the corresponding regularisation curves are shown. The genes for the 20 probe-sets associated with the largest coefficients are shown.

The REGS predictor for doxorubicin is established below.

```
COEF <- as.matrix(coef(DoxorubicinPredictor.cv, s = "lambda.min"))

Doxorubicin.reisitance.coef <- COEF[COEF != 0, ]

probes <- names(Doxorubicin.reisitance.coef)[c(-1, -length(Doxorubicin.reisitance.coef))]

sd.Doxorubicin <- apply(exprs(GEPCHO.sc[probes, ]), 1, function(x) sd(x, na.rm = TRUE))

Doxorubicin.reisitance.pred.coef <-
  Doxorubicin.reisitance.coef[-length(Doxorubicin.reisitance.coef)] *
  c(1, sd.Doxorubicin)

DoxorubicinPredictor<- function(newx, coef = Doxorubicin.reisitance.pred.coef){

  x <- rbind(1, newx[names(coef)[-1],,drop= FALSE])

  t(x) %*% as.matrix((coef))
}
```

The REGS predictor is saved for later use.

```
save(DoxorubicinPredictor, Doxorubicin.reisitance.pred.coef,
     file = "GeneratedData/REGS/DoxorubicinPredictor.RData")
```

5.3 Establishment of REGS Classifier

```
cutFun <-function(x) {
  cut(x,
      breaks=c(-Inf, quantile(x, c(1/3, 2/3),na.rm = TRUE), Inf),
      labels = c("Sensitive", "Intermediate","Resistant"))
}
```

5.3.1 Cross Validation

```
file.Doxorubicin.class <-
  file.path("GeneratedData/REGS",
            paste("Doxorubicin_classification_genes.RData"))

if(file.exists(file.Doxorubicin.class)){
  load(file.Doxorubicin.class)
}else{

  mat <- matrix(NaN, ncol = 4, nrow = length(alphas))
  colnames(mat) <- paste("Doxorubicin",
                         c("", ".lo", ".up", ".n"), sep = "")
  row.names(mat) <- alphas

  ## plot all the cross validation plots for later inspection
  pdf(file.path(Doxorubicin.output, "cvplots_classification.pdf"))

  class.Doxorubicin.fit <- list()


  # The GEP data is aligned with the dose response
  train.set <- exprs(GEPCHO.sc)
```

```r
R.data <- summary.Doxorubicin[colnames(train.set), ]
R.data$Disease <- as.character(metadataHBCCL[colnames(train.set), "Disease"])

R.data$AUC.class <-
  factor("Intermediate",
         levels= c("Sensitive", "Intermediate", "Resistant"))

R.data[R.data$Disease == "DLBCL", "AUC.class"] <-
  cutFun(R.data[R.data$Disease == "DLBCL", "AUC"])

R.data[R.data$Disease == "MM", "AUC.class"] <-
  cutFun(R.data[R.data$Disease == "MM", "AUC"])

R.data$AUC.class <- as.character(R.data$AUC.class)

y <- as.factor(R.data[R.data[, "AUC.class"] %in%
                        c("Resistant","Sensitive") ,"AUC.class"])

names(y) <- rownames(R.data[R.data[, "AUC.class"] %in%
                              c("Resistant","Sensitive") ,"AUC.class", drop = FALSE])

y <- as.factor(doseresponse.cat[doseresponse.cat[,drug] %in%
                                  c("Resistant","Sensitive") ,drug])


drug <- "Doxorubicin"
for(alpha in alphas){
  cat(drug, alpha, "\n")

  fit <-
    cv.glmnet(x = t(train.set[, names(y)]),
              alpha        = alpha,
              grouped      = FALSE,
              type.measure = "class",
              family       = "multinomial",
              nfolds       = length(y),
              keep         = TRUE,
              y            = y,
              lambda       = rev(exp(seq(-6, 3, length.out = 600))),
              standardize  = FALSE,
              nlambda      = 600)



  plot(fit, main = paste(drug, alpha))

  wh.min <- length(fit$cvm) - which.min(rev(fit$cvm))
  mat[paste(alpha), drug] <- min(fit$cvm)
  mat[paste(alpha), paste(drug, ".lo", sep = "")] <-
    fit$cvlo[wh.min]
  mat[paste(alpha), paste(drug, ".up", sep = "")] <-
    fit$cvup[wh.min]
  mat[paste(alpha), paste(drug, ".n", sep = "")] <-
    fit$nzero[wh.min]

  class.Doxorubicin.fit[[paste(alpha)]] <- fit
```

```
  }

  dev.off()
  save(class.Doxorubicin.fit, mat, file = file.Doxorubicin.class)
}
```

The alpha value resulting in the smallest classication error is found in the code chunk below.

```
fit <- class.Doxorubicin.fit[[names(which.min((mat[, "Doxorubicin"])))]]
fit$lambda.min <-
  rev(fit$lambda)[which.min(rev(fit$cvm))]
DoxorubicinClassifier.cv <- fit
```

The result of the cross validation is plotted in Figure 5.4. The code chunk below creates this plot.

```
par(mfrow = c(1,3))
drug <- "Doxorubicin"
plot.cv.alpha(x  = as.numeric(row.names(mat)),
              y  = mat[, drug],
              lo = mat[, paste(drug, ".lo", sep = "")],
              up = mat[, paste(drug, ".up", sep = "")],
              nzero = mat[,paste(drug, ".n", sep = "")],
              main1 = drug,
              las   = 1,
              ylab = "Misclassification error")

mtext("(a)", 3,
      line=1.5, adj=-0.145, cex=9/pointsize)

plot.cv.lambda(x  = log(DoxorubicinClassifier.cv$lambda),
               y  = DoxorubicinClassifier.cv$cvm,
               lo = DoxorubicinClassifier.cv$cvlo,
               up = DoxorubicinClassifier.cv$cvup,
               nzero = DoxorubicinClassifier.cv$nzero,
               main1 = drug,
               las   = 1,
               ylab = "Misclassification error")

mtext("(b)", 3,
      line=1.5, adj=-0.145, cex=9/pointsize)
plot.coef(DoxorubicinClassifier.cv,
          label.pct=38,
          main = paste("Regularisation curves for", drug))

mtext("(c)", 3,
      line=2, adj=-0.145, cex=9/pointsize)
```

The minimum classification error for Doxorubicin was 0.11 which was obtained at α =0.1 and $\log(\lambda)$ =−6. The resulting GEP classifier for these parameters have non null coefficients for 118 genes. For the α value resulting in the smallest cross validation error, the regularisation curves are plotted in Figure 5.4 Panel C.

The REGS classifier for doxorubicin is established below.

```
COEF <- as.matrix(coef(DoxorubicinClassifier.cv, s = "lambda.min")$Resistant)

Doxorubicin.reisitance.coef <- COEF[COEF != 0, ]

probes <- names(Doxorubicin.reisitance.coef)[-1]
```

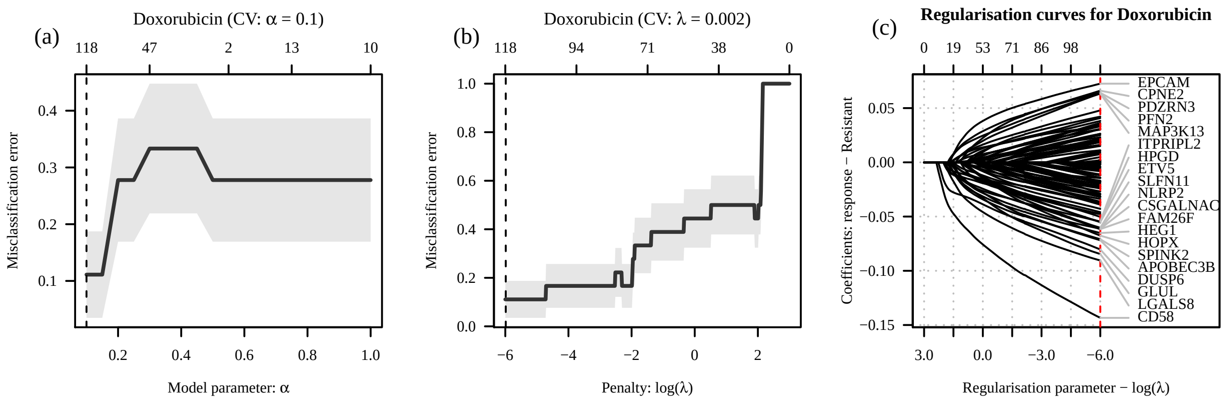

Figure 5.4: Cross validation and regularisation curves. In panel A the misclassification error obtained for various α values are plotted. In panel B the misclassification error is plotted against λ for the α value resulting in the minimum error and in Panel C the corresponding regularisation curves are shown. The genes for the 20 probe-sets associated with the largest coefficients are shown.

```
sd.Doxorubicin <- apply(exprs(GEPCHO.sc[probes, ]), 1, function(x) sd(x, na.rm = TRUE))

Doxorubicin.reisitance.class.coef <- Doxorubicin.reisitance.coef * c(1, sd.Doxorubicin)

DoxorubicinClassifier <- function(newx, coef = Doxorubicin.reisitance.class.coef){

  x <- rbind(1, newx[names(coef)[-1],,drop= FALSE])

  prob <- t(x) %*% as.matrix((coef)  )

  prob <- exp(prob) / ( exp(prob) + exp(-prob))
  colnames(prob) <- "Resistant"
  prob
}
```

The REGS classifier is saved for later use.

```
save(DoxorubicinClassifier, Doxorubicin.reisitance.class.coef,
     file = "GeneratedData/REGS/DoxorubicinClassifier.RData")
```

6 Vincristine

6.1 Load Vincristine Dose Response Data

Set seed to ensure reprodicible research.

```
set.seed(1000)
```

Determine directories for output.

```
Vincristine.figure  <- file.path("Output", "Vincristine", "Figures/")
Vincristine.table   <- file.path("Output", "Vincristine", "Tables/")
Vincristine.output <- file.path("Output", "Vincristine", "Prediction/")
```

The directories storing R-output are created.

```
dir.create(path = file.path(Vincristine.figure),
           showWarnings = FALSE, recursive = TRUE, mode = "0777")
dir.create(path = file.path(Vincristine.table),
           showWarnings = FALSE, recursive = TRUE, mode = "0777")
dir.create(Vincristine.output, recursive=TRUE, showWarnings=FALSE)
```

The dose response data for Vincristine is loaded.

```
load(Vincristine.data)
```

The results are investigated using the function **plotGrid** which plots the dose-response curves in panels with one cell line represented in each. The plot is shown in Figure 6.1. The bootstrapped dose-response curves are plotted along with the original curves.

```
plotGrid(DR.Vincristine, ylim = c(-1/12, 100), names = c(DLBCL, MM),
         bs.alpha = 50, bs.col=colours[c(1,2)],
         barcol="grey", line.col=colours[c(1,2)],
         ncol = 5)
```

For comparability the dose-response curves are plotted together and the variation of the summary statistics AUC_0 is illustrated by boxplots of the bootstrapped data in Figure 6.2.

```
par(mfrow = c(1,3), oma = c(1.2,0,0,0))

col.sc <- col.data$col[c(1,3,5)]
col = c(rep(col.sc[1], 4), rep(col.sc[2], 4), rep(col.sc[3], 4) )
lty = c(1:5,1:4, 1:5)
ylim <- c(-1/12, 100)

plot.DRdata(DR.Vincristine,  model = "G",
            ylim = ylim,
            col = col,
            main = "Vincristine (DLBCL lines lines)",
            lty = lty,
           # drug = "Vincristine",
            n.columns = 2,
            legend.place = "topright",
            legend.cex = 0.68,
            times = 48,
            names = names.DLBCL)

mtext("(a)", 3, line=1.5, adj=-0.145, cex=9/pointsize)
```

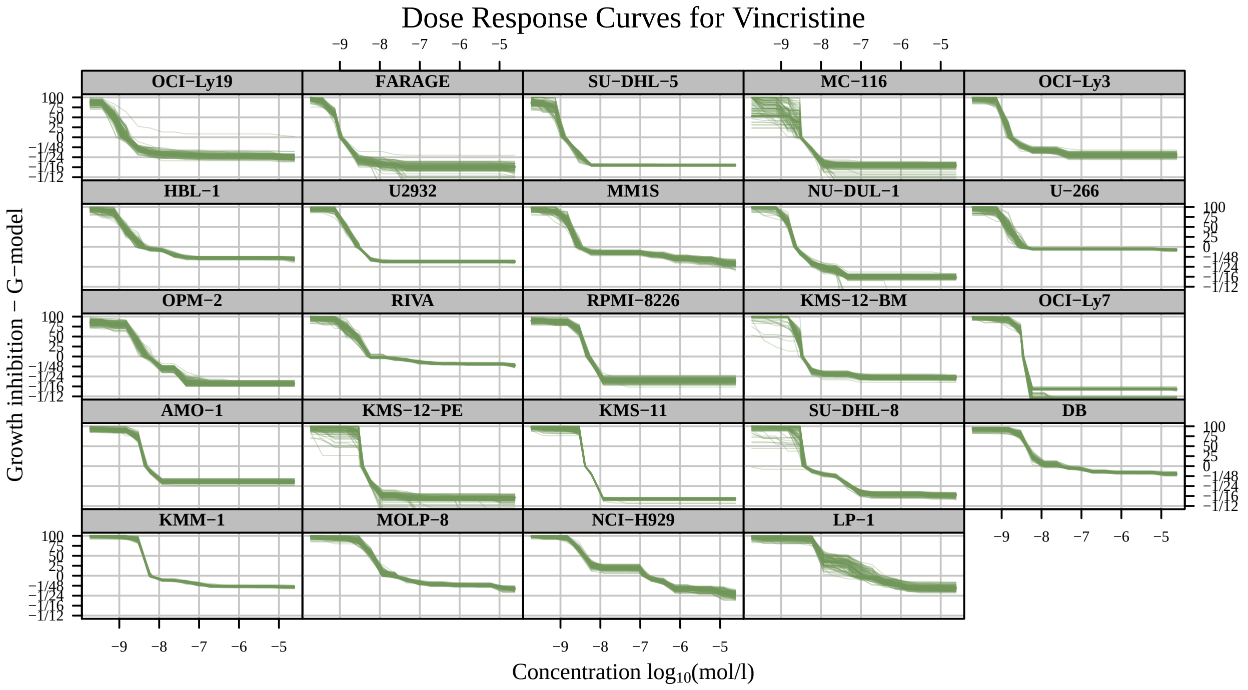

Figure 6.1: Plot of the dose response curves generated for the different cell lines.

```
col = c(rep(col.sc[1], 4), rep(col.sc[2], 4), rep(col.sc[3], 4) )
lty = c(1:4,1:4, 1:4)

plot.DRdata(DR.Vincristine,  model = "G",
            ylim = ylim,
            col = col,
            main = "Vincristine (MM lines)",
            lty = lty,
            drug = "Vincristine",
            n.columns = 2,
            legend.place = "topright",
            legend.cex = 0.68,
            times = 48,
            names = names.MM)

mtext("(b)", 3, line=1.5, adj=-0.145, cex=9/pointsize)

col = c(rep(col.sc[1], 5), rep(col.sc[2], 4), rep(col.sc[3], 5),
        rep(col.sc[1], 4), rep(col.sc[2], 4), rep(col.sc[3], 4))

DRdataBoxplot(DR.Vincristine, type = "AUC", model = "G",
              col.all=col,
              splitvar = "Disease",
              drug = "Vincristine",
              time = 48,
              ylim = c(25,225),
              names = names)
mtext("(c)", 3, line=1.5, adj=-0.145, cex=9/pointsize)
```

The summary statistics shown in Table 6.1 are stored in the object `summary.Vincristine`. Table 6.1 is created by the following call to `pdfCI`:

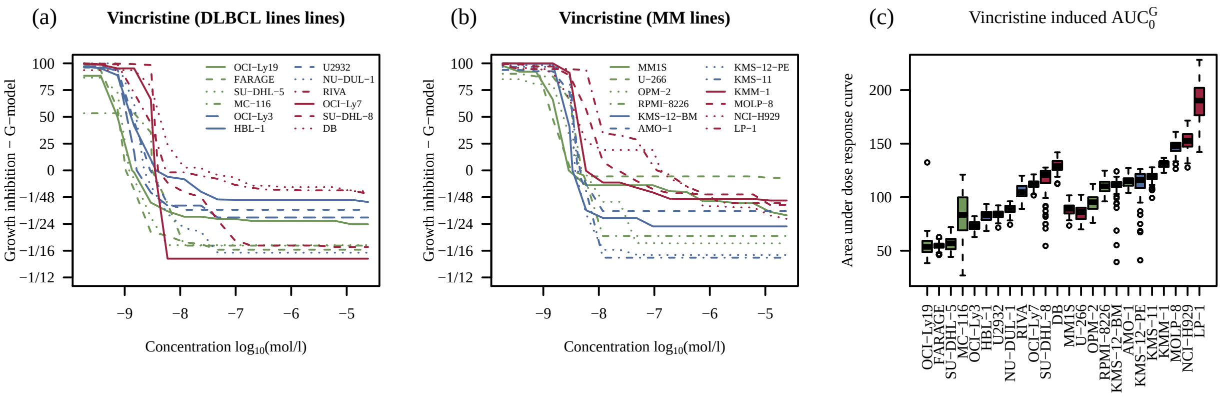

Figure 6.2: Plot of the dose response curves generated for the different cell lines. In panel A the dose response curves for the DLBCL cell lines are shown. Panel B shows the dose response curves for the MM cell lines. Panel C show boxplots of the AUC_0 summary statistic.

Table 6.1: Table created by the function `pdfCI`.

Cell Line	T0	Model G
	Hours	AUC_0
DB	34 (31;38)	131 (119;139)
FARAGE	38 (34;47)	56 (47;60)
HBL-1	46 (39;54)	85 (71;91)
NU-DHL-1		
NU-DUL-1	34 (30;40)	90 (79;95)
OCI-Ly3	55 (44;66)	75 (65;81)
OCI-Ly7	29 (27;33)	114 (103;119)
OCI-Ly19	44 (29;62)	54 (40;68)
RIVA	37 (31;43)	109 (91;117)
SU-DHL-4		
SU-DHL-5	45 (38;59)	58 (46;71)
SU-DHL-8	138 (94;255)	126 (73;127)
U2932	39 (35;44)	85 (77;91)
MC-116	168 (105;567)	62 (33;118)
KMM-1	35 (32;37)	133 (125;135)
KMS-11	61 (55;68)	123 (107;127)
KMS-12-BM	59 (48;76)	112 (79;118)
KMS-12-PE	51 (40;79)	121 (72;126)
LP-1	40 (27;58)	187 (151;219)
MM1S	59 (51;72)	90 (79;99)
MOLP-8	34 (30;38)	149 (132;158)
NCI-H929	33 (31;36)	149 (137;168)
OPM-2	47 (41;54)	95 (80;110)
RPMI-8226	29 (24;34)	111 (98;120)
U-266	53 (43;66)	90 (73;101)
AMO-1	41 (36;47)	116 (105;125)

```
summary.Vincristine <- CI(DR.Vincristine)$Vincristine
```

6.2 Establishment of REGS Predictor

The directory storing output for the signature based on linear regression is defined and created

```
Vincristine.output <- file.path("Output", "Vincristine", "Prediction/")
dir.create(Vincristine.output, recursive=TRUE, showWarnings=FALSE)
```

6.2.1 Cross Validation

```
drug <- "Vincristine"
file.Vincristine.predict <-
  file.path("GeneratedData/REGS", paste("Vincristine_prediction_genes.RData")) #entrez
```

```r
if(file.exists(file.Vincristine.predict)){
  load(file.Vincristine.predict)
}else{

  ## Create a matrix to store the results
  mat <- matrix(NaN, ncol = 4, nrow = length(alphas))
  colnames(mat) <- paste("Vincristine",
                         c("", ".lo", ".up", ".n"), sep = "")
  row.names(mat) <- alphas

  ## plot all the cross validation plots for later inspection
  pdf(file.path(Vincristine.output, "cvplots_prediction.pdf"))

  pred.Vincristine.fit <- list()

  int <- intersect(sampleNames(GEPCHO.sc), row.names(summary.Vincristine))

  # The GEP data is aligned with the dose response
  train.set <- exprs(GEPCHO.sc[, int])

  # The AUC0 values are extracted from the matrix
  y <- summary.Vincristine[int, "AUC", drop = FALSE]

  disease <- as.numeric(as.factor(as.character(metadataHBCCL[int, "Disease"]))) - 1

  train.set <- rbind(train.set, disease)

  # The cross validation is conducted for the supplied alpha values
  for(alpha in alphas){
    cat(alpha, "\n")
    fit <-
      cv.glmnet(x       = t(train.set[, rownames(y)]),
                y       = y[,1],
                standardize = FALSE,
                alpha   = alpha,
                nlambda = 600,
                nfolds  = ncol(train.set), # leave one out CV
                keep    = TRUE,
                grouped = FALSE,
                penalty.factor = c(rep(1, (nrow(train.set) - 1)), 0))

    ## plot the fit

    plot(fit, main = paste(drug, alpha))

    ## find the minimum and save upper and lower boundary
    wh.min <- which.min(fit$cvm)
    mat[paste(alpha), drug] <- min(fit$cvm)
    mat[paste(alpha), paste(drug, ".lo", sep = "")] <- fit$cvlo[wh.min]
    mat[paste(alpha), paste(drug, ".up", sep = "")] <- fit$cvup[wh.min]
    mat[paste(alpha), paste(drug, ".n",  sep = "")] <- fit$nzero[wh.min]

    ## add the cv fit to the list of fits
    pred.Vincristine.fit[[paste(alpha)]] <-fit
  }
  dev.off()
  save(pred.Vincristine.fit, mat, file = file.Vincristine.predict)
}
```

The best fit is found in the code chunck below:

```
VincristinePredictor.cv <-
      pred.Vincristine.fit[[names(which.min(mat[paste(alphas), drug]))]]
```

The result of the cross validation is plotted in Figure 6.3. The code chunk below creates this plot.

```
par(mfrow = c(1, 3))

plot.cv.alpha(x  = as.numeric(row.names(mat)),
              y  = mat[,drug],
              lo = mat[, paste(drug, ".lo", sep = "")],
              up = mat[, paste(drug, ".up", sep = "")],
              nzero = mat[,paste(drug, ".n", sep = "")],
              las   = 1,
              main1 = drug)

mtext("(a)", 3,
      line=1.5, adj=-0.145, cex=9/pointsize)

plot.cv.lambda(x  = log(VincristinePredictor.cv$lambda),
               y  = VincristinePredictor.cv$cvm,
               lo = VincristinePredictor.cv$cvlo,
               up = VincristinePredictor.cv$cvup,
               nzero = VincristinePredictor.cv$nzero,
               las   = 1,
               main1 = drug)

mtext("(b)", 3,
      line=1.5, adj=-0.145, cex=9/pointsize)

plot.coef(VincristinePredictor.cv,
          label.pct= 40.5,
          main = expression(paste("Regularisation Curves for Vincristine")))

mtext("(c)", 3,
      line=1.5, adj=-0.145, cex=9/pointsize)
```

For the α values resulting in the smallest cross validation error, the regularisation curves for the three drugs are plotted in the code chunk below. The plot is shown in Figure 6.3.

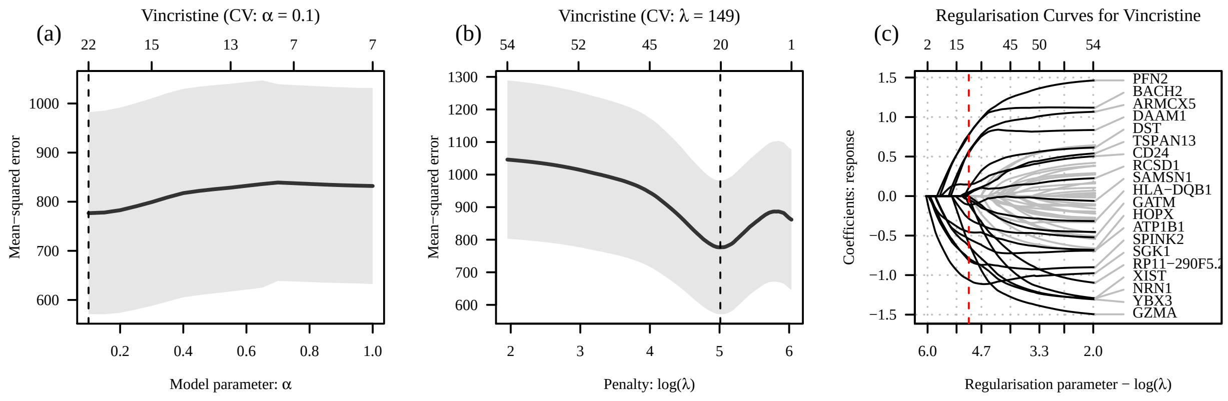

Figure 6.3: Cross validation and regularisation curves. In panel A the minimum mean squared error obtained for various α values are plotted. In panel B the mean-squared error is plotted against λ for the α value resulting in the minimum error and in Panel C the corresponding regularisation curves are shown. The genes for the 20 probe-sets associated with the largest coefficients are shown.

The minimum mean squared error for Vincristine was 776.67, which was obtained at α =0.1 and log(λ) =5. The

resulting GEP classifier for these parameters have non null coefficients for 22 genes.

The REGS predictor for vincristine is established below.

```
COEF <- as.matrix(coef(VincristinePredictor.cv, s = "lambda.min"))

Vincristine.reisitance.coef <- COEF[COEF != 0, ]

probes <- names(Vincristine.reisitance.coef)[c(-1, -length(Vincristine.reisitance.coef))]

sd.Vincristine <- apply(exprs(GEPCHO.sc[probes, ]), 1, function(x) sd(x, na.rm = TRUE))

Vincristine.reisitance.pred.coef <-
  Vincristine.reisitance.coef[-length(Vincristine.reisitance.coef)] *
  c(1, sd.Vincristine)

VincristinePredictor<- function(newx, coef = Vincristine.reisitance.pred.coef){

  x <- rbind(1, newx[names(coef)[-1],,drop= FALSE])

  t(x) %*% as.matrix((coef))
}
```

The REGS predictor is saved for later use.

```
save(VincristinePredictor, Vincristine.reisitance.pred.coef,
     file = "GeneratedData/REGS/VincristinePredictor.RData")
```

6.3 Establishment of REGS Classifier

```
cutFun <-function(x) {
  cut(x,
      breaks=c(-Inf, quantile(x, c(1/3, 2/3),na.rm = TRUE), Inf),
      labels = c("Sensitive", "Intermediate","Resistant"))
}
```

6.3.1 Cross Validation

```
file.Vincristine.class <-
  file.path("GeneratedData/REGS",
            paste("Vincristine_classification_genes.RData"))

if(file.exists(file.Vincristine.class)){
  load(file.Vincristine.class)
}else{

  mat <- matrix(NaN, ncol = 4, nrow = length(alphas))
  colnames(mat) <- paste("Vincristine",
                         c("", ".lo", ".up", ".n"), sep = "")
  row.names(mat) <- alphas

  ## plot all the cross validation plots for later inspection
  pdf(file.path(Vincristine.output, "cvplots_classification.pdf"))

  class.Vincristine.fit <- list()
```

```r
# The GEP data is aligned with the dose response
train.set <- exprs(GEPCHO.sc)


R.data <- summary.Vincristine[colnames(train.set), ]
R.data$Disease <- as.character(metadataHBCCL[colnames(train.set), "Disease"])

R.data$AUC.class <-
  factor("Intermediate",
         levels= c("Sensitive", "Intermediate", "Resistant"))

R.data[R.data$Disease == "DLBCL", "AUC.class"] <-
  cutFun(R.data[R.data$Disease == "DLBCL", "AUC"])

R.data[R.data$Disease == "MM", "AUC.class"] <-
  cutFun(R.data[R.data$Disease == "MM", "AUC"])

R.data$AUC.class <- as.character(R.data$AUC.class)

y <- as.factor(R.data[R.data[, "AUC.class"] %in%
                        c("Resistant","Sensitive") ,"AUC.class"])

names(y) <- rownames(R.data[R.data[, "AUC.class"] %in%
                              c("Resistant","Sensitive") ,"AUC.class", drop = FALSE])

drug <- "Vincristine"
for(alpha in alphas){
  cat(drug, alpha, "\n")

  fit <-
    cv.glmnet(x = t(train.set[, names(y)]),
              alpha        = alpha,
              grouped      = FALSE,
              type.measure = "class",
              family       = "multinomial",
              nfolds       = length(y),
              keep         = TRUE,
              y            = y,
              lambda       = exp(seq(-6,3, length.out = 600)),
              standardize  = FALSE,
              nlambda      = 600)



  plot(fit, main = paste(drug, alpha))

  wh.min <- length(fit$cvm) - which.min(rev(fit$cvm))
  mat[paste(alpha), drug] <- min(fit$cvm)
  mat[paste(alpha), paste(drug, ".lo", sep = "")] <-
    fit$cvlo[wh.min]
  mat[paste(alpha), paste(drug, ".up", sep = "")] <-
    fit$cvup[wh.min]
  mat[paste(alpha), paste(drug, ".n", sep = "")] <-
    fit$nzero[wh.min]

  class.Vincristine.fit[[paste(alpha)]] <- fit
```

```
  }

  dev.off()
  save(class.Vincristine.fit, mat, file = file.Vincristine.class)
}
```

The alpha value resulting in the smallest classication error is found in the code chunk below.

```
fit <- class.Vincristine.fit[[names(which.min((mat[, "Vincristine"])))]]
fit$lambda.min <-
  rev(fit$lambda)[which.min(rev(fit$cvm))]
VincristineClassifier.cv <- fit
```

The result of the cross validation is plotted in Figure 6.4. The code chunk below creates this plot.

```
par(mfrow = c(1,3))
drug <- "Vincristine"
plot.cv.alpha(x  = as.numeric(row.names(mat)),
              y  = mat[, drug],
              lo = mat[, paste(drug, ".lo", sep = "")],
              up = mat[, paste(drug, ".up", sep = "")],
              nzero = mat[,paste(drug, ".n", sep = "")],
              main1 = drug,
              las   = 1,
              ylab = "Misclassification error")

mtext("(a)", 3,
      line=1.5, adj=-0.145, cex=9/pointsize)

plot.cv.lambda(x  = log(VincristineClassifier.cv$lambda),
               y  = VincristineClassifier.cv$cvm,
               lo = VincristineClassifier.cv$cvlo,
               up = VincristineClassifier.cv$cvup,
               nzero = VincristineClassifier.cv$nzero,
               main1 = drug,
               las   = 1,
               ylab = "Misclassification error")

mtext("(b)", 3,
      line=1.5, adj=-0.145, cex=9/pointsize)
plot.coef(VincristineClassifier.cv,
          label.pct=36,
          main = paste("Regularisation curves for", drug))

mtext("(c)", 3,
      line=2, adj=-0.145, cex=9/pointsize)
```

The minimum classification error for Vincristine was 0.31 which was obtained at α =0.1 and $\log(\lambda)$ =0.54. The resulting GEP classifier for these parameters have non null coefficients for 32 genes. For the α value resulting in the smallest cross validation error, the regularisation curves are plotted in Figure 6.4 Panel C.

The REGS classifier for Vincristine is established below.

```
COEF <- as.matrix(coef(VincristineClassifier.cv, s = "lambda.min")$Resistant)

Vincristine.reisitance.coef <- COEF[COEF != 0, ]

probes <- names(Vincristine.reisitance.coef)[-1]
```

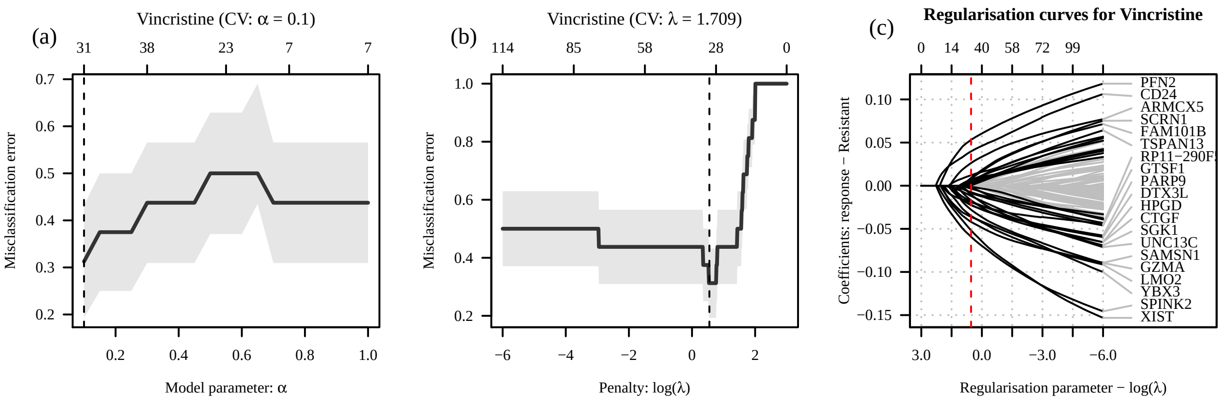

Figure 6.4: Cross validation and regularisation curves. In panel A the misclassification error obtained for various α values are plotted. In panel B the misclassification error is plotted against λ for the α value resulting in the minimum error and in Panel C the corresponding regularisation curves are shown. The genes for the 20 probe-sets associated with the largest coefficients are shown.

```
sd.Vincristine <- apply(exprs(GEPCHO.sc[probes, ]), 1, function(x) sd(x, na.rm = TRUE))

Vincristine.reisitance.class.coef <- Vincristine.reisitance.coef * c(1, sd.Vincristine)

VincristineClassifier <- function(newx, coef = Vincristine.reisitance.class.coef){

  x <- rbind(1, newx[names(coef)[-1],,drop= FALSE])

  prob <- t(x) %*% as.matrix((coef)  )

  prob <- exp(prob) / ( exp(prob) + exp(-prob))
  colnames(prob) <- "Resistant"
  prob
}
```

The REGS classifier is saved for later use.

```
save(VincristineClassifier, Vincristine.reisitance.class.coef,
     file = "GeneratedData/REGS/VincristineClassifier.RData")
```

```
## Error in parse_block(g[-1], g[1], params.src):  duplicate label 'unnamed-chunk-1'
```

Part II

Validation

All validation data are scaled according to the the function `microarrayScale`

```
microarrayScale <- function(x){
  x.m <- exprs(x)
  rowMedians <- rowMedians(x.m,  na.rm = TRUE)
  rowSd      <- rowSds(x.m, na.rm = TRUE)

  x.m        <-  x.m - rowMedians

  exprs(x)   <- x.m / rowSd
  return(x)
}
```

The continuous data are cut according to tertiles of the data.

```
probCut <- function(x, cut = c(1/3, 2/3))
  cut(x, breaks=c(-Inf, quantile(x, cut), Inf),
                          labels = c("Sensitive", "Intermediate", "Resistant"))
```

7 IDRC Cohort

The IDRC data consist of data from 470 patients with diffuse large B-cell lymphoma (DLBCL). The data include gene expressions from the Affymetrix GeneChip HG-U133 Plus 2.0 arrays and information regarding progression free survival (PFS). The .CEL files are available at

- `http://www.ncbi.nlm.nih.gov/geo/`

under GEO accession number GSE31312.

7.1 Loading the data

The metadata and RMA normalized GEP data for IDRC RCHOP are loaded into R.

```
load(file.path(Database,
               "DLBCL/IDRC_GSE31312/V1/Metadata/metadataIDRC.RData"))

load(file.path(Database,
               "DLBCL/IDRC_GSE31312/V1/",
               "PreProcessed/GEPIDRC_RMA_affy.RData"))
```

The GEP data are probeset wise median centred and scaled to have standard deviation 1.

```
GEPIDRC.sc <- microarrayScale(GEPIDRC)
```

7.2 REGS Classification

The data is classified according to REGS classifiers for Cyclophosphamide, Doxorubicin, and Vincristine.

```
metadataIDRC$C.prob <-
  CyclophosphamideClassifier(exprs(GEPIDRC.sc))[,1]

metadataIDRC$H.prob <-
  DoxorubicinClassifier(exprs(GEPIDRC.sc))[,1]

metadataIDRC$GCPH.prob <-
  GCPDoxorubicinClassifier(exprs(GEPIDRC.sc))[,1]

metadataIDRC$O.prob <-
  VincristineClassifier(exprs(GEPIDRC.sc))[,1]
```

```
test.mat <- metadataIDRC[, c("C.prob", "H.prob", "O.prob") ]

metadataIDRC$CHO.prob <-
  apply(test.mat, 1, prod) /
    (apply(test.mat, 1, prod) +
       apply(1-test.mat, 1, prod))
```

```
metadataIDRC$C.class    <- probCut(metadataIDRC$C.prob)
metadataIDRC$H.class    <- probCut(metadataIDRC$H.prob)
metadataIDRC$GCPH.class <- probCut(metadataIDRC$GCPH.prob)
metadataIDRC$O.class    <- probCut(metadataIDRC$O.prob)
metadataIDRC$CHO.class  <- probCut(metadataIDRC$CHO.prob)
```

Table 7.1: Cox proportional hazards analyses of the association between PFS and OS and the classification of the clinical cohorts for CHO and the three individual drugs. In the multivariate analysis the Cox proportional hazards regression is adjusted for IPI. The estimated HR's compare patients classified as resistant to patients classified as sensitive.

	Univariate			Multivariate		
	N	HR (95% CI)	P-value	N	HR (95% CI)	P-value
Cyclophosphamide	470	1.03 (0.72,1.47)	0.864	424	0.98 (0.68,1.42)	0.92
Doxorubicin	470	2.58 (1.72,3.86)	4.37e-06	424	2.52 (1.64,3.87)	2.65e-05
Vincristine	470	1.80 (1.23,2.64)	0.00241	424	1.50 (1.01,2.23)	0.0454
CHO	470	2.42 (1.63,3.59)	1.24e-05	424	2.24 (1.46,3.44)	0.000222

7.2.1 Cox Proportional Hazards Regression

The resistance levels assigned to the patients of the IDRC cohort are investigated by Cox proportional hazards models. First a matrix containing the results are created.

```
cox.IDRC.prob <- matrix(0, nrow = 4, ncol = 3*2)
rownames(cox.IDRC.prob) <- c( "Cyclophosphamide", "Doxorubicin", "Vincristine", "CHO")

colnames(cox.IDRC.prob) <- rep(c("N", "HR (95\\% CI)", "P-value"), 2)
```

Second, the univariate Cox regression analysis is performed.

```
cox.IDRC.prob[1, 1:3] <- CalcHR2(PFS5 ~ C.class,
                                 data = metadataIDRC)
cox.IDRC.prob[2, 1:3] <- CalcHR2(PFS5 ~ H.class,
                                 data = metadataIDRC)
cox.IDRC.prob[3, 1:3] <- CalcHR2(PFS5 ~ O.class,
                                 data = metadataIDRC)
cox.IDRC.prob[4, 1:3] <- CalcHR2(PFS5 ~ CHO.class,
                                 data = metadataIDRC)
```

Third, the multivariate Cox regression analysis is performed.

```
cox.IDRC.prob[1, 4:6] <- CalcHR2(PFS5 ~ C.class + ipi.hl,
                                 data = metadataIDRC)
cox.IDRC.prob[2, 4:6] <- CalcHR2(PFS5 ~ H.class + ipi.hl,
                                 data = metadataIDRC)
cox.IDRC.prob[3, 4:6] <- CalcHR2(PFS5 ~ O.class + ipi.hl,
                                 data = metadataIDRC)
cox.IDRC.prob[4, 4:6] <- CalcHR2(PFS5 ~ CHO.class + ipi.hl,
                                 data = metadataIDRC)
```

The results are shown in Table 7.1.

7.2.2 Kaplan-Meier Analysis

Patients in each of the clinical cohorts were assigned a probability of being sensitive to each of the three drugs. In this section these probabilities are plotted along with areas determining the three categories sensitive, intermeidate, and resistant. Kaplan Meier curves are also established according to the categorisations and the hazard rate ratios obtained between sensitive:intermediate and sensitive:resistant are estimated. For the cohort IDRC this is done in the code chunk below. The resulting plots are shown in Figure 7.1.

```
addAreaColour <- function(prob, col.code){
  polygon(x = c(0, 0, rep(length(prob), 2)),
          y = c(quantile(prob, 2/3), 1, 1,
                quantile(prob, 2/3)),
          col = paste(col.code[1], 60, sep = ""),
          border = 0)
```

```
  polygon(x = c(0, 0, rep(length(prob), 2)),
          y = c(quantile(prob, 1/3),
                quantile(prob, 2/3),
                quantile(prob, 2/3),
                quantile(prob, 1/3)),
          col = paste(col.code[2], 60, sep = ""),,
          border = 0)

  polygon(x = c(0, 0, rep(length(prob), 2)),
          y = c(quantile(prob, 1/3),
                0,0,
                quantile(prob, 1/3)),
          col = paste(col.code[3], 60, sep = ""),,
          border = 0)

  segments(x0=0,y0= quantile(prob, 1/3), x1=length(prob),
           y1 =quantile(prob, 1/3) , col = "white", lwd = 4)
  segments(x0=0,y0= quantile(prob, 2/3), x1=length(prob),
           y1 =quantile(prob, 2/3), col = "white", lwd = 4)
}
```

```
par(mfrow= c(2, 4))
par(mar = c(5.1, 2.8, 4.1, 1))
par(oma = c(0, 3.1, 0, 0))

drugs <- c("C", "H", "O", "CHO")
names(drugs) <- c("Cyclophosphamide", "Doxorubicin", "Vincristine", "CHO")
for(i in 1:length(drugs)){
  drug <- drugs[i]

  par(mfg = c(1, i))

  prob <- 1-metadataIDRC[, paste(drug, "prob", sep = ".")]

  plot(sort(prob), col = 0,   las = 1,
       xlab = "Samples", ylab = "Probablity of sensitivity",
       main = paste("IDRC,", names(drug)))

  addAreaColour(prob, col.code)

  points(sort(prob))

  if(i == 1)
    mtext("Probablity of sensitivity",outer = TRUE,side = 2,adj=0.87)

  mtext(paste0("(", letters[i], ")"), 3,
        line=1.5, adj=-0.145, cex=9/pointsize)

  table(metadataIDRC[, paste(drug, "class", sep = ".")], useNA ="ifany")

  par(mfg = c(2, i))

  formula <- as.formula(paste("PFS5 ~ ", drug, ".class", sep = ""))
  plot(survfit(formula, data = metadataIDRC),
       col   = col.code[
         levels(metadataIDRC[, paste(drug, ".class", sep = "")])],
       xlab = "Progression free survival (years)",
       ylab = "Survival probability",
```

```
      las = 1,
      main = paste("IDRC,", names(drug)), lwd = 1.3)

  legend("bottomleft", lty = 1,
         col = col.code[levels(
           metadataIDRC[, paste(drug, ".class", sep = "")])],
         legend = levels(metadataIDRC[, paste(drug, "class", sep = ".")]),
         bty = "n")

  cox.fit <- coxph(formula, data = metadataIDRC)
  cox.sum <- summary(cox.fit)

  legend("bottomright",
         paste(c("I:S ", "R:S "), c("Hz: ", "Hz: ") ,
               round2(cox.sum$coefficients[,2], 2), #c(" CI ", " CI ") ,
               c(" (", " ("),
               round2(cox.sum$conf.int[,3], 2), c(", ", ", "),
               round2(cox.sum$conf.int[,4], 2),
               c(")", ")"),
               sep = ""),
         bty = "n")

  if(i == 1)
    mtext("Survival probability",outer = TRUE, side = 2, adj =0.18 )

  mtext(paste0("(", letters[i + 4], ")"), 3,
        line=1.5, adj=-0.145, cex=9/pointsize)

}
```

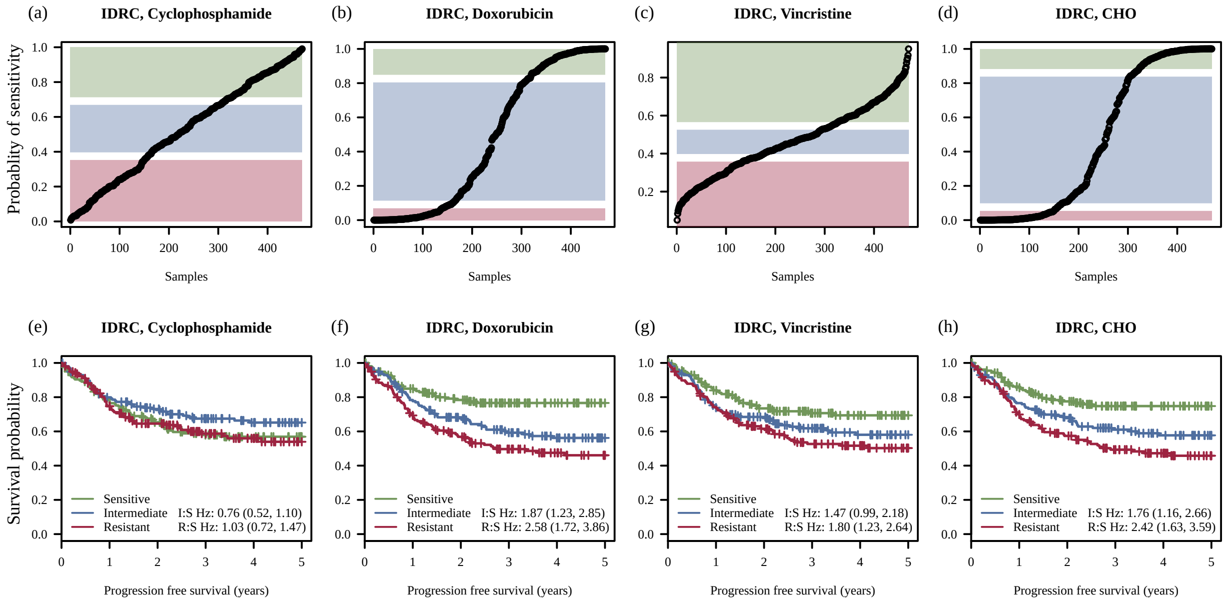

Figure 7.1: The classification of IDRC into sensitive, intermediate, and resistant patients. In panels A, B, C, and D the probability of being sensitive is plotted for each patient. Based on the probabilities the patients are categorised into tertiles with those deemed sensitive intermediate, and resistant indicated by green, blue, and red. Kaplan-Meier curves for PFS are shown in panels E, F, G, and H.

7.3 REGS Prediction

Resistance indices are established according to REGS predictors for Cyclophosphamide, Doxorubicin, Vincristine.

```
metadataIDRC$C.pred <-
  CyclophosphamidePredictor(exprs(GEPIDRC.sc))[,1]

metadataIDRC$H.pred <-
  DoxorubicinPredictor(exprs(GEPIDRC.sc))[,1]

metadataIDRC$GCPH.pred <-
  GCPDoxorubicinPredictor(exprs(GEPIDRC.sc))[,1]

metadataIDRC$O.pred <-
  VincristinePredictor(exprs(GEPIDRC.sc))[,1]
```

The resistance indices are combined using the geometric mean.

```
metadataIDRC$CHO.pred <- apply(metadataIDRC[, c("C.pred", "H.pred", "O.pred") ],
                               1, function(x) geomean(x))
```

7.3.1 Cox Proportional Hazards Regression

The resistance levels assigned to the patients of the IDRC cohort are investigated by Cox proportional hazards models. First a matrix containing the results are created.

```
cox.IDRC.pred <- matrix(0, nrow = 4, ncol = 3*2)
rownames(cox.IDRC.pred) <- c( "Cyclophosphamide", "Doxorubicin", "Vincristine", "CHO")

colnames(cox.IDRC.pred) <- rep(c("N", "HR (95\\% CI)", "P-value"), 2)
```

Second, the univariate Cox regression analysis is performed.

```
cox.IDRC.pred[1, 1:3] <- CalcHR(PFS5 ~ I(C.pred/10),
                                data = metadataIDRC)
cox.IDRC.pred[2, 1:3] <- CalcHR(PFS5 ~ I(H.pred/10),
                                data = metadataIDRC)
cox.IDRC.pred[3, 1:3] <- CalcHR(PFS5 ~ I(O.pred/10),
                                data = metadataIDRC)
cox.IDRC.pred[4, 1:3] <- CalcHR(PFS5 ~ I(CHO.pred/10),
                                data = metadataIDRC)
```

Third, the multivariate Cox regression analysis is performed.

```
cox.IDRC.pred[1, 4:6] <- CalcHR(PFS5 ~ I(C.pred/10) + ipi.hl,
                                data = metadataIDRC)
cox.IDRC.pred[2, 4:6] <- CalcHR(PFS5 ~ I(H.pred/10) + ipi.hl,
                                data = metadataIDRC)
cox.IDRC.pred[3, 4:6] <- CalcHR(PFS5 ~ I(O.pred/10) + ipi.hl,
                                data = metadataIDRC)
cox.IDRC.pred[4, 4:6] <- CalcHR(PFS5 ~ I(CHO.pred/10) + ipi.hl,
                                data = metadataIDRC)
```

The results are shown in Table 7.2.

7.3.2 Cox Proportional Hazards Regression based on Restricted Cubic Splines

In the following code chunk a restricted cubic spline with four knots is fitted to the resistance index. Based on the fitted spline, survival curves, adjusted for IPI are generated. The resulting plot is shown in Figure 7.2.

Table 7.2: Cox proportional hazards analyses of the association between PFS and OS and the predicted resistance indices for CHO and the three individual drugs. In the multivariate analyses the Cox proportional hazards regressions are adjusted for IPI. The estimated HR's are based on an increase of 10 in the AUC_0

	Univariate			Multivariate		
	N	HR (95% CI)	P-value	N	HR (95% CI)	P-value
Cyclophosphamide	470	0.98 (0.92,1.03)	0.371	424	0.98 (0.93,1.04)	0.598
Doxorubicin	470	1.11 (1.04,1.17)	0.000572	424	1.09 (1.03,1.16)	0.00595
Vincristine	470	1.56 (1.26,1.94)	5.48e-05	424	1.49 (1.19,1.87)	0.000554
CHO	470	1.24 (1.09,1.42)	0.00153	424	1.23 (1.06,1.42)	0.00555

```
n.knots <- 4
drugs <- c("C.pred", "H.pred", "O.pred", "CHO.pred")

par(mfrow = c(2, length(drugs)))
par(mar = c(5.1, 2.8, 4.1, 1))
par(oma = c(0, 3.1, 0, 0))

for(i in 1:length(drugs)){
  drug <- drugs[i]
  drug.i <- metadataIDRC[, drug]
  ipi <- as.factor(metadataIDRC$ipi.hl)
  d <- datadist(drug.i, ipi)
  options(datadist = "d", width = 150)


  main = paste("IDRC,", ifelse(grepl("Combined" , drug), "Combined", drug))

  fit <- cph(metadataIDRC$PFS5  ~ rcs(drug.i, n.knots) + ipi, surv = TRUE,
             x= TRUE, y = TRUE)

  seq(sort(drug.i)[11], sort(drug.i)[length(drug.i)- 10], length.out = 5)


  par(mfg = c(1,i))
  p <- Predict(fit, drug.i=seq(min(drug.i), max(drug.i), length.out=20),
               ipi = "2-3", #age = median(age),
               fun=exp)

  plot.cv(x = p$drug.i, y = p$yhat, lo = p$lower, up=p$upper,
          main = main,
          las  = 1,
          ylab = "",
          xlab = "Area under dose response curve")

    marks <- seq(sort(drug.i)[11], sort(drug.i)[length(drug.i)- 10], length.out = 5)
  r <- range(c(log(p$lower), log(p$upper)))
  r <- r[2] - r[1]
  arrows(marks, rep(min(log(p$lower) + r/10, 5)), marks, rep(min(log(p$lower), 5)),
         length = 0.05, col = col.data$col)

  if(i == 1)
    mtext("Log relative hazard",outer = TRUE,side = 2,adj=0.87)

  mtext(paste0("(", letters[i], ")"), 3,
        line=1.5, adj=-0.145, cex=9/pointsize)
  par(mfg = c(2, i))
```

```
  plot(0,0, ylim = c(0,1), xlim = c(0,5), col = 0,
      xlab = "Overall survival (years)",
      ylab = "", las = 1)

  survplot(fit, drug.i, ipi = "2-3",
           col.fill=paste(col.data$col, 50, sep = ""),
           col=col.data$col, lwd = 2,
           lty = 1, label.curves=FALSE,
           add  = TRUE)

  legend("bottomleft", lty = 1, col = col.data$col[1:5], legend = col.data$cell[1:5],
         bty = "n")

  title(main, line=3)
  n.risk <- fit$surv.summary[, , "n.risk"]
  axis(side=3,at= names(n.risk), labels=n.risk)
  graphics:::box()

  if(i == 1)
    mtext("Survival probability",outer = TRUE, side = 2, adj =0.18 )

  mtext(paste0("(", letters[i + 4], ")"), 3,
        line=1.5, adj=-0.145, cex=9/pointsize)
}

## Error:  could not find function "datadist"
```

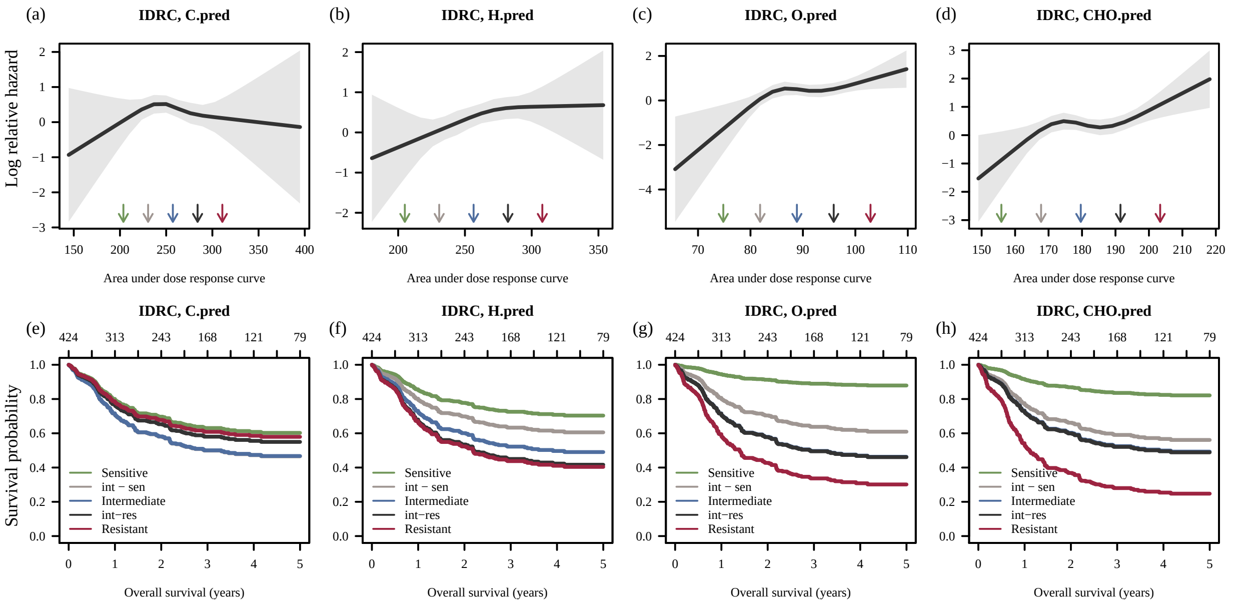

Figure 7.2: Fitted restricted cubic spline to the IDRC cohort. In panels A, B, C, and D the fitted spline is shown. Generated survival curves for PFS are shown in panels E, F, G, and H.

8 LLMPP Cohort

The LLMPP data consist of data from 233 patients with diffuse large B-cell lymphoma (DLBCL). The data include gene expressions from the Affymetrix GeneChip HG-U133 Plus 2.0 arrays and information regarding progression free survival (PFS). The metadata and .CEL files are available at

- `http://www.ncbi.nlm.nih.gov/geo/`

under GEO accession number GSE10846. The PFS was made available by personal communication with Lenz.

8.1 Loading the data

The metadata and RMA normalized GEP data for LLMPP RCHOP are loaded into R.

```
load(file.path(Database,
               "DLBCL/LLMPP_GSE10846/V1/GeneratedData/Metadata/metadataLLMPPRCHOP.RData"))

load(file.path(Database,
               "DLBCL/LLMPP_GSE10846/V1/",
               "GeneratedData/GEP/GEPLLMPPRCHOP_RMA_affy.RData"))
```

The GEP data are probeset wise median centred and scaled to have standard deviation 1.

```
GEPLLMPPRCHOP.sc <- microarrayScale(GEPLLMPPRCHOP)
```

8.2 REGS Classification

The data is classified according to REGS classifiers for Cyclophosphamide, Doxorubicin, and Vincristine.

```
metadataLLMPPRCHOP$C.prob <-
  CyclophosphamideClassifier(exprs(GEPLLMPPRCHOP.sc))[,1]

metadataLLMPPRCHOP$H.prob <-
  DoxorubicinClassifier(exprs(GEPLLMPPRCHOP.sc))[,1]

metadataLLMPPRCHOP$GCPH.prob <-
  GCPDoxorubicinClassifier(exprs(GEPLLMPPRCHOP.sc))[,1]

metadataLLMPPRCHOP$O.prob <-
  VincristineClassifier(exprs(GEPLLMPPRCHOP.sc))[,1]
```

```
test.mat <- metadataLLMPPRCHOP[, c("C.prob", "H.prob", "O.prob") ]

metadataLLMPPRCHOP$CHO.prob <-
  apply(test.mat, 1, prod) /
    (apply(test.mat, 1, prod) +
       apply(1-test.mat, 1, prod))
```

```
metadataLLMPPRCHOP$C.class    <- probCut(metadataLLMPPRCHOP$C.prob)
metadataLLMPPRCHOP$H.class    <- probCut(metadataLLMPPRCHOP$H.prob)
metadataLLMPPRCHOP$GCPH.class <- probCut(metadataLLMPPRCHOP$GCPH.prob)
metadataLLMPPRCHOP$O.class    <- probCut(metadataLLMPPRCHOP$O.prob)
metadataLLMPPRCHOP$CHO.class  <- probCut(metadataLLMPPRCHOP$CHO.prob)
```

Table 8.1: Cox proportional hazards analyses of the association between PFS and OS and the classification of the clinical cohorts for CHO and the three individual drugs. In the multivariate analysis the Cox proportional hazards regression is adjusted for IPI. The estimated HR's compare patients classified as resistant to patients classified as sensitive.

	Univariate			Multivariate		
	N	HR (95% CI)	P-value	N	HR (95% CI)	P-value
Cyclophosphamide	220	0.77 (0.41,1.43)	0.403	180	1.06 (0.53,2.12)	0.859
Doxorubicin	220	2.28 (1.10,4.73)	0.0269	180	2.52 (1.13,5.64)	0.0237
Vincristine	220	3.05 (1.51,6.15)	0.00184	180	2.56 (1.22,5.39)	0.0134
CHO	220	2.01 (0.96,4.19)	0.0633	180	2.43 (1.05,5.59)	0.0374

8.2.1 Cox Proportional Hazards Regression

The resistance levels assigned to the patients of the LLMPP cohort are investigated by Cox proportional hazards models. First a matrix containing the results are created.

```
cox.LLMPP.prob <- matrix(0, nrow = 4, ncol = 3*2)
rownames(cox.LLMPP.prob) <- c( "Cyclophosphamide", "Doxorubicin", "Vincristine", "CHO")

colnames(cox.LLMPP.prob) <- rep(c("N", "HR (95\\% CI)", "P-value"), 2)
```

Second, the univariate Cox regression analysis is performed.

```
cox.LLMPP.prob[1, 1:3] <- CalcHR2(PFS5 ~ C.class,
                                  data = metadataLLMPPRCHOP)
cox.LLMPP.prob[2, 1:3] <- CalcHR2(PFS5 ~ H.class,
                                  data = metadataLLMPPRCHOP)
cox.LLMPP.prob[3, 1:3] <- CalcHR2(PFS5 ~ O.class,
                                  data = metadataLLMPPRCHOP)
cox.LLMPP.prob[4, 1:3] <- CalcHR2(PFS5 ~ CHO.class,
                                  data = metadataLLMPPRCHOP)
```

Third, the multivariate Cox regression analysis is performed.

```
cox.LLMPP.prob[1, 4:6] <- CalcHR2(PFS5 ~ C.class + ipi.hl2,
                                  data = metadataLLMPPRCHOP)
cox.LLMPP.prob[2, 4:6] <- CalcHR2(PFS5 ~ H.class + ipi.hl2,
                                  data = metadataLLMPPRCHOP)
cox.LLMPP.prob[3, 4:6] <- CalcHR2(PFS5 ~ O.class + ipi.hl2,
                                  data = metadataLLMPPRCHOP)
cox.LLMPP.prob[4, 4:6] <- CalcHR2(PFS5 ~ CHO.class + ipi.hl2,
                                  data = metadataLLMPPRCHOP)
```

The results are shown in Table 8.1.

8.2.2 Kaplan-Meier Analysis

Patients in each of the clinical cohorts were assigned a probability of being sensitive to each of the three drugs. In this section these probabilities are plotted along with areas determining the three categories sensitive, intermeidate, and resistant. Kaplan Meier curves are also established according to the categorisations and the hazard rate ratios obtained between sensitive:intermediate and sensitive:resistant are estimated. For the cohort LLMPP this is done in the code chunk below. The resulting plots are shown in Figure 8.1.

```
addAreaColour <- function(prob, col.code){
  polygon(x = c(0, 0, rep(length(prob), 2)),
          y = c(quantile(prob, 2/3), 1, 1,
                quantile(prob, 2/3)),
          col = paste(col.code[1], 60, sep = ""),
          border = 0)
```

```
  polygon(x = c(0, 0, rep(length(prob), 2)),
          y = c(quantile(prob, 1/3),
                quantile(prob, 2/3),
                quantile(prob, 2/3),
                quantile(prob, 1/3)),
          col = paste(col.code[2], 60, sep = ""),,
          border = 0)

  polygon(x = c(0, 0, rep(length(prob), 2)),
          y = c(quantile(prob, 1/3),
                0,0,
                quantile(prob, 1/3)),
          col = paste(col.code[3], 60, sep = ""),,
          border = 0)

  segments(x0=0,y0= quantile(prob, 1/3), x1=length(prob),
           y1 =quantile(prob, 1/3) , col = "white", lwd = 4)
  segments(x0=0,y0= quantile(prob, 2/3), x1=length(prob),
           y1 =quantile(prob, 2/3), col = "white", lwd = 4)
}
```

```
par(mfrow= c(2, 4))
par(mar = c(5.1, 2.8, 4.1, 1))
par(oma = c(0, 3.1, 0, 0))

drugs <- c("C", "H", "O", "CHO")
names(drugs) <- c("Cyclophosphamide", "Doxorubicin", "Vincristine", "CHO")
for(i in 1:length(drugs)){
  drug <- drugs[i]

  par(mfg = c(1, i))

  prob <- 1-metadataLLMPPRCHOP[, paste(drug, "prob", sep = ".")]

  plot(sort(prob), col = 0,   las = 1,
       xlab = "Samples", ylab = "Probablity of sensitivity",
       main = paste("LLMPP,", names(drug)))

  addAreaColour(prob, col.code)

  points(sort(prob))

  if(i == 1)
    mtext("Probablity of sensitivity",outer = TRUE,side = 2,adj=0.87)

  mtext(paste0("(", letters[i], ")"), 3,
        line=1.5, adj=-0.145, cex=9/pointsize)

  table(metadataLLMPPRCHOP[, paste(drug, "class", sep = ".")], useNA ="ifany")

  par(mfg = c(2, i))

  formula <- as.formula(paste("PFS5 ~ ", drug, ".class", sep = ""))
  plot(survfit(formula, data = metadataLLMPPRCHOP),
       col   = col.code[
         levels(metadataLLMPPRCHOP[, paste(drug, ".class", sep = "")])],
       xlab = "Progression free survival (years)",
       ylab = "Survival probability",
```

```
      las = 1,
      main = paste("LLMPP,", names(drug)), lwd = 1.3)

  legend("bottomleft", lty = 1,
         col = col.code[levels(
           metadataLLMPPRCHOP[, paste(drug, ".class", sep = "")])],
         legend = levels(metadataLLMPPRCHOP[, paste(drug, "class", sep = ".")]),
         bty = "n")

  cox.fit <- coxph(formula, data = metadataLLMPPRCHOP)
  cox.sum <- summary(cox.fit)

  legend("bottomright",
         paste(c("I:S ", "R:S "), c("Hz: ", "Hz: ") ,
               round2(cox.sum$coefficients[,2], 2), #c(" CI ", " CI ") ,
               c(" (", " ("),
               round2(cox.sum$conf.int[,3], 2), c(", ", ", "),
               round2(cox.sum$conf.int[,4], 2),
               c(")", ")"),
               sep = ""),
         bty = "n")

  if(i == 1)
    mtext("Survival probability",outer = TRUE, side = 2, adj =0.18 )

  mtext(paste0("(", letters[i + 4], ")"), 3,
        line=1.5, adj=-0.145, cex=9/pointsize)

}
```

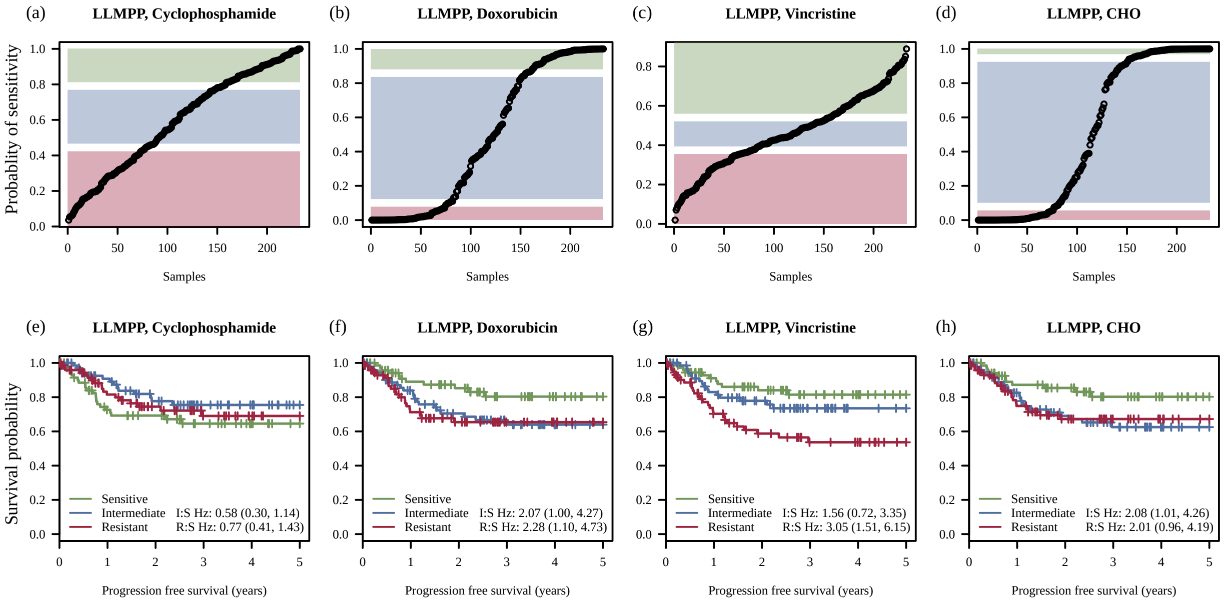

Figure 8.1: The classification of LLMPP into sensitive, intermediate, and resistant patients. In panels A, B, C, and D the probability of being sensitive is plotted for each patient. Based on the probabilities the patients are categorised into tertiles with those deemed sensitive intermediate, and resistant indicated by green, blue, and red. Kaplan-Meier curves for PFS are shown in panels E, F, G, and H.

8.3 REGS Prediction

Resistance indices are established according to REGS predictors for Cyclophosphamide, Doxorubicin, Vincristine.

```
metadataLLMPPRCHOP$C.pred <-
  CyclophosphamidePredictor(exprs(GEPLLMPPRCHOP.sc))[,1]

metadataLLMPPRCHOP$H.pred <-
  DoxorubicinPredictor(exprs(GEPLLMPPRCHOP.sc))[,1]

metadataLLMPPRCHOP$GCPH.pred <-
  GCPDoxorubicinPredictor(exprs(GEPLLMPPRCHOP.sc))[,1]

metadataLLMPPRCHOP$O.pred <-
  VincristinePredictor(exprs(GEPLLMPPRCHOP.sc))[,1]
```

The resistance indices are combined using the geometric mean.

```
metadataLLMPPRCHOP$CHO.pred <- apply(metadataLLMPPRCHOP[, c("C.pred", "H.pred", "O.pred") ], 1, functio
```

8.3.1 Cox Proportional Hazards Regression

The resistance levels assigned to the patients of the LLMPP cohort are investigated by Cox proportional hazards models. First a matrix containing the results are created.

```
cox.LLMPP.pred <- matrix(0, nrow = 4, ncol = 3*2)
rownames(cox.LLMPP.pred) <- c( "Cyclophosphamide", "Doxorubicin", "Vincristine", "CHO")

colnames(cox.LLMPP.pred) <- rep(c("N", "HR (95\\% CI)", "P-value"), 2)
```

Second, the univariate Cox regression analysis is performed.

```
cox.LLMPP.pred[1, 1:3] <- CalcHR(PFS5 ~ I(C.pred/10),
                          data = metadataLLMPPRCHOP)
cox.LLMPP.pred[2, 1:3] <- CalcHR(PFS5 ~ I(H.pred/10),
                          data = metadataLLMPPRCHOP)
cox.LLMPP.pred[3, 1:3] <- CalcHR(PFS5 ~ I(O.pred/10),
                          data = metadataLLMPPRCHOP)
cox.LLMPP.pred[4, 1:3] <- CalcHR(PFS5 ~ I(CHO.pred/10),
                          data = metadataLLMPPRCHOP)
```

Third, the multivariate Cox regression analysis is performed.

```
cox.LLMPP.pred[1, 4:6] <- CalcHR(PFS5 ~ I(C.pred/10) + ipi.hl2,
                          data = metadataLLMPPRCHOP)
cox.LLMPP.pred[2, 4:6] <- CalcHR(PFS5 ~ I(H.pred/10) + ipi.hl2,
                          data = metadataLLMPPRCHOP)
cox.LLMPP.pred[3, 4:6] <- CalcHR(PFS5 ~ I(O.pred/10) + ipi.hl2,
                          data = metadataLLMPPRCHOP)
cox.LLMPP.pred[4, 4:6] <- CalcHR(PFS5 ~ I(CHO.pred/10) + ipi.hl2,
                          data = metadataLLMPPRCHOP)
```

The results are shown in Table 8.2.

8.3.2 Cox Proportional Hazards Regression based on Restricted Cubic Splines

In the following code chunk a restricted cubic spline with four knots is fitted to the resistance index. Based on the fitted spline survival curves adjusted for IPI are generated. The resulting plot is shown in Figure 8.2.

Table 8.2: Cox proportional hazards analyses of the association between PFS and OS and the predicted resistance indices for CHO and the three individual drugs. In the multivariate analyses the Cox proportional hazards regressions are adjusted for IPI. The estimated HR's are based on an increase of 10 in the AUC_0

	Univariate			Multivariate		
	N	HR (95% CI)	P-value	N	HR (95% CI)	P-value
Cyclophosphamide	220	0.95 (0.87,1.05)	0.315	180	0.96 (0.87,1.07)	0.511
Doxorubicin	220	1.10 (1.00,1.20)	0.0438	180	1.11 (1.00,1.23)	0.0433
Vincristine	220	1.99 (1.36,2.92)	0.000399	180	1.83 (1.22,2.74)	0.00341
CHO	220	1.18 (0.98,1.44)	0.0876	180	1.20 (0.97,1.48)	0.0885

```
n.knots <- 4
drugs <- c("C.pred", "H.pred", "O.pred", "CHO.pred")

par(mfrow = c(2, length(drugs)))
par(mar = c(5.1, 2.8, 4.1, 1))
par(oma = c(0, 3.1, 0, 0))

for(i in 1:length(drugs)){
  drug <- drugs[i]
  drug.i <- metadataLLMPPRCHOP[, drug]
  ipi <- as.factor(metadataLLMPPRCHOP$ipi.hl2)
  d <- datadist(drug.i, ipi)
  options(datadist = "d", width = 150)


  main = paste("LLMPP,", ifelse(grepl("Combined" , drug), "Combined", drug))

  fit <- cph(metadataLLMPPRCHOP$PFS5  ~ rcs(drug.i, n.knots) + ipi, surv = TRUE,
             x= TRUE, y = TRUE)

  seq(sort(drug.i)[11], sort(drug.i)[length(drug.i)- 10], length.out = 5)


  par(mfg = c(1,i))
  p <- Predict(fit, drug.i=seq(min(drug.i), max(drug.i), length.out=20),
               ipi = "2-3", #age = median(age),
               fun=exp)

  plot.cv(x = p$drug.i, y = p$yhat, lo = p$lower, up=p$upper,
          main = main,
          las  = 1,
          ylab = "log relative hazard",
          xlab = "Area under dose response curve")

    marks <- seq(sort(drug.i)[11], sort(drug.i)[length(drug.i)- 10], length.out = 5)
  r <- range(c(log(p$lower), log(p$upper)))
  r <- r[2] - r[1]
  arrows(marks, rep(min(log(p$lower) + r/10, 5)), marks, rep(min(log(p$lower), 5)),
         length = 0.05, col = col.data$col)

  if(i == 1)
    mtext("Log relative hazard",outer = TRUE,side = 2,adj=0.87)

  mtext(paste0("(", letters[i], ")"), 3,
        line=1.5, adj=-0.145, cex=9/pointsize)

  par(mfg = c(2, i))
```

```
  plot(0,0, ylim = c(0,1), xlim = c(0,5), col = 0,
       xlab = "Overall survival (years)",
       ylab = "Survival probability", las = 1)

  survplot(fit, drug.i, ipi = "2-3",
           col.fill=paste(col.data$col, 50, sep = ""),
           col=col.data$col, lwd = 2,
           lty = 1, label.curves=FALSE,
           add  = TRUE)

  legend("bottomleft", lty = 1, col = col.data$col[1:5], legend = col.data$cell[1:5],
         bty = "n")

  title(main, line=3)
  n.risk <- fit$surv.summary[, , "n.risk"]
  axis(side=3,at= names(n.risk), labels=n.risk)
  graphics:::box()

  if(i == 1)
    mtext("Survival probability",outer = TRUE, side = 2, adj =0.18 )

  mtext(paste0("(", letters[i + 4], ")"), 3,
        line=1.5, adj=-0.145, cex=9/pointsize)
}

## Error:  could not find function "datadist"
```

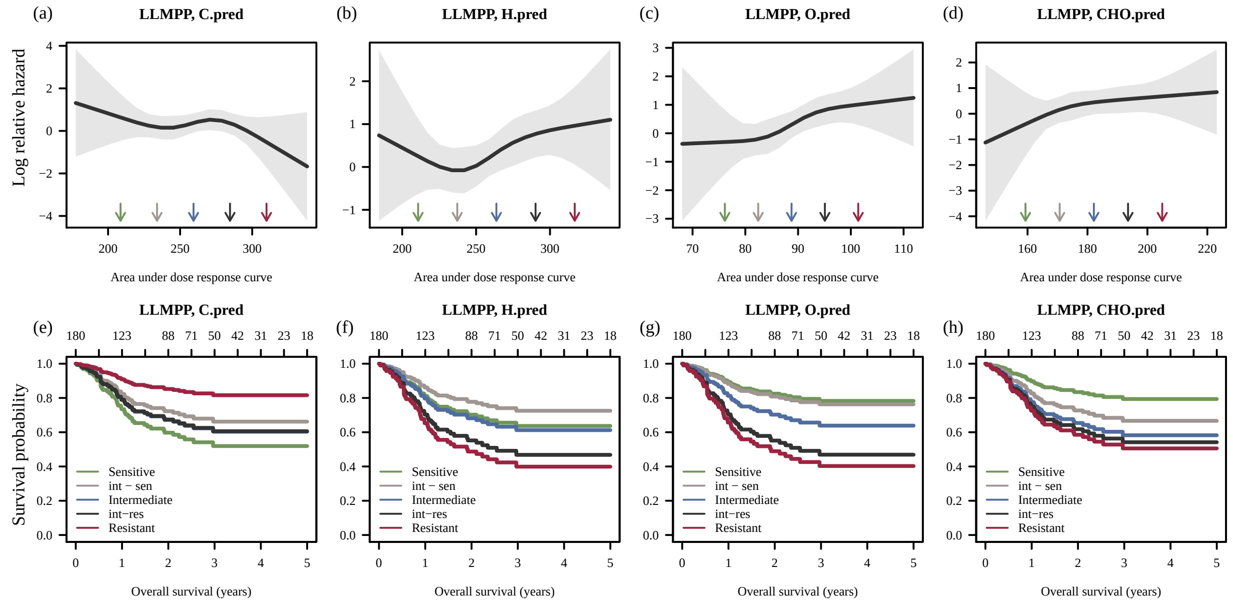

Figure 8.2: Fitted restricted cubic spline to the LLMPP cohort. In panels A, B, C, and D the fitted spline is shown. Generated survival curves for PFS are shown in panels E, F, G, and H.

9 IDRC and LLMPP Combined

The data sets are combined.

```
metadataLLMPPRCHOP2 <- metadataLLMPPRCHOP
metadataLLMPPRCHOP2$ipi.hl <- metadataLLMPPRCHOP2$ipi.hl2

int <- intersect(colnames(metadataIDRC), colnames(metadataLLMPPRCHOP2)) %w/o% "LDH"

metadataCombined <-
  rbind(cbind(metadataIDRC[,int],
              Data = "IDRC"),
        cbind(metadataLLMPPRCHOP2[, int],
              Data = "LLMPP"))

metadataCombined$PFS5 <- Surv(metadataCombined$PFS5[,1], metadataCombined$PFS5[,2])
```

9.1 Cox proportional hazard rate ratios

Cox proportional hazards analyses is performed to determine if the BAGS classification is of prognostic value. First we test for whether or not the cohort origin need to be accounted for with PFS as endpoint. This is initially done using both REGS classifier.

```
cox.full <- coxph(PFS5 ~ CHO.class + ipi.hl + Data, data = metadataCombined)
cox.A    <- coxph(PFS5 ~ CHO.class + ipi.hl, data = metadataCombined)
anova(cox.full, cox.A)

## Analysis of Deviance Table
##  Cox model: response is  PFS5
##  Model 1: ~ CHO.class + ipi.hl + Data
##  Model 2: ~ CHO.class + ipi.hl
##            loglik   Chisq Df P(>|Chi|)
## 1 -1187.121324557
## 2 -1187.891002369 1.53936  1   0.21471
```

Next it is done for the REGS predictor.

```
cox.full <- coxph(PFS5 ~ CHO.pred + ipi.hl + Data, data = metadataCombined)
cox.A    <- coxph(PFS5 ~ CHO.pred + ipi.hl, data = metadataCombined)
anova(cox.full, cox.A)

## Analysis of Deviance Table
##  Cox model: response is  PFS5
##  Model 1: ~ CHO.pred + ipi.hl + Data
##  Model 2: ~ CHO.pred + ipi.hl
##            loglik   Chisq Df P(>|Chi|)
## 1 -1192.781068951
## 2 -1193.847543633 2.13295  1   0.14416
```

Since this is not the case the coxproportional hazard rates are estimated for OS and PFS without accouting for cohort.
In the code chunk below it is tested whether there is a nonlinear relationship between prognosis and the resistance index given by the REGS predictor.

```
fit.spline <- coxph(PFS5  ~ rcs(CHO.pred) + ipi.hl, data = metadataCombined)

## Error in eval(expr, envir, enclos):  could not find function "rcs"
```

```
fit.linear <- coxph(PFS5  ~ CHO.pred + ipi.hl, data = metadataCombined)

anova(fit.spline, fit.linear)

## Error in anova(fit.spline, fit.linear):  object 'fit.spline' not found
```

This is not the case wherefore the relationship is modelled linear.

```
CalcHR2(PFS5 ~ CHO.class, data = metadataCombined)

##                N                HR               Pval
##            "690" "2.33 (1.64,3.30)"         "2.05e-06"

CalcHR2(PFS5 ~ C.class, data = metadataCombined)

##                N                HR               Pval
##            "690" "0.95 (0.70,1.30)"            "0.764"

CalcHR2(PFS5 ~ H.class, data = metadataCombined)

##                N                HR               Pval
##            "690" "2.52 (1.77,3.59)"         "2.81e-07"

CalcHR2(PFS5 ~ O.class, data = metadataCombined)

##                N                HR               Pval
##            "690" "2.07 (1.48,2.88)"         "2.04e-05"

CalcHR(PFS5 ~ I(CHO.pred/10), data = metadataCombined)

##                N                HR               Pval
##            "690" "1.22 (1.09,1.36)"         "0.000353"

CalcHR(PFS5 ~ I(C.pred/10), data = metadataCombined)

##                N                HR               Pval
##            "690" "0.97 (0.92,1.02)"            "0.184"

CalcHR(PFS5 ~ I(H.pred/10), data = metadataCombined)

##                N                HR               Pval
##            "690" "1.10 (1.05,1.16)"         "5.53e-05"

CalcHR(PFS5 ~ I(O.pred/10), data = metadataCombined)

##                N                HR               Pval
##            "690" "1.67 (1.38,2.01)"          "9.3e-08"
```

```
CalcHR2(PFS5 ~ CHO.class + ipi.hl, data = metadataCombined)

##                N                HR               Pval
##            "604" "2.30 (1.57,3.37)"         "1.78e-05"

CalcHR2(PFS5 ~ C.class + ipi.hl, data = metadataCombined)

##                N                HR               Pval
##            "604" "0.99 (0.71,1.37)"            "0.949"

CalcHR2(PFS5 ~ H.class + ipi.hl, data = metadataCombined)

##                N                HR               Pval
##            "604" "2.55 (1.74,3.72)"         "1.29e-06"
```

```r
CalcHR2(PFS5 ~ O.class + ipi.hl, data = metadataCombined)

##                    N                  HR                Pval
##               "604" "1.69 (1.19,2.40)"          "0.00325"

CalcHR(PFS5 ~ I(CHO.pred/10) + ipi.hl, data = metadataCombined)

##                    N                  HR                Pval
##               "604" "1.22 (1.09,1.37)"          "0.00091"

CalcHR(PFS5 ~ I(C.pred/10) + ipi.hl, data = metadataCombined)

##                    N                  HR                Pval
##               "604" "0.98 (0.93,1.03)"            "0.438"

CalcHR(PFS5 ~ I(H.pred/10) + ipi.hl, data = metadataCombined)

##                    N                  HR                Pval
##               "604" "1.10 (1.04,1.16)"         "0.000548"

CalcHR(PFS5 ~ I(O.pred/10) + ipi.hl, data = metadataCombined)

##                    N                  HR                Pval
##               "604" "1.59 (1.30,1.93)"         "4.79e-06"
```

9.2 Kaplan Meier Analysis

Patients in each of the clinical cohorts were assigned a probability of being sensitive to each of the three drugs. In this section these probabilities are plotted along with areas determining the three categories sensitive, intermeidate, and resistant. Kaplan Meier curves are also established according to the categorisations and the hazard rate ratios obtained between sensitive:intermediate and sensitive:resistant are estimated. For the cohort Combined this is done in the code chunk below. The resulting plots are shown in Figure 9.1.

```r
addAreaColour <- function(prob, col.code){
  polygon(x = c(0, 0, rep(length(prob), 2)),
          y = c(quantile(prob, 2/3), 1, 1,
                quantile(prob, 2/3)),
          col = paste(col.code[1], 60, sep = ""),
          border = 0)

  polygon(x = c(0, 0, rep(length(prob), 2)),
          y = c(quantile(prob, 1/3),
                quantile(prob, 2/3),
                quantile(prob, 2/3),
                quantile(prob, 1/3)),
          col = paste(col.code[2], 60, sep = ""),,
          border = 0)

  polygon(x = c(0, 0, rep(length(prob), 2)),
          y = c(quantile(prob, 1/3),
                0,0,
                quantile(prob, 1/3)),
          col = paste(col.code[3], 60, sep = ""),,
          border = 0)

  segments(x0=0,y0= quantile(prob, 1/3), x1=length(prob),
           y1 =quantile(prob, 1/3) , col = "white", lwd = 4)
  segments(x0=0,y0= quantile(prob, 2/3), x1=length(prob),
           y1 =quantile(prob, 2/3), col = "white", lwd = 4)
```

```
}
```

```
par(mfrow= c(2, 4))
par(mar = c(5.1, 2.8, 4.1, 1))
par(oma = c(0, 3.1, 0, 0))

drugs <- c("C", "H", "O", "CHO")
names(drugs) <- c("Cyclophosphamide", "Doxorubicin", "Vincristine", "CHO")
for(i in 1:length(drugs)){
  drug <- drugs[i]

  par(mfg = c(1, i))

  prob <- 1-metadataCombined[, paste(drug, "prob", sep = ".")]

  plot(sort(prob), col = 0,   las = 1,
       xlab = "Samples", ylab = "Probablity of sensitivity",
       main = paste("Combined,", names(drug)))

  addAreaColour(prob, col.code)

  points(sort(prob))

  if(i == 1)
    mtext("Probablity of sensitivity",outer = TRUE,side = 2,adj=0.87)

  mtext(paste0("(", letters[i], ")"), 3,
        line=1.5, adj=-0.145, cex=9/pointsize)

  table(metadataCombined[, paste(drug, "class", sep = ".")], useNA ="ifany")

  par(mfg = c(2, i))

  formula <- as.formula(paste("PFS5 ~ ", drug, ".class", sep = ""))
  fit <- survfit(formula, data = metadataCombined)

  plot(fit,
       col  = col.code[
         levels(metadataCombined[, paste(drug, ".class", sep = "")])],
       xlab = "Progression free survival (years)",
       ylab = "Survival probability",
       las = 1,
       main = paste("Combined,", names(drug)), lwd = 1.3)

  legend("bottomleft", lty = 1,
         col = col.code[levels(
           metadataCombined[, paste(drug, ".class", sep = "")])],
         legend = levels(metadataCombined[, paste(drug, "class", sep = ".")]),
         bty = "n")

  cox.fit <- coxph(formula, data = metadataCombined)
  cox.sum <- summary(cox.fit)

  cox.cph <- cph(formula, data = metadataCombined, surv = TRUE,
             x= TRUE, y = TRUE)
  n.risk <- cox.cph $surv.summary[, , "n.risk"]
  axis(side = 3, at = names(n.risk), labels = n.risk)
```

```
  legend("bottomright",
         paste(c("I:S ", "R:S "), c("Hz: ", "Hz: ") ,
               round2(cox.sum$coefficients[,2], 2), #c(" CI ", " CI ") ,
               c(" (", " ("),
               round2(cox.sum$conf.int[,3], 2), c(", ", ", "),
               round2(cox.sum$conf.int[,4], 2),
               c(")", ")"),
               sep = ""),
         bty = "n")

  if(i == 1)
    mtext("Survival probability",outer = TRUE, side = 2, adj =0.18 )

  mtext(paste0("(", letters[i + 3], ")"), 3,
        line=1.5, adj=-0.145, cex=9/pointsize)

}

## Error:  could not find function "cph"
```

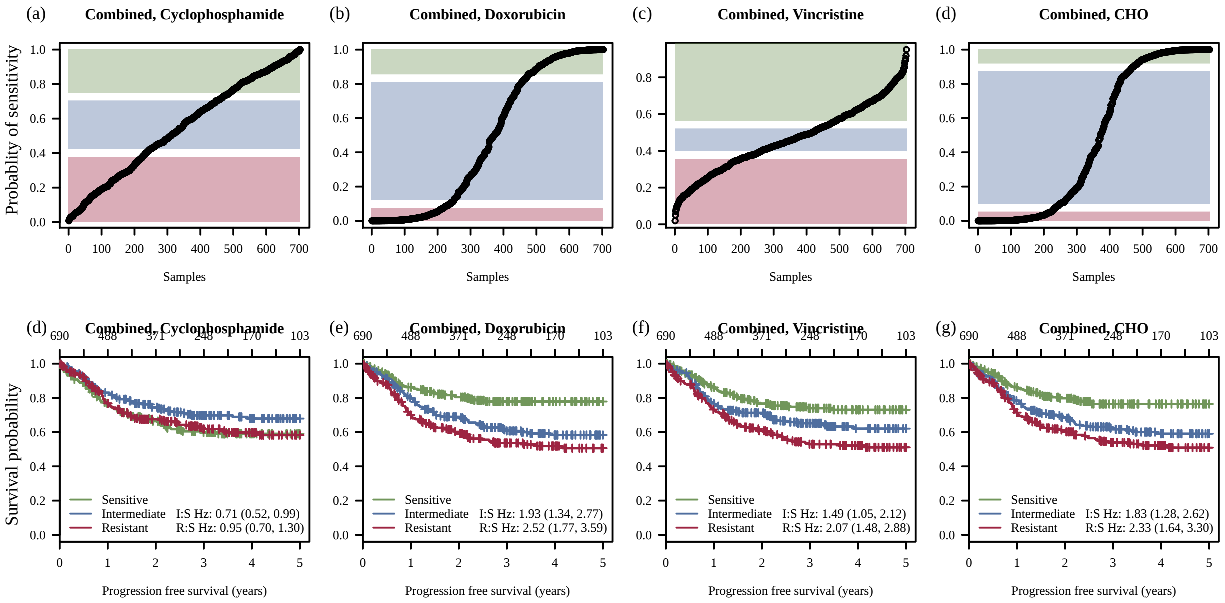

Figure 9.1: The classification of IDRC and LLMPP into sensitive, intermediate, and resistant patients. In panels A, B, C, and D the probability of being sensitive is plotted for each patient. Based on the probabilities the patients are categorised into tertiles with those deemed sensitive intermediate, and resistant indicated by green, blue, and red. Kaplan-Meier curves for PFS are shown in panels E, F, G, and H.

9.3 Cox Proportional Hazards Regression based on Restricted Cubic Splines

In the following code chunk a restricted cubic spline with four knots is fitted to the resistance index. Based on the fitted spline, survival curves, adjusted for IPI are generated. The resulting plot is shown in Figure 9.2.

```
n.knots <- 4
drugs <- c("C.pred", "H.pred", "O.pred", "CHO.pred")
```

```r
par(mfrow = c(2, length(drugs)))
par(mar = c(5.1, 2.8, 4.1, 1))
par(oma = c(0, 3.1, 0, 0))

for(i in 1:length(drugs)){
  drug <- drugs[i]
  drug.i <- metadataCombined[, drug]
  ipi <- as.factor(metadataCombined$ipi.hl)
  d <- datadist(drug.i, ipi)
  options(datadist = "d", width = 150)


  main = paste("Combined,", ifelse(grepl("Combined" , drug), "Combined", drug))

  fit <- cph(metadataCombined$PFS5  ~ rcs(drug.i, n.knots) + ipi, surv = TRUE,
             x= TRUE, y = TRUE)

  seq(sort(drug.i)[11], sort(drug.i)[length(drug.i)- 10], length.out = 5)

  par(mar = c(5.1, 3.5, 4.1, 1))
  #par(oma = c(0,5,0,0))
  par(mfg = c(1,i))
  p <- Predict(fit, drug.i=seq(min(drug.i), max(drug.i), length.out=20),
               ipi = "2-3", #age = median(age),
               fun=exp)

  plot.cv(x = p$drug.i, y = p$yhat, lo = p$lower, up=p$upper,
          main = main,
          las  = 1,
          ylab = "log relative hazard",
          xlab = "Area under dose response curve")

    marks <- seq(sort(drug.i)[11], sort(drug.i)[length(drug.i)- 10], length.out = 5)
  r <- range(c(log(p$lower), log(p$upper)))
  r <- r[2] - r[1]
  arrows(marks, rep(min(log(p$lower) + r/10, 5)), marks, rep(min(log(p$lower), 5)),
         length = 0.05, col = col.data$col)

  if(i == 1)
    mtext("Log relative hazard",outer = TRUE,side = 2,adj=0.87)

  mtext(paste0("(", letters[i], ")"), 3,
        line=1.5, adj=-0.145, cex=9/pointsize)
  par(mfg = c(2, i))

   plot(0,0, ylim = c(0,1), xlim = c(0,5), col = 0,
       xlab = "Overall survival (years)",
       ylab = "Survival probability", las = 1)

  survplot(fit, drug.i, ipi = "2-3",
           col.fill=paste(col.data$col, 50, sep = ""),
           col=col.data$col, lwd = 2,
           lty = 1, label.curves=FALSE,
           add  = TRUE)

  legend("bottomleft", lty = 1, col = col.data$col[1:5], legend = col.data$cell[1:5],
         bty = "n")

  title(main, line=3)
```

```
  n.risk <- fit$surv.summary[, , "n.risk"]
  axis(side=3,at= names(n.risk), labels=n.risk)
  graphics:::box()

  if(i == 1)
    mtext("Survival probability",outer = TRUE, side = 2, adj =0.18 )

  mtext(paste0("(", letters[i], ")"), 3,
        line=1.5, adj=-0.145, cex=9/pointsize)
}

## Error:  could not find function "datadist"
```

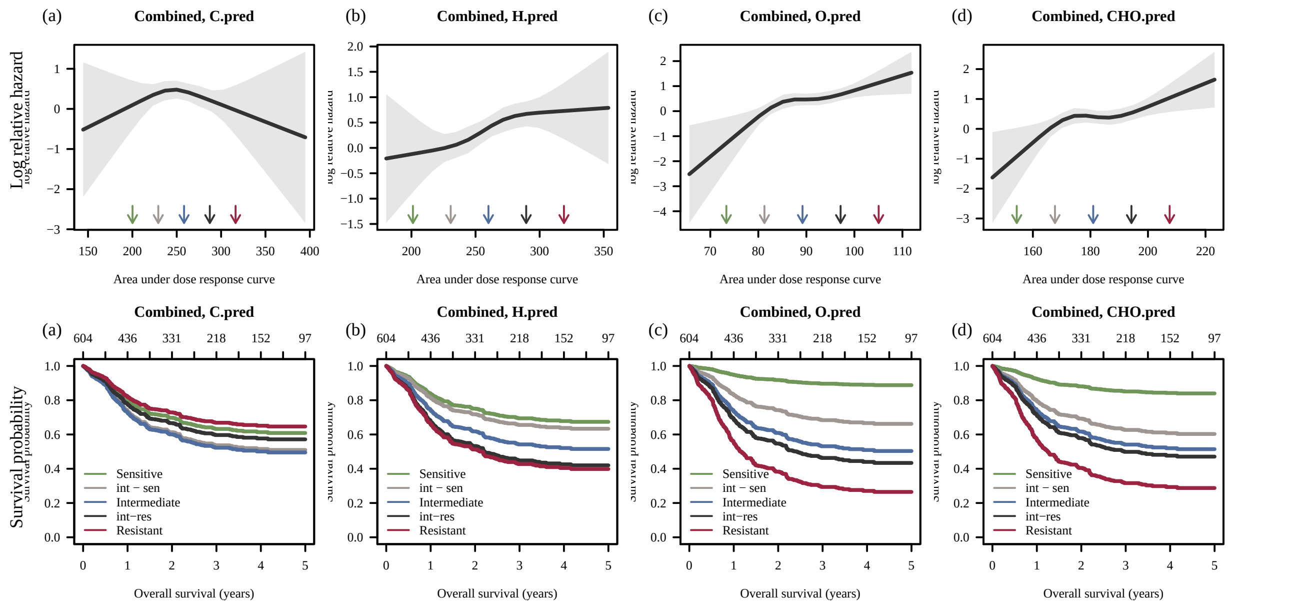

Figure 9.2: Fitted restricted cubic spline to the IDRC and LLMPP cohort. In panels A, B, C, and D the fitted spline is shown. Generated survival curves for PFS are shown in panels E, F, G, and H.

9.4 Comparison of REGS Classifiers and Predictors

The performance of the REGS classifier and predictor for the combination therapy CHO is compared in terms of AUC of the ROC curves. The results are shown in Figure 9.3.

```
# 1) with the Kaplan-Meier estimator for computing the weights (default).
drug  <- "CHO"
times <- seq(0.5, 4.5, 0.5)
ROC.class <- timeROC(T=metadataCombined$PFS5[, 1],
                     delta=metadataCombined$PFS5[, 2],
                     marker = metadataCombined[, "CHO.prob"],
                     cause=1,weighting="marginal",
                     times=times,
                     iid=TRUE)

ROC.pred <- timeROC(T=metadataCombined$PFS5[, 1],
                    delta=metadataCombined$PFS5[, 2],
                    marker = metadataCombined[, "CHO.pred"],
                    cause=1,weighting="marginal",
                    times=times,
                    iid=TRUE)
```

```r
par(mfrow = c(1,2), las = 1)

conf.class <- confint(ROC.class)$CI_AUC/100

conf.pred  <- confint(ROC.pred)$CI_AUC/100

range <- range(conf.class, conf.pred )

plot(0,0, ylim = range, xlim = range(times), col = 0, ylab = "AUC(t)", xlab = "Time (Years)",
     main = "AUC for CHO REGS")

abline(h = 0.5, lty = 5, lwd = 1.3, col = "#33333360")

polygon(x= c(times, rev(times)),
        y = c(conf.class[,1], rev(conf.class[,2])),
        col = paste(col.data$col[1], 50, sep = ""), border = NA)

lines(times, ROC.class$AUC, lwd= 2, col = col.data$col[1])

polygon(x= c(times, rev(times)),
        y = c(conf.pred[,1], rev(conf.pred[,2])),
        col = paste(col.data$col[3], 50, sep = ""), border = NA)
lines(times, ROC.pred$AUC, lwd= 2, col = col.data$col[3])

mtext("(a)", 3,
        line=1.5, adj=-0.145, cex=9/pointsize)

AUC <- ROC.class$AUC[!(is.na(ROC.class$AUC) | is.na(ROC.pred$AUC))] -
  ROC.pred$AUC[!(is.na(ROC.class$AUC) | is.na(ROC.pred$AUC))]
times <- ROC.class$times[!(is.na(ROC.class$AUC) | is.na(ROC.pred$AUC))]

mat.iid <- ROC.class$inference$mat_iid_rep_1 - ROC.pred$inference$mat_iid_rep_1
se <- apply(mat.iid, 2, sd)/sqrt(ROC.class$n)
lower.conf.int <- AUC - se * qnorm(1 - (1 - 0.95)/2)
upper.conf.int <- AUC + se * qnorm(1 - (1 - 0.95)/2)

range <- range(lower.conf.int, upper.conf.int)

plot(0, 0, ylim = range, xlim = range(times), col = 0,
     ylab = expression(paste(Delta, "AUC(t)")),
     xlab = "Time (Years)",
     main = "Difference in AUC")

abline(h = 0, lty = 5, lwd = 1.3, col = "#33333360")

polygon(x= c(times, rev(times)),
        y = c(lower.conf.int, rev(upper.conf.int )),
        col = paste(col.data$col[3], 50, sep = ""), border = NA)

lines(times, AUC, lwd= 1.3, col = col.data$col[3])


mtext("(b)", 3,
        line=1.5, adj=-0.145, cex=9/pointsize)
```

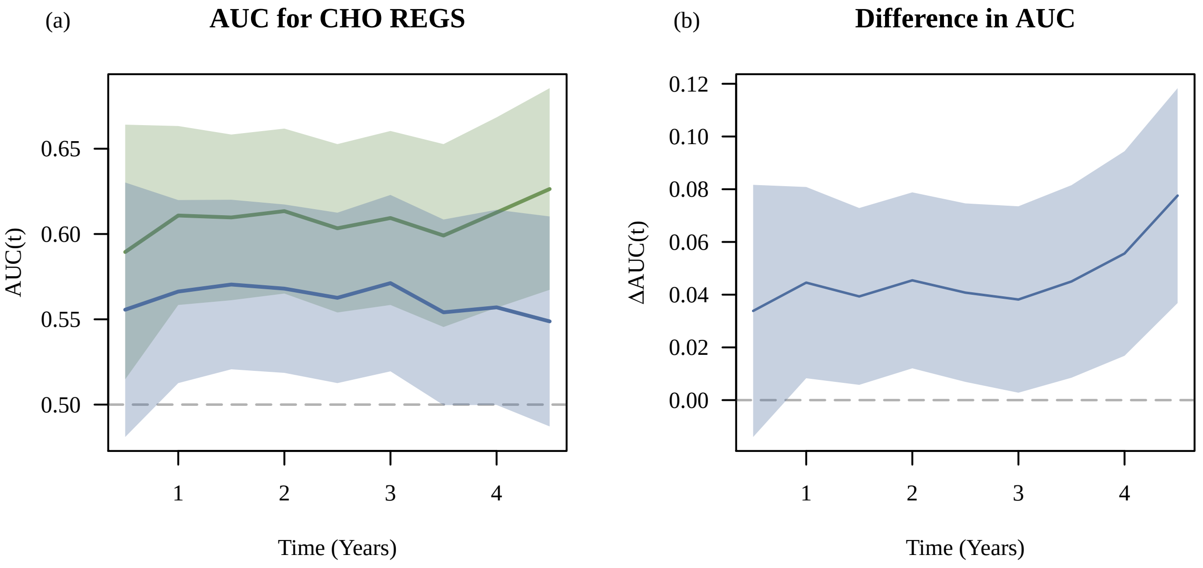

Figure 9.3: Analysis of ROC curves for prediction of the combination therapy CHO. Panel A Shows AUC under the ROC curves plotted against time for the CHO REGS classifier (green) and predictor (blue). Panel B shows the difference in AUC plotted aganst time. All curves are shown with 95% CI.

```
## Error:  could not find function "cph"
```

10 MDFCI Cohort

The MDFCI data consist of data from 67 patients with diffuse large B-cell lymphoma (DLBCL). The data include gene expressions from the Affymetrix GeneChip HG-U133 Plus 2.0 arrays and information about overall survival (OS). The metadata and .CEL files are available at

- `http://www.ncbi.nlm.nih.gov/geo/`

under GEO accession number GSE34171.

10.1 Loading the data

The metadata and RMA normalized GEP data for MDFCI are loaded into R.
The metadata have been constructed by merging the files available at GEO accession number GSE34171 together with Supplementary Table 5 of
Monti, S., Chapuy, B., Takeyama, K., Rodig, S. J., Hao, Y., Yeda, K. T., ... Shipp, M. a. (2012). Integrative analysis reveals an outcome-associated and targetable pattern of p53 and cell cycle deregulation in diffuse large B cell lymphoma. Cancer Cell, 22(3), 359-72.

```
load(file.path(Database,
               "DLBCL/MDFCI_GSE34171/V1/Metadata/metadataMDFCI.RData"))

metadataMDFCI <- metadataMDFCI[!is.na(metadataMDFCI$HGU133Plus2), ]

load(file.path(Database,
               "DLBCL/MDFCI_GSE34171/V1/",
               "PreProcessed/GEPMDFCI_HGU133Plus2_RMA_affy.RData"))
```

The GEP data are probeset wise median centred and scaled to have standard deviation 1.

```
GEPMDFCI.sc <- microarrayScale(GEPMDFCI)
```

10.2 REGS classification

The data is classified according to REGS classifiers for Cyclophosphamide, Doxorubicin, and Vincristine.

```
metadataMDFCI$C.prob <-
  CyclophosphamideClassifier(exprs(GEPMDFCI.sc))[,1]

metadataMDFCI$H.prob <-
  DoxorubicinClassifier(exprs(GEPMDFCI.sc))[,1]

metadataMDFCI$GCPH.prob <-
  GCPDoxorubicinClassifier(exprs(GEPMDFCI.sc))[,1]

metadataMDFCI$O.prob <-
  VincristineClassifier(exprs(GEPMDFCI.sc))[,1]
```

```
test.mat <- metadataMDFCI[, c("C.prob", "H.prob", "O.prob") ]

metadataMDFCI$CHO.prob <-
  apply(test.mat, 1, prod) /
    (apply(test.mat, 1, prod) +
       apply(1-test.mat, 1, prod))
```

Table 10.1: Cox proportional hazard analyses of the association between PFS and OS and the predicted resistance indices for the three drugs. In the multivariate analyses the Cox proportional hazards regressions are adjusted for IPI. The estimated HR's are based on an increase of 10 in the AUC_0

	Overall Survival			Progression Free Survival		
	N	HR (95% CI)	P-value	N	HR (95% CI)	P-value
Cyclophosphamide	67	0.66 (0.23,1.89)	0.441	63	0.85 (0.28,2.53)	0.767
Doxorubicin	67	4.56 (1.29,16.19)	0.0188	63	4.05 (1.13,14.51)	0.0318
Vincristine	67	5.67 (1.24,25.96)	0.0253	63	5.05 (1.08,23.47)	0.039
CHO	67	14.04 (1.82,108.09)	0.0112	63	13.90 (1.80,107.43)	0.0117

```
metadataMDFCI$C.class    <- probCut(metadataMDFCI$C.prob)
metadataMDFCI$H.class    <- probCut(metadataMDFCI$H.prob)
metadataMDFCI$GCPH.class <- probCut(metadataMDFCI$GCPH.prob)
metadataMDFCI$O.class    <- probCut(metadataMDFCI$O.prob)
metadataMDFCI$CHO.class  <- probCut(metadataMDFCI$CHO.prob)
```

10.2.1 Cox Proportional Hazards Regression

The resistance levels assigned to the patients of the MDFCI cohort are investigated by Cox proportional hazards models. First a matrix containing the results are created.

```
cox.MDFCI.prob <- matrix(0, nrow = 4, ncol = 3*2)
rownames(cox.MDFCI.prob) <- c( "Cyclophosphamide", "Doxorubicin", "Vincristine", "CHO")

colnames(cox.MDFCI.prob) <- rep(c("N", "HR (95\\% CI)", "P-value"), 2)
```

Second, the univariate Cox regression analysis is performed.

```
cox.MDFCI.prob[1, 1:3] <- CalcHR2(OS5 ~ C.class,
                          data = metadataMDFCI)
cox.MDFCI.prob[2, 1:3] <- CalcHR2(OS5 ~ H.class,
                          data = metadataMDFCI)
cox.MDFCI.prob[3, 1:3] <- CalcHR2(OS5 ~ O.class,
                          data = metadataMDFCI)
cox.MDFCI.prob[4, 1:3] <- CalcHR2(OS5 ~ CHO.class,
                          data = metadataMDFCI)
```

Third, the multivariate Cox regression analysis is performed.

```
cox.MDFCI.prob[1, 4:6] <- CalcHR2(OS5 ~ C.class + ipi.hl,
                          data = metadataMDFCI)
cox.MDFCI.prob[2, 4:6] <- CalcHR2(OS5 ~ H.class + ipi.hl,
                          data = metadataMDFCI)
cox.MDFCI.prob[3, 4:6] <- CalcHR2(OS5 ~ O.class + ipi.hl,
                          data = metadataMDFCI)
cox.MDFCI.prob[4, 4:6] <- CalcHR2(OS5 ~ CHO.class + ipi.hl,
                          data = metadataMDFCI)
```

The results are shown in Table 10.1.

10.2.2 Kaplan Meier Analysis

Patients in each of the clinical cohorts were assigned a probability of being sensitive to each of the three drugs. In this section these probabilities are plotted along with areas determining the three categories sensitive, intermeidate, and resistant. Kaplan Meier curves are also established according to the categorisations and the hazard rate ratios obtained between sensitive:intermediate and sensitive:resistant are estimated. For the cohort MDFCI this is done in the code chunk below. The resulting plots are shown in Figure 10.1.

```r
addAreaColour <- function(prob, col.code){
  polygon(x = c(0, 0, rep(length(prob), 2)),
          y = c(quantile(prob, 2/3), 1, 1,
                quantile(prob, 2/3)),
          col = paste(col.code[1], 60, sep = ""),
          border = 0)

  polygon(x = c(0, 0, rep(length(prob), 2)),
          y = c(quantile(prob, 1/3),
                quantile(prob, 2/3),
                quantile(prob, 2/3),
                quantile(prob, 1/3)),
          col = paste(col.code[2], 60, sep = ""),,
          border = 0)

  polygon(x = c(0, 0, rep(length(prob), 2)),
          y = c(quantile(prob, 1/3),
                0,0,
                quantile(prob, 1/3)),
          col = paste(col.code[3], 60, sep = ""),,
          border = 0)

  segments(x0=0,y0= quantile(prob, 1/3), x1=length(prob),
           y1 =quantile(prob, 1/3)  , col = "white", lwd = 4)
  segments(x0=0,y0= quantile(prob, 2/3), x1=length(prob),
           y1 =quantile(prob, 2/3), col = "white", lwd = 4)
}
```

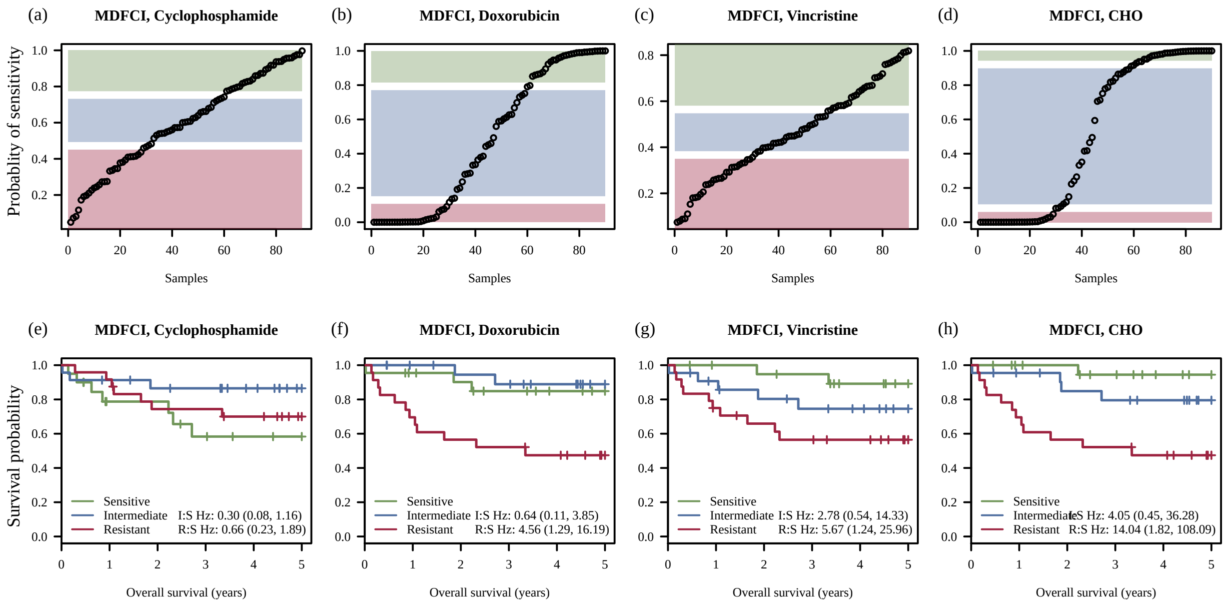

Figure 10.1: The classification of MDFCI into sensitive, intermediate, and resistant patients. In panels A, B, C, and D the probability of being sensitive is plotted for each patient. Based on the probabilities the patients are categorised into tertiles with those deemed sensitive intermediate, and resistant indicated by green, blue, and red. Kaplan-Meier curves for OS are shown in panels E, F, G, and H.

```r
par(mfrow= c(2, 4))
par(mar = c(5.1, 2.8, 4.1, 1))
par(oma = c(0, 3.1, 0, 0))
```

```r
drugs <- c("C", "H", "O", "CHO")
names(drugs) <- c("Cyclophosphamide", "Doxorubicin", "Vincristine", "CHO")
for(i in 1:length(drugs)){
  drug <- drugs[i]

  par(mfg = c(1, i))

  prob <- 1-metadataMDFCI[, paste(drug, "prob", sep = ".")]

  plot(sort(prob), col = 0,   las = 1,
       xlab = "Samples", ylab = "Probablity of sensitivity",
       main = paste("MDFCI,", names(drug)))

  addAreaColour(prob, col.code)

  points(sort(prob))

  if(i == 1)
    mtext("Probablity of sensitivity",outer = TRUE,side = 2,adj=0.87)

  mtext(paste0("(", letters[i], ")"), 3,
        line=1.5, adj=-0.145, cex=9/pointsize)

  table(metadataMDFCI[, paste(drug, "class", sep = ".")], useNA ="ifany")

  par(mfg = c(2, i))

  formula <- as.formula(paste("OS5 ~ ", drug, ".class", sep = ""))
  plot(survfit(formula, data = metadataMDFCI),
       col   = col.code[
         levels(metadataMDFCI[, paste(drug, ".class", sep = "")])],
       xlab = "Overall survival (years)",
       ylab = "Survival probability",
       las = 1,
       main = paste("MDFCI,", names(drug)), lwd = 1.3)

  legend("bottomleft", lty = 1,
         col = col.code[levels(
           metadataMDFCI[, paste(drug, ".class", sep = "")])],
         legend = levels(metadataMDFCI[, paste(drug, "class", sep = ".")]),
         bty = "n")

  cox.fit <- coxph(formula, data = metadataMDFCI)
  cox.sum <- summary(cox.fit)

  legend("bottomright",
         paste(c("I:S ", "R:S "), c("Hz: ", "Hz: ") ,
               round2(cox.sum$coefficients[,2], 2), #c(" CI ", " CI ") ,
               c(" (", " ("),
               round2(cox.sum$conf.int[,3], 2), c(", ", ", "),
               round2(cox.sum$conf.int[,4], 2),
               c(")", ")"),
               sep = ""),
         bty = "n")
  if(i == 1)
    mtext("Survival probability",outer = TRUE, side = 2, adj =0.18 )

  mtext(paste0("(", letters[i + 4], ")"), 3,
        line=1.5, adj=-0.145, cex=9/pointsize)
```

```
}
```

10.3 REGS Predictors

Resistance indices are established according to REGS predictors for Cyclophosphamide, Doxorubicin, and Vincristine.

```
metadataMDFCI$C.pred <-
  CyclophosphamidePredictor(exprs(GEPMDFCI.sc))[,1]

metadataMDFCI$H.pred <-
  DoxorubicinPredictor(exprs(GEPMDFCI.sc))[,1]

metadataMDFCI$GCPH.pred <-
  GCPDoxorubicinPredictor(exprs(GEPMDFCI.sc))[,1]

metadataMDFCI$O.pred <-
  VincristinePredictor(exprs(GEPMDFCI.sc))[,1]
```

The resistance indices are combined using the geometric mean.

```
metadataMDFCI$CHO.pred <-
  apply(metadataMDFCI[, c("C.pred", "H.pred", "O.pred") ], 1, function(x) geomean(x))
```

10.3.1 Cox Proportional Hazards Regression

The resistance levels assigned to the patients of the MDFCI cohort are investigated by Cox proportional hazards models. First a matrix containing the results are created.

```
cox.MDFCI.pred <- matrix(0, nrow = 4, ncol = 3*2)
rownames(cox.MDFCI.pred) <- c( "Cyclophosphamide", "Doxorubicin", "Vincristine", "CHO")

colnames(cox.MDFCI.pred) <- rep(c("N", "HR (95\\% CI)", "P-value"), 2)
```

Second, the univariate Cox regression analysis is performed.

```
cox.MDFCI.pred[1, 1:3] <- CalcHR(OS5 ~ I(C.pred/10),
                                 data = metadataMDFCI)
cox.MDFCI.pred[2, 1:3] <- CalcHR(OS5 ~ I(H.pred/10),
                                 data = metadataMDFCI)
cox.MDFCI.pred[3, 1:3] <- CalcHR(OS5 ~ I(O.pred/10),
                                 data = metadataMDFCI)
cox.MDFCI.pred[4, 1:3] <- CalcHR(OS5 ~ I(CHO.pred/10),
                                 data = metadataMDFCI)
```

Third, the multivariate Cox regression analysis is performed.

```
cox.MDFCI.pred[1, 4:6] <- CalcHR(OS5 ~ I(C.pred/10) + ipi.hl,
                                 data = metadataMDFCI)
cox.MDFCI.pred[2, 4:6] <- CalcHR(OS5 ~ I(H.pred/10) + ipi.hl,
                                 data = metadataMDFCI)
cox.MDFCI.pred[3, 4:6] <- CalcHR(OS5 ~ I(O.pred/10) + ipi.hl,
                                 data = metadataMDFCI)
cox.MDFCI.pred[4, 4:6] <- CalcHR(OS5 ~ I(CHO.pred/10) + ipi.hl,
                                 data = metadataMDFCI)
```

The results are shown in Table 10.2.

Table 10.2: Cox proportional hazard analyses of the association between PFS and OS and the predicted resistance indices for the three drugs. In the multivariate analyses the Cox proportional hazards regressions are adjusted for IPI. The estimated HR's are based on an increase of 10 in the AUC_0

	Univariate			Multivariate		
	N	HR (95% CI)	P-value	N	HR (95% CI)	P-value
Cyclophosphamide	67	1.06 (0.86,1.31)	0.598	63	1.14 (0.90,1.43)	0.284
Doxorubicin	67	1.32 (1.14,1.54)	0.000318	63	1.34 (1.15,1.57)	0.000237
Vincristine	67	2.73 (1.29,5.80)	0.00872	63	2.49 (1.22,5.05)	0.0117
CHO	67	1.91 (1.28,2.87)	0.00166	63	2.03 (1.35,3.04)	0.000612

10.3.2 Cox Proportional Hazards Regression based on Restricted Cubic Splines

In the following code chunk a restricted cubic spline with four knots is fitted to the resistance index. Based on the fitted spline, survival curves, adjusted for IPI are generated. The resulting plot is shown in Figure 10.2.

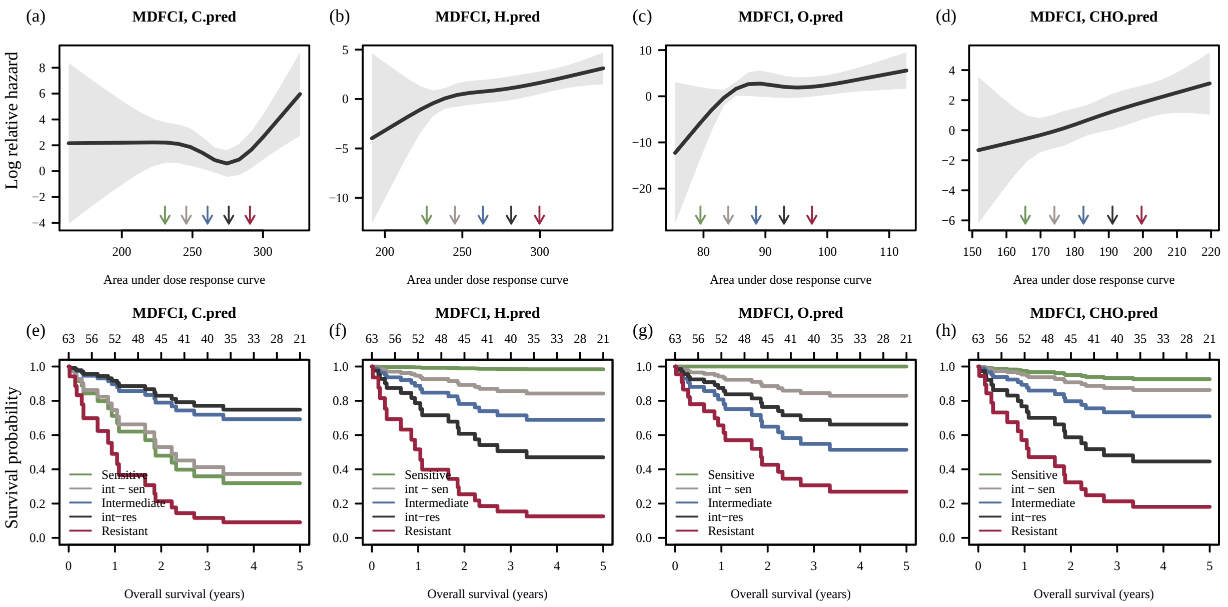

Figure 10.2: Fitted restricted cubic spline to the MDFCI cohort. In panels A, B, C, and D the fitted spline is shown. Generated survival curves for OS are shown in panels E, F, G, and H.

```
n.knots <- 4
drugs <- c("C.pred", "H.pred", "O.pred", "CHO.pred")

par(mfrow = c(2, length(drugs)))
par(mar = c(5.1, 2.8, 4.1, 1))
par(oma = c(0, 3.1, 0, 0))

for(i in 1:length(drugs)){
  drug <- drugs[i]
  drug.i <- metadataMDFCI[, drug]
  ipi <- as.factor(metadataMDFCI$ipi.hl)
  d <- datadist(drug.i, ipi)
  options(datadist = "d", width = 150)


  main = paste("MDFCI,", ifelse(grepl("Combined" , drug), "Combined", drug))

  fit <- cph(metadataMDFCI$OS5  ~ rcs(drug.i, n.knots) + ipi, surv = TRUE,
             x= TRUE, y = TRUE)
```

```r
  seq(sort(drug.i)[11], sort(drug.i)[length(drug.i)- 10], length.out = 5)


  par(mfg = c(1,i))
  p <- Predict(fit, drug.i=seq(min(drug.i), max(drug.i), length.out=20),
               ipi = "2-3", #age = median(age),
               fun=exp)

  plot.cv(x = p$drug.i, y = p$yhat, lo = p$lower, up=p$upper,
          main = main,
          las  = 1,
          ylab = "log relative hazard",
          xlab = "Area under dose response curve")

    marks <- seq(sort(drug.i)[11], sort(drug.i)[length(drug.i)- 10], length.out = 5)
  r <- range(c(log(p$lower), log(p$upper)))
  r <- r[2] - r[1]
  arrows(marks, rep(min(log(p$lower) + r/10, 5)), marks, rep(min(log(p$lower), 5)),
         length = 0.05, col = col.data$col)

  if(i == 1)
    mtext("Log relative hazard",outer = TRUE,side = 2,adj=0.87)

  mtext(paste0("(", letters[i], ")"), 3,
        line=1.5, adj=-0.145, cex=9/pointsize)
  par(mfg = c(2, i))

   plot(0,0, ylim = c(0,1), xlim = c(0,5), col = 0,
       xlab = "Overall survival (years)",
       ylab = "Survival probability", las = 1)

  survplot(fit, drug.i, ipi = "2-3",
           col.fill=paste(col.data$col, 50, sep = ""),
           col=col.data$col, lwd = 2,
           lty = 1, label.curves=FALSE,
           add  = TRUE)

  legend("bottomleft", lty = 1, col = col.data$col[1:5], legend = col.data$cell[1:5],
         bty = "n")

  title(main, line=3)
  n.risk <- fit$surv.summary[, , "n.risk"]
  axis(side=3,at= names(n.risk), labels=n.risk)
  graphics:::box()

  if(i == 1)
    mtext("Survival probability",outer = TRUE, side = 2, adj =0.18 )

  mtext(paste0("(", letters[i + 4], ")"), 3,
        line=1.5, adj=-0.145, cex=9/pointsize)
}

## Error:  could not find function "datadist"
```

10.4 Comparison of REGS Classifiers and Predictors

The performance of the REGS classifier and predictor for the combination therapy CHO is compared in terms of AUC of the ROC curves. The results are shown in Figure 10.3.

```
# 1) with the Kaplan-Meier estimator for computing the weights (default).
drug  <- "CHO"
times <- seq(0.5, 4.5, 0.5)
ROC.class <- timeROC(T=metadataMDFCI$OS5[, 1],
                     delta=metadataMDFCI$OS5[, 2],
                     marker = metadataMDFCI[, "CHO.prob"],
                     cause=1,weighting="marginal",
                     times=times,
                     iid=TRUE)

ROC.pred <- timeROC(T=metadataMDFCI$OS5[, 1],
                    delta=metadataMDFCI$OS5[, 2],
                    marker = metadataMDFCI[, "CHO.pred"],
                    cause=1,weighting="marginal",
                    times=times,
                    iid=TRUE)

par(mfrow = c(1,2), las = 1)
conf.class <- confint(ROC.class)$CI_AUC/100
conf.pred  <- confint(ROC.pred)$CI_AUC/100

range <- range(conf.class, conf.pred )

plot(0,0, ylim = range, xlim = range(times), col = 0, ylab = "AUC(t)", xlab = "Time (Years)",
     main = "AUC for CHO REGS")

abline(h = 0.5, lty = 5, lwd = 1.3, col = "#33333360")

polygon(x= c(times, rev(times)),
        y = c(conf.class[,1], rev(conf.class[,2])),
        col = paste(col.data$col[1], 50, sep = ""), border = NA)

lines(times, ROC.class$AUC, lwd= 2, col = col.data$col[1])

polygon(x= c(times, rev(times)),
        y = c(conf.pred[,1], rev(conf.pred[,2])),
        col = paste(col.data$col[3], 50, sep = ""), border = NA)
lines(times, ROC.pred$AUC, lwd= 2, col = col.data$col[3])

mtext("(a)", 3,
        line=1.5, adj=-0.145, cex=9/pointsize)

AUC <- ROC.class$AUC[!(is.na(ROC.class$AUC) | is.na(ROC.pred$AUC))] -
  ROC.pred$AUC[!(is.na(ROC.class$AUC) | is.na(ROC.pred$AUC))]
times <- ROC.class$times[!(is.na(ROC.class$AUC) | is.na(ROC.pred$AUC))]

mat.iid <- ROC.class$inference$mat_iid_rep_1 - ROC.pred$inference$mat_iid_rep_1
se <- apply(mat.iid, 2, sd)/sqrt(ROC.class$n)
lower.conf.int <- AUC - se * qnorm(1 - (1 - 0.95)/2)
upper.conf.int <- AUC + se * qnorm(1 - (1 - 0.95)/2)

range <- range(lower.conf.int, upper.conf.int)

plot(0, 0, ylim = range, xlim = range(times), col = 0,
```

```
     ylab = expression(paste(Delta, "AUC(t)")),
     xlab = "Time (Years)",
     main = "Difference in AUC")

abline(h = 0, lty = 5, lwd = 1.3, col = "#33333360")

polygon(x= c(times, rev(times)),
        y = c(lower.conf.int, rev(upper.conf.int )),
        col = paste(col.data$col[3], 50, sep = ""), border = NA)

lines(times, AUC, lwd= 1.3, col = col.data$col[3])


mtext("(b)", 3,
        line=1.5, adj=-0.145, cex=9/pointsize)
```

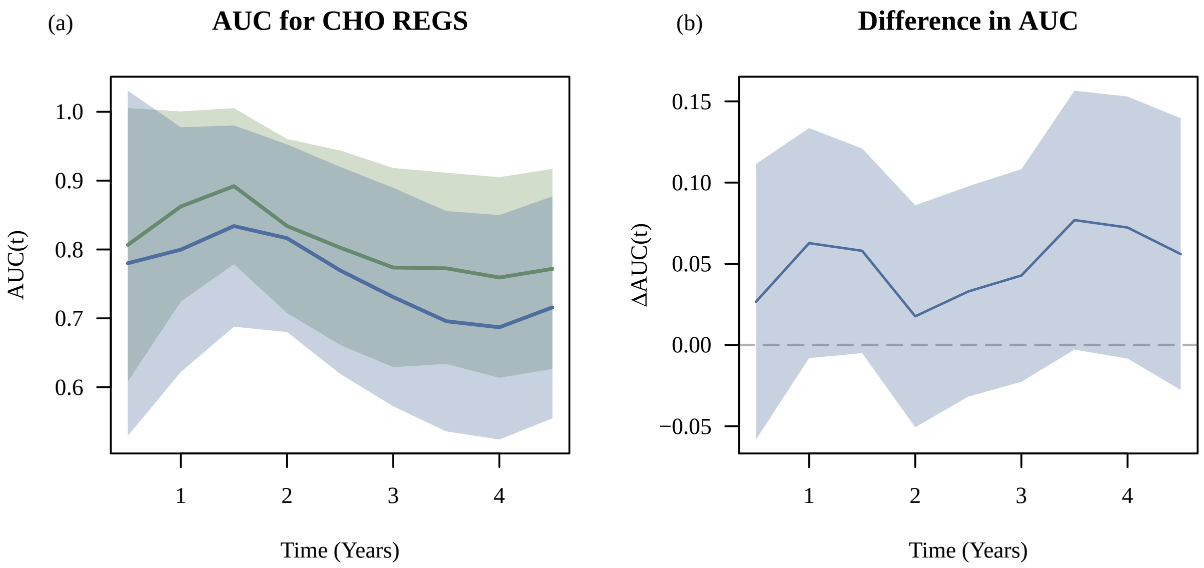

Figure 10.3: Analysis of ROC curves for prediction of the combination therapy CHO. Panel A Shows AUC under the ROC curves plotted against time for the CHO REGS classifier (green) and predictor (blue). Panel B shows the difference in AUC plotted aganst time. All curves are shown with 95% CI.

```
## Error:  could not find function "cph"
```

11 UAMS Cohort

The *UAMS* data consist of data from 565 patients with multiple myeloma (MM). The data include gene expressions from a HG-U133Plus 2.0 array platform and information about overall survival (OS) and event free survival (EFS). The metadata and .CEL files are available at

- `http://www.ncbi.nlm.nih.gov/geo/`

under GEO accession number GSE24080.

11.1 Loading the data

The metadata and RMA normalized GEP data for IDRC RCHOP are loaded into R.

```
load(file.path(Database,
               "MM/UAMS_GSE24080/V1/Metadata/metadataUAMS.RData"))

load(file.path(Database,
               "MM/UAMS_GSE24080/V1",
               "PreProcessed/GEPUAMS_RMA_affy.RData"))
```

The GEP data are probeset wise median centred and scaled to have standard deviation 1.

```
GEPUAMS.sc <- microarrayScale(GEPUAMS)
```

11.2 REGS classification

The data is classified according to REGS classifiers for Rituximab, Cyclophosphamide, Doxorubicin, and Vincristine.

```
metadataUAMS$C.prob <-
  CyclophosphamideClassifier(exprs(GEPUAMS.sc))[,1]

metadataUAMS$H.prob <-
  DoxorubicinClassifier(exprs(GEPUAMS.sc))[,1]

metadataUAMS$GCPH.prob <-
  GCPDoxorubicinClassifier(exprs(GEPUAMS.sc))[,1]

metadataUAMS$O.prob <-
  VincristineClassifier(exprs(GEPUAMS.sc))[,1]
```

```
test.mat <- metadataUAMS[, c("C.prob", "H.prob", "O.prob") ]

metadataUAMS$CHO.prob <-
  apply(test.mat, 1, prod) /
    (apply(test.mat, 1, prod) +
       apply(1-test.mat, 1, prod))
```

```
metadataUAMS$C.class <- probCut(metadataUAMS$C.prob)
metadataUAMS$H.class <- probCut(metadataUAMS$H.prob)
metadataUAMS$O.class <- probCut(metadataUAMS$O.prob)
metadataUAMS$CHO.class <- probCut(metadataUAMS$CHO.prob)
metadataUAMS$GCPH.class <- probCut(metadataUAMS$GCPH.prob)
```

Table 11.1: Cox proportional hazard analyses of the association between PFS and OS and the predicted resistance indices for the three drugs. In the multivariate analyses the Cox proportional hazards regressions are adjusted for IPI. The estimated HR's are based on an increase of 10 in the AUC_0

	Overall Survival			Progression Free Survival		
	N	HR (95% CI)	P-value	N	HR (95% CI)	P-value
Cyclophosphamide	559	0.70 (0.47,1.06)	0.093	559	0.92 (0.63,1.36)	0.69
Doxorubicin	559	1.10 (0.74,1.62)	0.642	559	0.91 (0.62,1.32)	0.607
Vincristine	559	0.89 (0.59,1.35)	0.594	559	0.81 (0.55,1.20)	0.295
CHO	559	0.90 (0.61,1.33)	0.599	559	0.77 (0.53,1.13)	0.178

11.2.1 Cox Proportional Hazards Regression

The resistance levels assigned to the patients of the MDFCI cohort are investigated by Cox proportional hazards models. First a matrix containing the results are created.

```
cox.UAMS.prob <- matrix(0, nrow = 4, ncol = 3*2)
rownames(cox.UAMS.prob) <- c( "Cyclophosphamide", "Doxorubicin", "Vincristine", "CHO")

colnames(cox.UAMS.prob) <- rep(c("N", "HR (95\\% CI)", "P-value"), 2)
```

Second, the univariate Cox regression analysis is performed for both OS and PFS.

```
cox.UAMS.prob[1, 1:3] <- CalcHR2(PFS5 ~ C.class,
                          data = metadataUAMS)
cox.UAMS.prob[2, 1:3] <- CalcHR2(PFS5 ~ H.class,
                          data = metadataUAMS)
cox.UAMS.prob[3, 1:3] <- CalcHR2(PFS5 ~ O.class,
                          data = metadataUAMS)
cox.UAMS.prob[4, 1:3] <- CalcHR2(PFS5 ~ CHO.class,
                          data = metadataUAMS)

cox.UAMS.prob[1, 4:6] <- CalcHR2(OS5 ~ C.class,
                          data = metadataUAMS)
cox.UAMS.prob[2, 4:6] <- CalcHR2(OS5 ~ H.class,
                          data = metadataUAMS)
cox.UAMS.prob[3, 4:6] <- CalcHR2(OS5 ~ O.class,
                          data = metadataUAMS)
cox.UAMS.prob[4, 4:6] <- CalcHR2(OS5 ~ CHO.class,
                          data = metadataUAMS)
```

The results are shown in Table 11.1.

11.2.2 Kaplan Meier Analysis

Patients in each of the clinical cohorts were assigned a probability of being sensitive to each of the three drugs. In this section these probabilities are plotted along with areas determining the three categories sensitive, intermeidate, and resistant. Kaplan Meier curves are also established according to the categorisations and the hazard rate ratios obtained between sensitive:intermediate and sensitive:resistant are estimated. For the cohort UAMS this is done in the code chunk below. The resulting plots are shown in Figure 11.1.

```
addAreaColour <- function(prob, col.code){
  polygon(x = c(0, 0, rep(length(prob), 2)),
          y = c(quantile(prob, 2/3), 1, 1,
                quantile(prob, 2/3)),
          col = paste(col.code[1], 60, sep = ""),
          border = 0)

  polygon(x = c(0, 0, rep(length(prob), 2)),
```

```
          y = c(quantile(prob, 1/3),
                quantile(prob, 2/3),
                quantile(prob, 2/3),
                quantile(prob, 1/3)),
          col = paste(col.code[2], 60, sep = ""),,
          border = 0)

  polygon(x = c(0, 0, rep(length(prob), 2)),
          y = c(quantile(prob, 1/3),
                0,0,
                quantile(prob, 1/3)),
          col = paste(col.code[3], 60, sep = ""),,
          border = 0)

  segments(x0=0,y0= quantile(prob, 1/3), x1=length(prob),
           y1 =quantile(prob, 1/3) , col = "white", lwd = 4)
  segments(x0=0,y0= quantile(prob, 2/3), x1=length(prob),
           y1 =quantile(prob, 2/3), col = "white", lwd = 4)
}
```

```
par(mfrow= c(2, 4))
par(mar = c(5.1, 2.8, 4.1, 1))
par(oma = c(0, 3.1, 0, 0))

drugs <- c("C", "H", "O", "CHO")
names(drugs) <- c("Cyclophosphamide", "Doxorubicin", "Vincristine", "CHO")
for(i in 1:length(drugs)){
  drug <- drugs[i]

  par(mfg = c(1, i))

  prob <- 1-metadataUAMS[, paste(drug, "prob", sep = ".")]

  plot(sort(prob), col = 0,   las = 1,
       xlab = "Samples", ylab = "Probablity of sensitivity",
       main = paste("UAMS,", names(drug)))

  addAreaColour(prob, col.code)

  points(sort(prob))

  if(i == 1)
    mtext("Probablity of sensitivity",outer = TRUE,side = 2,adj=0.87)

  mtext(paste0("(", letters[i], ")"), 3,
        line=1.5, adj=-0.145, cex=9/pointsize)

  table(metadataUAMS[, paste(drug, "class", sep = ".")], useNA ="ifany")

  par(mfg = c(2, i))

  formula <- as.formula(paste("OS5 ~ ", drug, ".class", sep = ""))
  plot(survfit(formula, data = metadataUAMS),
       col   = col.code[
         levels(metadataUAMS[, paste(drug, ".class", sep = "")])],
       xlab = "Overall survival (years)",
       ylab = "Survival probability",
       las = 1,
```

```
      main = paste("UAMS,", names(drug)), lwd = 1.3)

  legend("bottomleft", lty = 1,
         col = col.code[levels(
           metadataUAMS[, paste(drug, ".class", sep = "")])],
         legend = levels(metadataUAMS[, paste(drug, "class", sep = ".")]),
         bty = "n")

  cox.fit <- coxph(formula, data = metadataUAMS)
  cox.sum <- summary(cox.fit)

  legend("bottomright",
         paste(c("I:S ", "R:S "), c("Hz: ", "Hz: ") ,
               round2(cox.sum$coefficients[,2], 2), #c(" CI ", " CI ") ,
               c(" (", " ("),
               round2(cox.sum$conf.int[,3], 2), c(", ", ", "),
               round2(cox.sum$conf.int[,4], 2),
               c(")", ")"),
               sep = ""),
         bty = "n")

  if(i == 1)
    mtext("Survival probability",outer = TRUE, side = 2, adj =0.18 )

  mtext(paste0("(", letters[i + 4], ")"), 3,
        line=1.5, adj=-0.145, cex=9/pointsize)

}
```

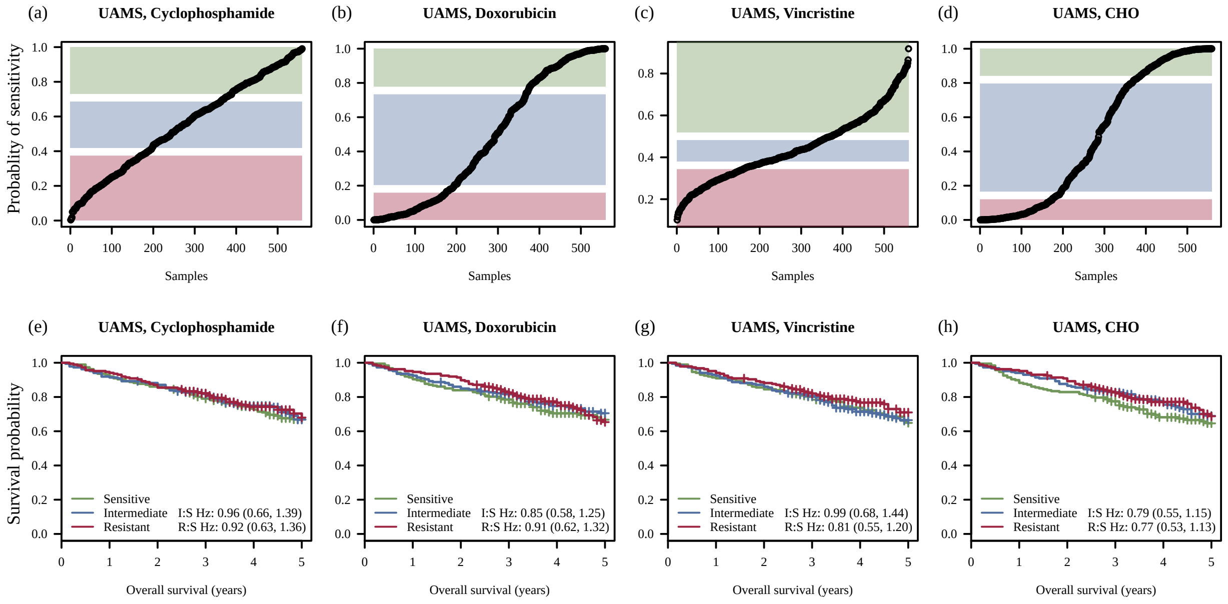

Figure 11.1: The classification of UAMS into sensitive, intermediate, and resistant patients. In panels A, B, C, and D the probability of being sensitive is plotted for each patient. Based on the probabilities the patients are categorised into tertiles with those deemed sensitive intermediate, and resistant indicated by green, blue, and red. Kaplan-Meier curves for OS are shown in panels E, F, G, and H.

11.3 REGS Predictors

Resistance indices are established according to REGS predictors for Cyclophosphamide, Doxorubicin, Vincristine.

```
metadataUAMS$C.pred <-
  CyclophosphamidePredictor(exprs(GEPUAMS.sc))[,1]

metadataUAMS$H.pred <-
  DoxorubicinPredictor(exprs(GEPUAMS.sc))[,1]

metadataUAMS$GCPH.pred <-
  GCPDoxorubicinPredictor(exprs(GEPUAMS.sc))[,1]

metadataUAMS$O.pred <-
  VincristinePredictor(exprs(GEPUAMS.sc))[,1]
```

The resistance indices are combined using the geometric mean.

```
metadataUAMS$CHO.pred <- apply(metadataUAMS[, c("C.pred", "H.pred", "O.pred") ], 1, function(x) mean(x))
```

11.3.1 Cox Proportional Hazards Regression

The resistance levels assigned to the patients of the UAMS cohort are investigated by Cox proportional hazards models. First a matrix containing the results are created.

```
cox.UAMS.pred <- matrix(0, nrow = 4, ncol = 3*2)
rownames(cox.UAMS.pred) <- c( "Cyclophosphamide", "Doxorubicin", "Vincristine", "CHO")

colnames(cox.UAMS.pred) <- rep(c("N", "HR (95\\% CI)", "P-value"), 2)
```

Second, the univariate Cox regression analysis is performed for PFS and OS.

```
cox.UAMS.pred[1, 1:3] <- CalcHR(PFS5 ~ I(C.pred/10),
                          data = metadataUAMS)
cox.UAMS.pred[2, 1:3] <- CalcHR(PFS5 ~ I(H.pred/10),
                          data = metadataUAMS)
cox.UAMS.pred[3, 1:3] <- CalcHR(PFS5 ~ I(O.pred/10),
                          data = metadataUAMS)
cox.UAMS.pred[4, 1:3] <- CalcHR(PFS5 ~ I(CHO.pred/10),
                          data = metadataUAMS)

cox.UAMS.pred[1, 4:6] <- CalcHR(OS5 ~ I(C.pred/10),
                          data = metadataUAMS)
cox.UAMS.pred[2, 4:6] <- CalcHR(OS5 ~ I(H.pred/10),
                          data = metadataUAMS)
cox.UAMS.pred[3, 4:6] <- CalcHR(OS5 ~ I(O.pred/10),
                          data = metadataUAMS)
cox.UAMS.pred[4, 4:6] <- CalcHR(OS5 ~ I(CHO.pred/10),
                          data = metadataUAMS)
```

The results are shown in Table 11.2.

11.3.2 Cox Proportional Hazards Regression based on Restricted Cubic Splines

In the following code chunk a restricted cubic spline with four knots is fitted to the resistance index. Based on the fitted spline, survival curves are generated. The resulting plot is shown in Figure 11.2.

Table 11.2: Cox proportional hazard analyses of the association between PFS and OS and the predicted resistance indices for the three drugs. In the multivariate analyses the Cox proportional hazards regressions are adjusted for IPI. The estimated HR's are based on an increase of 10 in the AUC_0

	Overall Survival			Progression Free Survival		
	N	HR (95% CI)	P-value	N	HR (95% CI)	P-value
Cyclophosphamide	559	1.03 (0.97,1.10)	0.289	559	1.08 (1.02,1.14)	0.00865
Doxorubicin	559	1.02 (0.95,1.10)	0.633	559	0.96 (0.90,1.03)	0.286
Vincristine	559	1.23 (0.95,1.58)	0.115	559	1.05 (0.82,1.35)	0.687
CHO	559	1.09 (0.96,1.25)	0.189	559	1.09 (0.96,1.23)	0.19

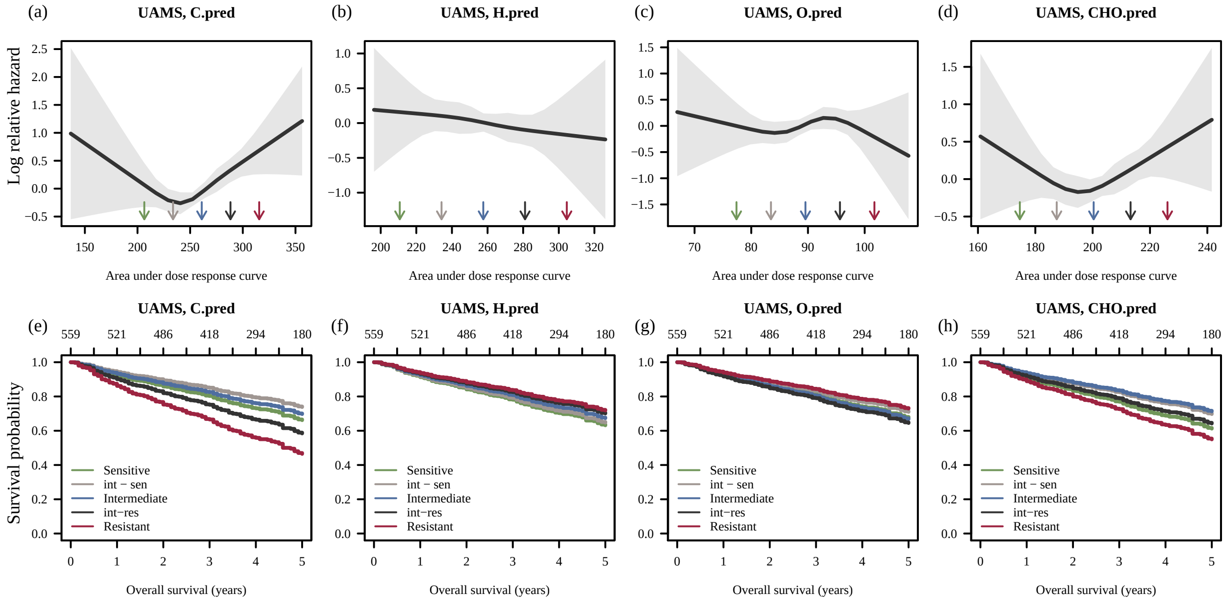

Figure 11.2: Fitted restricted cubic spline to the UAMS cohort. In panels A, B, C, and D the fitted spline is shown. Generated survival curves for OS are shown in panels E, F, G, and H.

```
n.knots <- 4
drugs <- c("C.pred", "H.pred", "O.pred", "CHO.pred")

par(mfrow = c(2, length(drugs)))
par(mar = c(5.1, 2.8, 4.1, 1))
par(oma = c(0, 3.1, 0, 0))

for(i in 1:length(drugs)){
  drug <- drugs[i]
  drug.i <- metadataUAMS[, drug]
  d <- datadist(drug.i)
  options(datadist = "d", width = 150)


  main = paste("UAMS,", ifelse(grepl("Combined" , drug), "Combined", drug))

  fit <- cph(metadataUAMS$OS5  ~ rcs(drug.i, n.knots), surv = TRUE,
           x= TRUE, y = TRUE)

  seq(sort(drug.i)[11], sort(drug.i)[length(drug.i)- 10], length.out = 5)


  par(mfg = c(1,i))
  p <- Predict(fit, drug.i=seq(min(drug.i), max(drug.i), length.out=20),
```

```
               fun=exp)

  plot.cv(x = p$drug.i, y = p$yhat, lo = p$lower, up=p$upper,
          main = main,
          las  = 1,
          ylab = "log relative hazard",
          xlab = "Area under dose response curve")

    marks <- seq(sort(drug.i)[11], sort(drug.i)[length(drug.i)- 10], length.out = 5)
  r <- range(c(log(p$lower), log(p$upper)))
  r <- r[2] - r[1]
  arrows(marks, rep(min(log(p$lower) + r/10, 5)), marks, rep(min(log(p$lower), 5)),
         length = 0.05, col = col.data$col)

  if(i == 1)
    mtext("Log relative hazard",outer = TRUE,side = 2,adj=0.87)

  mtext(paste0("(", letters[i], ")"), 3,
        line=1.5, adj=-0.145, cex=9/pointsize)
  par(mfg = c(2, i))

   plot(0,0, ylim = c(0,1), xlim = c(0,5), col = 0,
       xlab = "Overall survival (years)",
       ylab = "Survival probability", las = 1)

  survplot(fit, drug.i,
           col.fill=paste(col.data$col, 50, sep = ""),
           col=col.data$col, lwd = 2,
           lty = 1, label.curves=FALSE,
           add   = TRUE)

  legend("bottomleft", lty = 1, col = col.data$col[1:5], legend = col.data$cell[1:5],
         bty = "n")

  title(main, line=3)
  n.risk <- fit$surv.summary[, , "n.risk"]
  axis(side=3,at= names(n.risk), labels=n.risk)
  graphics:::box()

  if(i == 1)
    mtext("Survival probability",outer = TRUE, side = 2, adj =0.18 )

  mtext(paste0("(", letters[i + 4], ")"), 3,
        line=1.5, adj=-0.145, cex=9/pointsize)
}

## Error:  could not find function "datadist"
```

11.4 Comparison of REGS Classifiers and Predictors

```
# 1) with the Kaplan-Meier estimator for computing the weights (default).
drug  <- "CHO"
times <- seq(0.5, 4.5, 0.5)
ROC.class <- timeROC(T=metadataUAMS$PFS5[, 1],
                     delta=metadataUAMS$PFS5[, 2],
                     marker = metadataUAMS[, "CHO.prob"],
                     cause=1,weighting="marginal",
```

```r
                     times=times,
                     iid=TRUE)

ROC.pred <- timeROC(T=metadataUAMS$PFS5[, 1],
                    delta=metadataUAMS$PFS5[, 2],
                    marker = metadataUAMS[, "CHO.pred"],
                    cause=1,weighting="marginal",
                    times=times,
                    iid=TRUE)

pdf(file.path(figure.output,"UAMS_AUC.pdf"),
    width = twocol, height = twocol / 2, pointsize = 8)

par(mfrow = c(1,2), las = 1)
conf.class <- confint(ROC.class)$CI_AUC/100
conf.pred  <- confint(ROC.pred)$CI_AUC/100

ROC.pred$AUC
```

```
##             t=0.5               t=1             t=1.5               t=2
## 0.460873050346084 0.559236136652846 0.572227251738238 0.596836172148973
##             t=2.5               t=3             t=3.5               t=4
## 0.551259944944461 0.538185858701772 0.521305351610128 0.536592676795827
##             t=4.5
## 0.521134200101854
```

```r
range <- range(conf.class, conf.pred )

plot(0,0, ylim = range, xlim = range(times), col = 0, ylab = "AUC(t)", xlab = "Time (Years)",
     main = "AUC for CHO REGS")

abline(h = 0.5, lty = 5, lwd = 1.3, col = "#33333360")

polygon(x= c(times, rev(times)),
        y = c(conf.class[,1], rev(conf.class[,2])),
        col = paste(col.data$col[1], 50, sep = ""), border = NA)

lines(times, ROC.class$AUC, lwd= 2, col = col.data$col[1])

polygon(x= c(times, rev(times)),
        y = c(conf.pred[,1], rev(conf.pred[,2])),
        col = paste(col.data$col[3], 50, sep = ""), border = NA)
lines(times, ROC.pred$AUC, lwd= 2, col = col.data$col[3])

mtext("(a)", 3,
        line=1.5, adj=-0.145, cex=9/pointsize)

AUC <- ROC.class$AUC[!(is.na(ROC.class$AUC) | is.na(ROC.pred$AUC))] -
  ROC.pred$AUC[!(is.na(ROC.class$AUC) | is.na(ROC.pred$AUC))]
times <- ROC.class$times[!(is.na(ROC.class$AUC) | is.na(ROC.pred$AUC))]

mat.iid <- ROC.class$inference$mat_iid_rep_1 - ROC.pred$inference$mat_iid_rep_1
se <- apply(mat.iid, 2, sd)/sqrt(ROC.class$n)
lower.conf.int <- AUC - se * qnorm(1 - (1 - 0.95)/2)
upper.conf.int <- AUC + se * qnorm(1 - (1 - 0.95)/2)

range <- range(lower.conf.int, upper.conf.int)

plot(0, 0, ylim = range, xlim = range(times), col = 0,
     ylab = expression(paste(Delta, "AUC(t)")),
```

```
     xlab = "Time (Years)",
     main = "Difference in AUC")

abline(h = 0, lty = 5, lwd = 1.3, col = "#33333360")

polygon(x= c(times, rev(times)),
        y = c(lower.conf.int, rev(upper.conf.int )),
        col = paste(col.data$col[3], 50, sep = ""), border = NA)

lines(times, AUC, lwd= 1.3, col = col.data$col[3])


mtext("(b)", 3,
        line=1.5, adj=-0.145, cex=9/pointsize)
dev.off()

## pdf
##   2
```

```
## Error:  could not find function "cph"
```

12 Comparison of GCP and HBCCL

Comparison of predictors and classifiers for doxorubicin based on GCP and HBCCL.

```
intersect(HGU133Plus2[names(Doxorubicin.reisitance.pred.coef), c("Gene.Symbol"),],
HGU133Plus2[names(GCPDoxorubicin.reisitance.pred.coef), c("Gene.Symbol"),])

## [1] NA       "CDKN2A" "DPYD"   "GLUL"   "NRN1"   "TIMP2"  "VIM"

intersect(HGU133Plus2[names(Doxorubicin.reisitance.class.coef), c("Gene.Symbol"),],
HGU133Plus2[names(GCPDoxorubicin.reisitance.class.coef), c("Gene.Symbol"),])

## [1] NA       "CDKN2A" "KCTD12" "DPYD"   "MEST"   "TIMP2"  "VIM"    "RIMS3"
```

12.1 Cox Proportional Hazards Regression

First, the results are estimated for the REGS classifiers. A matrix for storing results is created.

```
mat1 <- matrix(NA, nrow = 3, ncol = 3*2)
row.names(mat1) <- c("IDRC (PFS)", "LLMPP (PFS)", "MDFCI (OS)")

colnames(mat1) <- rep(c("N", "HR (95\\% CI)", "P-value"), 2)
```

The Cox proportional hazards regression is conducted and the results are added to the matrix mat1

```
mat1[1, 1:3] <- CalcHR2(PFS5 ~ H.class, data = metadataIDRC)
mat1[1, 4:6] <- CalcHR2(PFS5 ~ GCPH.class, data = metadataIDRC)

mat1[2, 1:3] <- CalcHR2(PFS5 ~ H.class, data = metadataLLMPPRCHOP)
mat1[2, 4:6] <- CalcHR2(PFS5 ~ GCPH.class, data = metadataLLMPPRCHOP)

mat1[3, 1:3] <- CalcHR2(OS5 ~ H.class, data = metadataMDFCI)
mat1[3, 4:6] <- CalcHR2(OS5 ~ GCPH.class, data = metadataMDFCI)

mat1

##             N     HR (95\\% CI)       P-value    N     HR (95\\% CI)
## IDRC (PFS)  "470" "2.58 (1.72,3.86)"  "4.37e-06" "470" "1.54 (1.07,2.22)"
## LLMPP (PFS) "220" "2.28 (1.10,4.73)"  "0.0269"   "220" "1.70 (0.89,3.22)"
## MDFCI (OS)  "67"  "4.56 (1.29,16.19)" "0.0188"   "67"  "0.93 (0.28,3.04)"
##             P-value
## IDRC (PFS)  "0.0211"
## LLMPP (PFS) "0.105"
## MDFCI (OS)  "0.899"
```

Second, the results are estimated for the REGS predictors. A matrix for storing results is created.

```
mat2 <- matrix(NA, nrow = 3, ncol = 3*2)
row.names(mat2) <- c("IDRC (PFS)", "LLMPP (PFS)", "MDFCI (OS)")

colnames(mat2) <- rep(c("N", "HR (95\\% CI)", "P-value"), 2)
```

In order to compare the results, the resistance instances are converted to the same scale.

```
metadataIDRC$GCPH.pred2 <- metadataIDRC$GCPH.pred /
  sd(metadataIDRC$GCPH.pred) * sd(metadataIDRC$H.pred)

metadataLLMPPRCHOP$GCPH.pred2 <- metadataLLMPPRCHOP$GCPH.pred /
```

```r
  sd(metadataLLMPPRCHOP$GCPH.pred) * sd(metadataLLMPPRCHOP$H.pred)

metadataMDFCI$GCPH.pred2 <- metadataMDFCI$GCPH.pred /
  sd(metadataMDFCI$GCPH.pred) * sd(metadataMDFCI$H.pred)

mat2[1, 1:3] <- CalcHR(PFS5 ~ I(H.pred/10), data = metadataIDRC)
mat2[1, 4:6] <- CalcHR(PFS5 ~ I(GCPH.pred2/10), data = metadataIDRC)

mat2[2, 1:3] <- CalcHR(PFS5 ~ I(H.pred/10), data = metadataLLMPPRCHOP)
mat2[2, 4:6] <- CalcHR(PFS5 ~ I(GCPH.pred2/10), data = metadataLLMPPRCHOP)

mat2[3, 1:3] <- CalcHR(OS5 ~ I(H.pred/10), data = metadataMDFCI)
mat2[3, 4:6] <- CalcHR(OS5 ~ I(GCPH.pred2/10), data = metadataMDFCI)

mat2

##              N     HR (95\\% CI)      P-value    N     HR (95\\% CI)
## IDRC (PFS)   "470" "1.11 (1.04,1.17)" "0.000572" "470" "1.07 (1.01,1.13)"
## LLMPP (PFS) "220" "1.10 (1.00,1.20)" "0.0438"   "220" "1.09 (0.99,1.19)"
## MDFCI (OS)   "67"  "1.32 (1.14,1.54)" "0.000318" "67"  "0.96 (0.83,1.12)"
##              P-value
## IDRC (PFS)   "0.0131"
## LLMPP (PFS) "0.0794"
## MDFCI (OS)   "0.63"
```

Third, the Cox proportional hazards rates for the REGS classifiers are adjusted for IPI.

```r
mat3 <- matrix(NA, nrow = 3, ncol = 3*2)
row.names(mat3) <- c("IDRC (PFS)", "LLMPP (PFS)", "MDFCI (OS)")

colnames(mat3) <- rep(c("N", "HR (95\\% CI)", "P-value"), 2)
```

```r
mat3[1, 1:3] <- CalcHR2(PFS5 ~ H.class + ipi.hl, data = metadataIDRC)
mat3[1, 4:6] <- CalcHR2(PFS5 ~ GCPH.class + ipi.hl, data = metadataIDRC)

mat3[2, 1:3] <- CalcHR2(PFS5 ~ H.class + ipi.hl2, data = metadataLLMPPRCHOP)
mat3[2, 4:6] <- CalcHR2(PFS5 ~ GCPH.class + ipi.hl2, data = metadataLLMPPRCHOP)

mat3[3, 1:3] <- CalcHR2(OS5 ~ H.class + ipi.hl, data = metadataMDFCI)
mat3[3, 4:6] <- CalcHR2(OS5 ~ GCPH.class + ipi.hl, data = metadataMDFCI)

mat3

##              N     HR (95\\% CI)       P-value    N     HR (95\\% CI)
## IDRC (PFS)   "424" "2.52 (1.64,3.87)"  "2.65e-05" "424" "1.46 (0.99,2.16)"
## LLMPP (PFS) "180" "2.52 (1.13,5.64)"  "0.0237"   "180" "1.37 (0.68,2.74)"
## MDFCI (OS)   "63"  "4.05 (1.13,14.51)" "0.0318"   "63"  "0.84 (0.25,2.76)"
##              P-value
## IDRC (PFS)   "0.0562"
## LLMPP (PFS) "0.375"
## MDFCI (OS)   "0.769"
```

Fourth, the Cox proportional hazards rates for the REGS predictors are adjusted for IPI.

```r
mat4 <- matrix(NA, nrow = 3, ncol = 3*2)
row.names(mat4) <- c("IDRC (PFS)", "LLMPP (PFS)", "MDFCI (OS)")

colnames(mat4) <- rep(c("N", "HR (95\\% CI)", "P-value"), 2)
```

```r
mat4[1, 1:3] <- CalcHR(PFS5 ~ I(H.pred/10) + ipi.hl, data = metadataIDRC)
mat4[1, 4:6] <- CalcHR(PFS5 ~ I(GCPH.pred2/10) + ipi.hl, data = metadataIDRC)

mat4[2, 1:3] <- CalcHR(PFS5 ~ I(H.pred/10) + ipi.hl2, data = metadataLLMPPRCHOP)
mat4[2, 4:6] <- CalcHR(PFS5 ~ I(GCPH.pred2/10) + ipi.hl2, data = metadataLLMPPRCHOP)

mat4[3, 1:3] <- CalcHR(OS5 ~ I(H.pred/10) + ipi.hl, data = metadataMDFCI)
mat4[3, 4:6] <- CalcHR(OS5 ~ I(GCPH.pred2/10) + ipi.hl, data = metadataMDFCI)

mat4

##              N     HR (95\\% CI)      P-value    N     HR (95\\% CI)
## IDRC (PFS)   "424" "1.09 (1.03,1.16)" "0.00595"  "424" "1.05 (1.00,1.11)"
## LLMPP (PFS)  "180" "1.11 (1.00,1.23)" "0.0433"   "180" "1.04 (0.94,1.15)"
## MDFCI (OS)   "63"  "1.34 (1.15,1.57)" "0.000237" "63"  "0.95 (0.83,1.07)"
##              P-value
## IDRC (PFS)   "0.0733"
## LLMPP (PFS)  "0.467"
## MDFCI (OS)   "0.387"
```

The results are combined and saved to a .csv file.

```r
write.csv(rbind(mat1, mat3, mat2, mat4), file = "Output/cox.comparison.csv")
```

13 Differentially Expressed Genes and GO Enrichment

13.1 Differentially Expression in Clinical Data

Function for calculating sample mean with confidence interval for each row of a matrix.

```
ci.matrix <- function(x, alpha = 0.05){
  mean  <- rowMeans(x, na.rm = TRUE)
  n     <- rowSums(!is.na(x)) #ncol(x)
  se    <- rowSds(x, na.rm = TRUE) / sqrt(n)
  error <- qt(1 - alpha / 2, df = n - 1) * se
  ll    <- mean - error
  ul    <- mean + error
  return <- cbind(n, mean, se, ll, ul)
  colnames(return) <- c("Obs", "Mean", "Std. Err.",
                        "Lower limit", "Upper limit")
  return(return)
}
```

Only probesets associated with a gene symbol is used in the analysis.

```
keep <- rownames(HGU133Plus2[!is.na(HGU133Plus2[, "Gene.Symbol" ]),])
```

A dataset is created consisting only of the resistant and sensitive subgroups.

```
what <- "CHO.class"
data <- metadataIDRC[metadataIDRC[, what] %in% c("Resistant", "Sensitive")  ,]

## The intermediate level is dropped
data[, what] <- drop.levels(data[, what])
```

The data are stored in the variables x and y.

```
y <- data[, what]

x <- exprs(GEPIDRC)[keep, data$Array.Data.File]
```

The package limma is used to discover differential expressed genes between CHO sensitive and CHO resistant patients.

```
design <- model.matrix(~ 0 + y)

colnames(design) <- c("Resistant", "Sensitive")

contrast.matrix <- makeContrasts(contrasts = "Resistant - Sensitive",
                                 levels = design)

colnames(design) <- c("Resistant", "Sensitive")

fit <- lmFit(x, design)
fit2 <- contrasts.fit(fit, contrast.matrix)
ebayes <- eBayes(fit2)

tab.limma <- topTable(ebayes, coef = 1, number = nrow(x))
```

Confidence intervals for the mean expression level for each gene in each group are calculated and added to the result.

```r
Resistant.ci <- ci.matrix(x = x[,y == "Resistant"])

## Warning in qt(1 - alpha/2, df = n - 1):  NaNs produced

Sensitive.ci <- ci.matrix(x[,y == "Sensitive"])

## Warning in qt(1 - alpha/2, df = n - 1):  NaNs produced

tab.limma$"Mean, Resistant" <-
  paste(round(Resistant.ci[row.names(tab.limma), "Mean"], 2), " (",
        round(Resistant.ci[row.names(tab.limma), "Lower limit"], 2), "-",
        round(Resistant.ci[row.names(tab.limma), "Upper limit"], 2), ")", sep = "")

tab.limma$"Mean, Sensitive" <-
  paste(round(Sensitive.ci[row.names(tab.limma), "Mean"], 2), " (",
        round(Sensitive.ci[row.names(tab.limma), "Lower limit"], 2), "-",
        round(Sensitive.ci[row.names(tab.limma), "Upper limit"], 2), ")", sep = "")
```

The gene symbol and gene title is added.

```r
result <-
  cbind(HGU133Plus2[row.names(tab.limma), c("Gene.Symbol", "Gene.Title") ],
        tab.limma)
```

An x is added for the genes that are also present in the classifiers for C, H, and O.

```r
result$C <- result[, "Gene.Symbol"] %in%
  HGU133Plus2[names(Cyclophosphamide.reisitance.class.coef)[-1], "Gene.Symbol"]
result$C <- ifelse(result$C, "x", "")

result$H <- result[, "Gene.Symbol"] %in%
  HGU133Plus2[names(Doxorubicin.reisitance.class.coef)[-1], "Gene.Symbol"]
result$H <- ifelse(result$H, "x", "")

result$O <- result[, "Gene.Symbol"] %in%
  HGU133Plus2[names(Vincristine.reisitance.class.coef)[-1], "Gene.Symbol"]
result$O <- ifelse(result$O, "x", "")
```

Only the genes that are differentially expressed at the 0.05 level and assiciated with a log fold change greater than 1 are considered significant.

```r
result <- as.data.frame(result)[, c("Gene.Symbol", "Gene.Title",
                                    "Mean, Resistant", "Mean, Sensitive",
                                    "logFC", "adj.P.Val", "C", "H", "O")]


select <- abs(result$logFC) > 1 & result$adj.P.Val < 0.05
select[is.na(select)] <- FALSE
result <- result[select, ]
```

13.2 GO enrichment

In this section find the GO terms that are overrepresented in the list of differentially expressed genes found in the previous section.

All required information for the test is put into an object.

```
unique.genes <- unique(unlist(mget(rownames(result), envir = hgu133plus2ENTREZID)))
universeGeneIds <- unique(unlist(mget(row.names(tab.limma), envir = hgu133plus2ENTREZID)))

params <- new("GOHyperGParams",
              geneIds         = unique.genes,
              universeGeneIds = universeGeneIds,
              annotation      = "hgu133plus2.db",
              ontology        = "BP",
              pvalueCutoff    = 1,
              conditional     = FALSE,
              testDirection   = "over")
```

We can then apply the overrepresentation analysis.

```
GO.IDRC <- hyperGTest(params)
```

We get the results from the overrepresentation analysis through the summary.

```
GO.IDRC.s <- summary(GO.IDRC)
```

To take multiple testing into account the function p.adjust is used to adjust the p-values using holms method.

```
GO.IDRC.s$Adj.Pvalue <- p.adjust(GO.IDRC.s$Pvalue)

## Only GO terms associated with an adjusted p-value below 0.05 is considered overrepresented.
GO.IDRC.s <- GO.IDRC.s[GO.IDRC.s$Adj.Pvalue < 0.05,
                       c("GOBPID",  "OddsRatio", "ExpCount", "Count", "Size",
                         "Pvalue", "Adj.Pvalue", "Term")]
```

We expand the table of differentially expressed genes generated in the previoues section with columns representing each overrepresented GO term. In each of these columns are added an x if the gene was present in the GO term. This is done to observe which of the differentially expressed genes are represented by each significant GO term.

```
x <- as.list(hgu133plus2GO2ALLPROBES)
GO.result <- result
for(GO.id in GO.IDRC.s[,1])
   GO.result[, GO.id] <-
    ifelse(rownames(GO.result) %in% x[[GO.id]], "x", "")
```

Finally, the results are saved in an Excel document.

```
WriteXLS("GO.result", row.names = FALSE,
           file.path(table.output, "GO.IDRC.DE.CHO.Class.xls"))
```

13.2.1 GO Enrichment, Step by Step

The test conducted for each GO term is illustrated below.

```
## Get the conversion from probeset names to GO id
GO.look <- mget(row.names(tab.limma), hgu133plus2GO)

## Constrict to all probesets associated with GO id
has.go <- sapply(GO.look,
                 function(x) {if (length(x) == 1 && is.na(x)) FALSE else TRUE})

## Constrict to all probesets associated with biological process
has.BP <- sapply(GO.look[has.go], function(x) any(grepl("BP", x)))
```

```r
## find ENTREZ gene ID's for the significant probesets.
geneIds = unlist(mget(rownames(result), envir = hgu133plus2ENTREZID))
## And all the probesets tested.
universeGeneIds = unlist(mget(row.names(tab.limma), envir = hgu133plus2ENTREZID))

## Restict to those associated with biological process
geneIds <- geneIds[names(geneIds) %in% names(has.BP)[has.BP]]
universeGeneIds <- universeGeneIds[names(universeGeneIds) %in% names(has.BP)[has.BP]]

## Restict to non-duplicated genes
geneIds <- geneIds[!duplicated(geneIds)]
universeGeneIds <- universeGeneIds[!duplicated(universeGeneIds)]

## Extract the probesets associated with each GO.id
x <- as.list(hgu133plus2GO2ALLPROBES)


## Initialize a vector for storing the count of genes that are differentially expressed between
## sensitive and resistant as well as associated with each GO term.
count <- vector()

## Initialize a vector for storing the count of genes that are associated with each GO term.
size  <- vector()

## For each significant GO term fill in the counts
sig.probes <- intersect(rownames(result), names(geneIds))
av.probes  <- intersect(row.names(tab.limma), names(universeGeneIds))
for(GO.id in GO.IDRC.s[,1]){

  c <- table(sig.probes %in% x[[GO.id]])
  s <- table(av.probes  %in% x[[GO.id]])

  count <- c(count, c["TRUE"])
  size  <- c(size,  s["TRUE"])
}

## Check the counts matches that calculated by the function hyperGTest
all(GO.IDRC.s$Count == count)

## [1] TRUE

all(GO.IDRC.s$Size  == size)

## [1] TRUE

## In order to perform the hypergeometric test we need to know:
## 1. The number of significant genes:
(lc <- length(geneIds))

## [1] 216

## 2. The number of genes tested:
(ls <- length(universeGeneIds))

## [1] 14675

## Then we may perform the tests
p.vals <- phyper(count-1, size, ls - size, lc, lower.tail=FALSE)

## Check that all P-values matches those calculated by hyperGTest
all(p.vals == GO.IDRC.s$Pvalue)

## [1] TRUE
```
