# Supplementary material for: Predicting response to multidrug regimens in cancer patients using cell line experiments and regularised regression models
Source: BMC Cancer. 2015 Apr 8;15:235. doi: 10.1186/s12885-015-1237-6 (PMC4396063; doi:10.1186/s12885-015-1237-6)
Supplement: Additional file 2: — Contains supplementary figures and tables. [file 12885_2015_1237_MOESM2_ESM.docx]

**Additional file 2 for**

# Predicting response to multidrug regimens in cancer patients using cell line experiments and regularised regression models

Steffen Falgreen^1^, Karen Dybkær^1,4,5^, Ken H. Young^2^, Zijun Y. Xu-Monette^2^, Tarec C. El-Galaly^1,4^, Maria Bach Laursen^1^, Julie S. Bødker^1^, Malene K. Kjeldsen^1^, Alexander Schmitz^1^, Mette Nyegaard^1,3^, Hans Erik Johnsen^1,4,5^, Martin Bøgsted^1,4,5^

**^1^**Department of Haematology, Aalborg University Hospital, Aalborg, Denmark

**^2^**Department of Hematopathology, The University of Texas MD Anderson Cancer Center, Houston, TX, United States

**^3^**Department of Biomedicine, Aarhus University, Aarhus, Denmark

**^4^**Department of Clinical Medicine, Aalborg University, Aalborg, Denmark

**^5^**Clinical Cancer Research Center, Aalborg University Hospital, Aalborg, Denmark

# Contents

|  | **Supplementary Figures** |  |
| --- | --- | --- |
| Figure S1 | Leave-one-out cross validation for REGS classifiers | **2** |
| Figure S2 | Regularisation curves for the gene expression classifiers | **2** |
| Figure S3 | Leave-one-out cross validation for REGS predictors | **3** |
| Figure S4 | Regularisation curves for the gene expression predictors | **3** |
| Figure S5 | Performance of REGS tested on MDFCI**.** | **4** |
| Figure S6 | Performance of REGS tested on UAMS (Negative Control)**.** | **5** |
|  | **Supplementary Tables** |  |
| Table S1 | Information on the cell lines | **6** |
| Table S2 | The gene list for the REGS classifiers for each of the three drugs | **10** |
| Table S3 | The gene list for the REGS predictors for each of the three drugs | **17** |
| Table S4 | Cox proportional hazards analyses of performance of REGS classifiers | **20** |
| Table S5 | Cox proportional hazards analyses of performance of REGS predictors | **21** |

**­­­**

Figure S1. Leave-one-out cross validation **for the parameters α and λ in the elastic net classification.** In panels A, B, and C the classification error for varying values of α are shown for Cyclophosphamide, Doxorubicin, and Vincristine, respectively. In panels D, E, and F the classification error is plotted against λ for the α values resulting in the minimum classification error for each of the three drugs. The grey area represents a one-standard deviation band.

Figure S2. Regularisation curves for the gene expression classifiers **for the three drugs.** In panels A, B, and C the coefficients’ paths are shown. The red line indicates the model chosen through leave-one-out cross validation. The genes for the 20 probe-sets associated with the largest coefficients in the chosen gene expression classifers are displayed. Positive coefficients indicate that high expression values for the associated gene are related to resistance toward the drug and vice versa for negative coefficients.

Figure S3. Leave-one-out cross validation **for the parameters α and λ in the elastic net regression.** In panels A, B, and C the mean squared prediction error (MSPE) for varying values of α are shown for Cyclophosphamide, Doxorubicin, and Vincristine, respectively. In panels D, E, and F the MSPE is plotted against λ for the α values resulting in the minimum MSPE for each of the three drugs. The grey area represents a one-standard deviation band.

Figure S4. Regularisation curves for the gene expression predictors **for the three drugs.** In panels A, B, and C the coefficients’ paths are shown. The red line indicates the model chosen through leave-one-out cross validation. The genes for the 20 probe-sets associated with the largest coefficients in the chosen gene expression predictors are displayed. Positive coefficients indicate that high expression values for the associated gene are related to resistance toward the drug and vice versa for negative coefficients.

Figure S5. **The association between OS and the predicted level of sensitivity for the combined REGS for CHO in the MDFCI dataset.** In panel A the probability of being sensitive (one minus the probability of being resistant) according to the REGS classifier is plotted for each patient. Based on the probabilities the patients are categorised into tertiles with those deemed sensitive, intermediate, and resistant indicated by green, blue, and red. Kaplan-Meier curves for PFS are shown in panel D. Panel B shows estimated log HR versus predicted resistance index modelled by a RCS-model with four knots for the REGS predictor for CHO adjusted for IPI. Panel E shows the corresponding survival curves generated by the fitted Cox proportional hazards regression. The survival curves are generated for the values marked by arrows in Panel B. Panels C and F illustrate an analysis of ROC curves for prediction of the combination therapy CHO where all curves are shown with 95% CI. Panel C Shows AUC under the ROC curves plotted against time for the CHO REGS classifier (green) and predictor (blue). Panel F shows the difference in AUC plotted against time.

Figure S6. Negative control**.** Analysis of the association between OS and the predicted level of sensitivity for the combined REGS for CHO in the UAMS dataset. In panel A the probability of being sensitive according to the REGS classifier is plotted for each patient. Based on the probabilities the patients are categorised into tertiles with those deemed sensitive, intermediate, and resistant indicated by green, blue, and red. Kaplan-Meier curves for PFS are shown in panel D. Panel B shows estimated log HR versus predicted resistance index modelled by a RCS-model with four knots for the REGS predictor for CHO adjusted for IPI. Panel E shows the corresponding survival curves generated by the fitted Cox proportional hazards regression. The survival curves are generated for the values marked by arrows in Panel B. Panels C and F illustrate an analysis of ROC curves for prediction of the combination therapy CHO where all curves are shown with 95% CI. Panel C Shows AUC under the ROC curves plotted against time for the CHO REGS classifier (green) and predictor (blue). Panel F shows the difference in AUC plotted against time.

Table S1. **Characteristics** **on the cell lines.** From left to right 1) cell line, 2) supplier information, 3) culture conditions, 4-12) passage nr., resistance level, and resistance class for C, H, and O, and 13) Established from. Regarding supplier information: 1, Kind gift from Jose A. Martinez, MD, PhD, Spain; 2, Kind gift from Hans Messner, Canada; 3 Kind gift from Dr. Steven T. Rosen; JCRB, Japanese Collection of Research Bioresources; DSMZ, German Collection of Microorganisms and Cell Cultures. The cell lines were cultured under standard conditions, at 37°C in a humidified atmosphere of 95% air and 5% CO_2,_ with the appropriate culture medium, e.g., RPMI1640 or IMDM, fetal calf serum (FCS) or human serum (HS), supplemented with 1% penicillin and streptomycin, *with 55 μM 2-mercaptoethanol and **with 2 mM L-glutamine. Regarding passage nr. "," designate new stock thawed and "/" designate same stock.

| Cell line | Supplier | Maintenance Culture medium | Cyclophosphamide | | | Doxorubicin | | | Vincristine | | | Established from |
| --- | --- | --- | --- | --- | --- | --- | --- | --- | --- | --- | --- | --- |
|  |  |  | Passage nr. | AUC | Class | Passage nr. | AUC | Class | Passage nr. | AUC | Class |  |
| DB | DSMZ Acc. 539 | RPMI+20% FCS | P6, P5/P6 | 346 (329, 351) | R | P8/P10, P7 | 276 (261, 286) | R | P8, P5, P6 | 131 (119, 139) | R | Established from ascites of a 45-year-old Caucasian man with diffuse large cell lymphoma |
| FARAGE | 1 | RPMI+10% FCS | P16/P17/P19 | 311 (296, 323) | R | P8/P12/P14 | 180 (164, 198) | S | P13/P14, P5/P6 | 56 (47, 60) | S | The Farage cell line was adapted to culture in 1990 from a lymph node biopsy of a patient with diffuse large cell non-Hodgkin's lymphoma (DLCL). Derived from metastatic site: lymph node, non-Hodgkin's B cell lymphoma, female |
| HBL-1 | 1 | RPMI+10% FCS | P9/P11/P14 | 226 (202, 241) | I | P8/P9, P4 | 272 (264, 277) | I | P16, P5/P6 | 85 (71, 91) | I | Established from the pleural effusion of a patient with malignant lymphoma, diffuse, large cell. |
| MC-116 | DSMZ Acc. 82 | RPMI+15% FCS | - | - | - | P10/P11/P15 | 272 (235, 301) | I | P10/P12, P12 | 62 (33, 118) | S | Ascites male, B-cell undifferentiated lymphoma, EBNA negative, negative for Fc receptors and negative for complement receptors |
| NU-DHL-1 | DSMZ Acc. 583 | RPMI+10% FCS | P9/P12/P16 | 172 (142, 191) | S | P5,P20,P19 | 201 (184, 212) | S | - | - | - | Established from the left inguinal lymph node of a 73-year-old Caucasian man with B-cell Non-Hodgkin lymphoma (B-NHL, diffuse large cell lymphoma, non-cleaved cell type) in 1982 |
| NU-DUL-1 | DSMZ Acc. 579 | RPMI+15% FCS | P10/P13, P6/P8 | 214 (181, 227) | S | P15/P17/P19, P4 | 224 (192, 229) | I | P9,P10,P18 | 90 (79, 95) | I | Peritonial effusion; derived from metastatic site: malignant cerebrospinal fluid, undifferentiated lymphoma, non-Burkitt's type |
| OCI-Ly19 | 1 | RPMI+10% FCS | P13/P17/P18 | 202 (161, 214) | S | P11, P7/P9 | 167 (157, 179) | S | P8/P11/P16 | 54 (40, 68) | S | Established from the bone marrow of a 27-year-old woman with B-cell Non-Hodgkin lymphoma (B-NHL) (diffuse large cell lymphoma, DLCL, stage 4B, at relapse) in 1987 |
| OCI-Ly3 | 1 | IMDM+20% HS* | P17, P7/P8 | 262 (247, 285) | I | P17, P10, P9 | 253 (229, 258) | I | P17, P5,P10 | 75 (65, 81) | I | Established from a bone marrow aspirate and peripheral blood from a NHL patient during relapse (diffuse large cell) in 1987. |
| OCI-Ly7 | 1 | RPMI+10% FCS | P22, P16/P22 | 258 (191, 282) | I | P11/P12/P15, P11 | 325 (312, 334) | S | P6/P8, P8 | 114 (103, 119) | R | Established from a bone marrow aspirate and peripheral blood from a NHL patient during relapse (diffuse large cell) in 1987. |
| RIVA | 1 | RPMI+10% FCS | P6/P8/P17 | 296 (275, 318) | R | P17, P9/P11 | 327 (316, 337) | R | P10/P19,P21,P5 | 109 (91, 117) | R | Established from the peripheral blood of a 57-year-old woman with B-cell non-Hodgkin lymphoma (B-NHL, lymphocytic, small cell type progressing to large non-convoluted cell type) in the refractory terminal stage in 1977; assigned to ABC-like lymphoma subtype (activated B-cell); cell line also known as Riva |
| SU-DHL-4 | DSMZ Acc. 495 | RPMI+10% FCS | - | - | - | P16, P14 | 289 (278, 293) | R | - | - | - | Established from the peritoneal effusion of a 38-year-old man with B-NHL (diffuse large cell, cleaved cell type; originally described as "diffuse histiocytic lymphoma") in 1975 |
| SU-DHL-5 | DSMZ Acc. 571 | RPMI+20% FCS | P13/P14/P17 | 165 (148, 174) | S | P9/P11, P13 | 202 (188, 210) | S | P10/P21, P6 | 58 (46, 71) | S | Established from a lymph node of a 17-year-old woman with B-cell non-Hodgkin lymphoma (B-NHL), described at the time as diffuse large cell, noncleaved cell type |
| SU-DHL-8 | 1 | RPMI+10% FCS** | P15, P3/P19 | 271 (242, 283) | I | P6/P9/P11 | 222 (208, 230) | S | P11/P13/P19 | 126 (73, 127) | R | Established from the pleural effusion of a 59-year-old man with B-cell non-Hodgkin lymphoma (B-NHL), described at the time as diffuse large cell lymphoma, noncleaved cell type and later as diffuse histiocytic lymphoma |
| U2932 | 1 | RPMI+10% FCS | P14/P16, P19 | 330 (321, 333) | R | P9/P11/P14 | 295 (286, 299) | R | P10,P11,P13 | 85 (77, 91) | I | Established in 1996 from the ascites of a 29-year-old woman with diffuse large B cell lymphoma, who 16 years earlier was diagnosed with advanced stage Hodgkin lymphoma, and relapsed several times after multiple chemo- and radiotharapy regimens to complete remissions; cells were described to overexpress BCL-2, BCL-6 and p53 |
| AMO-1 | DSMZ Acc. 538 | RPMI+20% FCS | P12/P15/P17 | 395 (391, 395) | R | P9, P7/p14 | 269 (262, 282) | S | P7, P5/P7 | 116 (105, 125) | I | Established from the ascitic fluid of a 64-year-old woman with plasmacytoma (IgAkappa) of the duodenum two months following resection of the tumor mass (additional therapy not specified) in 1984; plasmacytoma is related to multiple myeloma |
| KMM-1 | JCRB | RPMI+10% FCS | P19/P20/P22 | 295 (280, 305) | S | P11, P4/P7 | 346 (323, 352) | R | P7/P9/P11 | 133 (125, 135) | R | Japanese male, 62 years old with multiple myeloma. From a subcontaneus plasmacytoma. The patient responded poorly to treatment and died 6 months after diagnosis. Melphalan, vincristine, procarbarzine and prednisolone was the initial treatment. |
| KMS-11 | JCRB | RPMI+10% FCS | P13/P14/P16 | 318 (300, 323) | I | P9, P4/P7 | 356 (343, 361) | R | P11, P7/P9 | 123 (107, 127) | I | Japanese female, 67 years old. Myeloma pleural effusion infiltration (IgGk) |
| KMS-12-BM | DSMZ Acc. 551 | RPMI+20% FCS | P5/P8/P10 | 328 (316, 332) | R | P7/P14, P7 | 331 (308, 338) | R | P4/P6, P7 | 112 (79, 118) | I | Established from the bone marrow of a 64-year-old woman with multiple myeloma in 1988; sister cell line of KMS-12-PE |
| KMS-12-PE | DSMZ Acc. 606 | RPMI+20% FCS | P14/P15, P7 | 309 (306, 310) | I | P12, P6/P8 | 340 (315, 352) | R | P11, P4/P7 | 121 (72, 126) | I | Established in 1987 from the pleural effusion of a 64-year-old woman with refractory, terminal multiple myeloma (Ig-non-producing) after combination chemotherapy; described to carry t(11:14)(q13:q32) rearrangement; sister cell line of KMS-12-BM |
| LP-1 | DSMZ Acc. 41 | IMDM+10% FCS | P14/P15, P7 | 308 (304, 311) | I | P9, P9, P6 | 316 (292, 335) | I | P4/P7, P6 | 187 (151, 219) | R | Established from the peripheral blood of a 56-year-old woman with multiple myeloma (IgG, EBV-negative) in leukemic transformation (refractory, terminal) in 1986 |
| MM1S | 3 | RPMI+10% FCS | P21, P17/P19 | 242 (228, 245) | S | P14, P4/P6 | 228 (198, 238) | S | P6/P8/P10 | 90 (79, 99) | S | The MM.1S cell line was subcloned from the MM.1 cell line by limiting dilution to isolate a subline that is sensitive to the killing effects of glucocorticoids. The MM.1 cell line was derived from peripheral blood cells of a patient with immunoglobulin A myeloma. The patient whose treatment regimen included glucocorticoids was in the leukemic phase of MM |
| MOLP-8 | DSMZ Acc. 569 | RPMI+20% FCS | P16, P9/P14 | 249 (225, 256) | S | P7, P7, P6 | 253 (226, 289) | S | P4, P5/P13 | 149 (132, 158) | R | Established from the peripheral blood of a 52-year-old Japanese man with multiple myeloma (stage IIIA, type IgD lambda) in 2002 |
| NCI-H929 | DSMZ Acc. 163 | RPMI+20% FCS | P19/P21/P17 | 322 (308, 327) | I | P9, P15, P7 | 291 (264, 300) | S | P3/ P7,P13 | 149 (137, 168) | R | Established from the pleural effusion of a 62-year-old white woman with myeloma (IgAkappa) at relapse; cells were described as follows: cells synthesize high amounts of immunoglobulin, MYC proto-oncogene is rearranged and MYC RNA is expressed, cells are EBV-negative; patented cell line |
| OPM-2 | DSMZ Acc. 50 | RPMI+10% FCS | P8, P18/P20 | 303 (289, 306) | S | P4, P19, P7 | 329 (314, 337) | I | P7/P9/P15 | 95 (80, 110) | S | Established from the peripheral blood of a 56-year-old woman with multiple myeloma (IgG lambda) in leukemic phase (relapse, terminal) in 1982 |
| RPMI-8226 | DSMZ Acc. 402 | RPMI+10% FCS | P6/P9/P13 | 350 (345, 353) | R | P11, P11, P8 | 297 (291, 306) | I | P7/P10,P7 | 111 (98, 120) | S | Established from the peripheral blood of a 61-year-old man with multiple myeloma (IgG lambda-type) at diagnosis in 1966; described to produce and secrete only lambda light chains (but not heavy chains) |
| U-266 | DSMZ Acc. 9 | RPMI+10% FCS | P12/P14/P16 | 376 (372, 378) | R | P19, P5/P6 | 325 (299, 341) | I | P18,P4,P4 | 90 (73, 101) | S | Established from the peripheral blood of a 53-year-old man with IgE-secreting myeloma (refractory, terminal) in 1968; cells were described to produce IgE lambda; possible fusion partner for hybridoma production; cells express mRNA for BCL2 gene |

Table S2. The gene list for the REGS classifiers for each of the three drugs. The columns from left to right: 1) the Affymetrix GeneChip HG-U133 Plus 2.0 array probe-set id, 2) the gene symbol, 3) standard deviation for the 14 DLBCL cell lines, 4-6) weight in logistic classifier for C, H, and O, 7-12) mean expression level for resistant and sensitive cell lines according to C, H, and O, and 13) Gene title.

| Probeset | Gene Symbol | sd | C | H | O | Cyclophosphamide Mean | | Doxorubicin Mean | | Vincristine Mean | | Gene Title |
| --- | --- | --- | --- | --- | --- | --- | --- | --- | --- | --- | --- | --- |
|  |  |  |  |  |  | Resistant | Sensitive | Resistant | Sensitive | Resistant | Sensitive |  |
| (Intercept) |  |  | -0.075 | 0.167 | 0.270 |  |  |  |  |  |  |  |
| 202157_s_at | CELF2 | 2.130 | -0.065 | -0.004 |  | 8.9 (6.7-11.1) | 11.4 (10.8-12) | 9.3 (7.5-11.2) | 10.8 (9.5-12.2) | 10.2 (8.3-12) | 10.2 (8.6-11.8) | CUGBP, Elav-like family member 2 |
| 203695_s_at | DFNA5 | 2.030 | -0.063 |  |  | 5.1 (4.7-5.5) | 6.8 (5-8.5) | 6 (4.8-7.2) | 6.7 (4.9-8.6) | 6.5 (4.4-8.6) | 6 (4.7-7.4) | deafness, autosomal dominant 5 |
| 221933_at | NLGN4X | 2.309 | -0.060 | -0.005 |  | 3.4 (3.2-3.5) | 5.8 (3.5-8) | 3.9 (3.1-4.6) | 5.4 (3.4-7.5) | 3.6 (3.4-3.9) | 5.2 (3.1-7.3) | neuroligin 4, X-linked |
| 210517_s_at | AKAP12 | 3.238 | -0.051 |  |  | 3.5 (3.4-3.7) | 6.1 (2.9-9.3) | 3.7 (3.5-4) | 5.3 (2.8-7.9) | 4.5 (3-5.9) | 5.5 (2.3-8.6) | A kinase (PRKA) anchor protein 12 |
| 242794_at | MAML3 | 2.134 | -0.046 | 0.000 |  | 4 (2.6-5.3) | 6.5 (4.8-8.3) | 4.3 (3.3-5.2) | 6 (4-7.9) | 4.5 (2.7-6.2) | 5.2 (3.5-7) | mastermind-like 3 (Drosophila) |
| 202600_s_at | NRIP1 | 2.744 | -0.045 |  |  | 4.6 (3.2-6.1) | 7.7 (5.8-9.5) | 4.7 (3.2-6.2) | 7 (5.1-8.9) | 6.5 (4.2-8.7) | 6 (3.9-8.1) | nuclear receptor interacting protein 1 |
| 224499_s_at | AICDA | 3.236 | -0.045 |  |  | 4.4 (3.6-5.2) | 6.4 (3.5-9.3) | 5.7 (3.7-7.7) | 5.4 (3.2-7.6) | 4.7 (3-6.4) | 5 (2.8-7.3) | activation-induced cytidine deaminase |
| 226743_at | SLFN11 | 2.007 | -0.037 | -0.060 |  | 5.5 (4.3-6.7) | 7.5 (5.9-9.1) | 5.4 (4.4-6.5) | 7.6 (6-9.1) | 6.2 (4.6-7.7) | 6.6 (4.7-8.6) | schlafen family member 11 |
| 203066_at | CHST15 | 2.634 | -0.031 |  |  | 8.3 (6.2-10.3) | 9.9 (8.5-11.3) | 9.1 (7.5-10.6) | 8.8 (6.7-11) | 8.8 (6.5-11.1) | 8.7 (6.7-10.8) | carbohydrate (N-acetylgalactosamine 4-sulfate 6-O) sulfotransferase 15 |
| 221675_s_at | CHPT1 | 2.378 | -0.029 |  |  | 7.8 (6.2-9.4) | 9.4 (8.5-10.2) | 8.5 (7.1-10) | 8.3 (6.9-9.8) | 7.6 (5.6-9.6) | 7.9 (6.2-9.5) | choline phosphotransferase 1 |
| 1564310_a_at | PARP15 | 1.802 | -0.027 |  |  | 4.7 (3.2-6.1) | 5.1 (4-6.2) | 4.1 (3.4-4.9) | 5.1 (3.8-6.4) | 4.1 (3.2-5) | 5.2 (3.8-6.5) | poly (ADP-ribose) polymerase family, member 15 |
| 218251_at | MID1IP1 | 2.107 | -0.025 |  |  | 5.7 (4-7.5) | 8 (6.4-9.5) | 5.7 (4.2-7.2) | 7 (5.4-8.6) | 6.6 (4.8-8.4) | 8 (6.6-9.4) | MID1 interacting protein 1 |
| 200897_s_at | PALLD | 2.149 | -0.023 |  |  | 5.8 (4.4-7.1) | 7.9 (6-9.9) | 6.6 (4.7-8.4) | 6.9 (4.9-8.8) | 6.6 (4.9-8.2) | 7.1 (5-9.2) | palladin, cytoskeletal associated protein |
| 213793_s_at | HOMER1 | 2.342 | -0.021 |  |  | 7.2 (5.3-9.1) | 9.3 (8.3-10.2) | 7 (5.1-9) | 8.7 (8.4-9.1) | 8 (6.5-9.5) | 8.1 (6.8-9.5) | homer homolog 1 (Drosophila) |
| 201924_at | AFF1 | 2.117 | -0.019 |  |  | 8.3 (6.8-9.9) | 9.8 (7.8-11.8) | 8.9 (7.7-10.2) | 8.8 (6.8-10.8) | 9.5 (8.3-10.8) | 8.5 (6.3-10.8) | AF4/FMR2 family, member 1 |
| 217894_at | KCTD3 | 1.920 | -0.019 |  |  | 6.2 (4.3-8.1) | 8.2 (6.5-9.9) | 6.8 (5.4-8.2) | 7 (4.8-9.2) | 7.1 (5-9.2) | 7.5 (5.8-9.1) | potassium channel tetramerisation domain containing 3 |
| 227396_at | LOC100287223 | 2.028 | -0.018 |  |  | 5.6 (3.9-7.3) | 7.3 (5.7-8.9) | 6.6 (4.9-8.2) | 6.5 (4.8-8.2) | 6.5 (4.9-8.2) | 6 (4.4-7.6) | uncharacterized LOC100287223 /// protein tyrosine phosphatase, receptor type, J |
| 210690_at | KLRC4 | 2.328 | -0.018 |  |  | 3 (2.8-3.2) | 4.6 (2.2-7) | 3.3 (2.9-3.8) | 3.8 (2.1-5.5) | 3.2 (2.8-3.6) | 3.6 (2.3-4.9) | killer cell lectin-like receptor subfamily C, member 4 |
| 206638_at | HTR2B | 2.252 | -0.018 |  |  | 3.2 (3.1-3.4) | 4.7 (2.1-7.3) | 3.7 (2.7-4.7) | 4.1 (2-6.3) | 3.8 (2.6-5) | 3.1 (3-3.3) | 5-hydroxytryptamine (serotonin) receptor 2B, G protein-coupled |
| 224983_at | SCARB2 | 1.950 | -0.015 |  |  | 8.2 (6.5-9.9) | 10.1 (8.3-11.9) | 7.6 (5.5-9.7) | 9.2 (7.6-10.8) | 8.9 (6.4-11.4) | 8.9 (6.7-11.2) | scavenger receptor class B, member 2 |
| 231577_s_at | GBP1 | 2.053 | -0.013 | -0.032 |  | 4.9 (3.9-5.8) | 6.5 (4.8-8.2) | 4.5 (4.3-4.8) | 6.8 (5.2-8.3) | 5.1 (3.9-6.3) | 5.9 (4.3-7.5) | guanylate binding protein 1, interferon-inducible |
| 209732_at | CLEC2B | 2.770 | -0.011 |  |  | 7.1 (5.4-8.9) | 10 (8.2-11.8) | 6.6 (5.3-7.8) | 9 (7-10.9) | 8.1 (6-10.3) | 8.4 (6.7-10) | C-type lectin domain family 2, member B |
| 208892_s_at | DUSP6 | 2.608 | -0.009 | -0.080 |  | 5.2 (3.3-7.2) | 8.2 (6.9-9.5) | 4.5 (3-6) | 8.5 (6.8-10.2) | 6.6 (3.9-9.4) | 6.9 (4.9-9) | dual specificity phosphatase 6 |
| 209138_x_at | IGLC1 | 1.962 | -0.006 |  |  | 11.6 (9.1-14.1) | 14.2 (13.8-14.6) | 11.4 (9.7-13.1) | 12.9 (11.1-14.7) | 13.5 (12.5-14.5) | 13.6 (11.5-15.7) | Immunoglobulin lambda constant 1 (Mcg marker) |
| 224520_s_at | BEST3 | 2.492 | -0.006 |  |  | 3.5 (2.9-4.2) | 5.2 (3.2-7.1) | 3.8 (2.8-4.8) | 4.8 (2.9-6.7) | 4.7 (2.6-6.9) | 4.9 (3.7-6.1) | bestrophin 3 |
| 213241_at | PLXNC1 | 2.364 | -0.005 | -0.008 |  | 6.9 (5.1-8.8) | 8.8 (7-10.5) | 6.7 (5-8.4) | 8.6 (7-10.2) | 7.1 (5.3-8.9) | 8.5 (6.5-10.4) | plexin C1 |
| 212592_at | IGJ | 3.324 | -0.004 |  |  | 9.9 (6.5-13.3) | 12.2 (9.7-14.7) | 10.9 (8-13.8) | 10.9 (8.1-13.6) | 11.3 (8.3-14.3) | 11.7 (8.9-14.4) | immunoglobulin J polypeptide, linker protein for immunoglobulin alpha and mu polypeptides |
| 200670_at | XBP1 | 1.733 | -0.004 |  |  | 9.7 (8.1-11.3) | 11.1 (9.6-12.7) | 10 (8.4-11.5) | 9.9 (8-11.8) | 10.8 (9.1-12.6) | 10.3 (8.3-12.2) | X-box binding protein 1 |
| 201700_at | CCND3 | 1.443 | -0.003 |  |  | 9.1 (8.2-10.1) | 10.5 (9.1-11.9) | 9.7 (8.5-10.8) | 10.5 (9.2-11.7) | 10.1 (8.3-11.8) | 10 (8.7-11.4) | cyclin D3 |
| 215117_at | RAG2 | 2.482 | -0.003 |  |  | 3 (2.9-3.2) | 4.4 (2.2-6.5) | 3 (3-3.1) | 4.4 (2.3-6.4) | 3.8 (1.9-5.7) | 3.7 (2.1-5.4) | recombination activating gene 2 |
| 205632_s_at | PIP5K1B | 1.751 | -0.002 |  |  | 6.4 (4.8-8.1) | 8.5 (7.3-9.7) | 6.8 (5.7-7.9) | 8 (6.6-9.4) | 7.8 (6.3-9.3) | 7.5 (5.7-9.3) | phosphatidylinositol-4-phosphate 5-kinase, type I, beta |
| 221558_s_at | LEF1 | 2.422 | -0.001 |  |  | 7.6 (5.2-10) | 9.6 (8.6-10.5) | 9.3 (7.1-11.5) | 8.7 (7.1-10.4) | 8.6 (6.3-10.8) | 9.2 (7.2-11.2) | lymphoid enhancer-binding factor 1 |
| 201160_s_at | CSDA | 3.852 | -0.001 |  | -0.036 | 8.1 (4.9-11.4) | 9.4 (6.4-12.4) | 8.6 (5.6-11.6) | 8.6 (5.9-11.4) | 7.4 (4.1-10.8) | 10 (7.8-12.3) | cold shock domain protein A |
| 205173_x_at | CD58 | 3.180 |  | -0.143 |  | 9.3 (7.7-10.9) | 9.9 (8.8-11) | 7.5 (5.5-9.5) | 10.3 (9.2-11.4) | 8.7 (6.4-11) | 10.3 (9.1-11.5) | CD58 molecule |
| 210732_s_at | LGALS8 | 1.830 |  | -0.090 |  | 5.8 (4-7.7) | 6.3 (4.6-8.1) | 4.2 (3.2-5.2) | 7.5 (6.4-8.6) | 5 (3.3-6.7) | 6.7 (5-8.4) | lectin, galactoside-binding, soluble, 8 |
| 215001_s_at | GLUL | 3.363 |  | -0.085 |  | 7.7 (5.6-9.8) | 8.9 (6.2-11.5) | 6.3 (3.9-8.7) | 9.1 (7.3-11) | 7.3 (4.6-10) | 7.5 (4.7-10.3) | glutamate-ammonia ligase |
| 206632_s_at | APOBEC3B | 2.246 |  | -0.072 |  | 7.5 (4.7-10.4) | 8.5 (7.2-9.9) | 6.6 (4.3-8.8) | 8.9 (7.3-10.5) | 7.9 (5.6-10.2) | 9 (7.4-10.6) | apolipoprotein B mRNA editing enzyme, catalytic polypeptide-like 3B |
| 206310_at | SPINK2 | 2.861 |  | -0.071 | -0.059 | 6.8 (4.8-8.8) | 6.9 (5-8.8) | 5.7 (4.6-6.7) | 7.5 (5.4-9.6) | 5.6 (4.7-6.4) | 8.7 (6.4-11.1) | serine peptidase inhibitor, Kazal type 2 (acrosin-trypsin inhibitor) |
| 211597_s_at | HOPX | 3.379 |  | -0.068 | -0.001 | 5.1 (2.7-7.4) | 5.2 (2.8-7.5) | 4 (3.6-4.4) | 5.9 (3.2-8.6) | 4.1 (3.6-4.5) | 7.1 (3.9-10.3) | HOP homeobox |
| 213069_at | HEG1 | 1.713 |  | -0.065 |  | 6.6 (4.8-8.4) | 7.7 (6.2-9.1) | 5.4 (4.9-5.9) | 8.4 (7.2-9.5) | 7 (5.4-8.6) | 7.7 (6.1-9.3) | HEG homolog 1 (zebrafish) |
| 229390_at | FAM26F | 2.499 |  | -0.062 |  | 5.3 (3-7.6) | 7 (5.8-8.3) | 4.4 (3.5-5.4) | 7.7 (6.1-9.3) | 6.2 (3.7-8.6) | 6.4 (3.9-8.9) | family with sequence similarity 26, member F |
| 219049_at | CSGALNACT1 | 1.868 |  | -0.061 |  | 4.4 (3.3-5.4) | 6.3 (4.3-8.3) | 3.7 (3.5-3.8) | 6.5 (4.8-8.2) | 4.6 (3.4-5.9) | 5 (3.4-6.5) | chondroitin sulfate N-acetylgalactosaminyltransferase 1 |
| 221690_s_at | NLRP2 | 2.575 |  | -0.061 |  | 6.5 (4.5-8.4) | 6.1 (4.2-8) | 4.6 (3.9-5.3) | 6.5 (4.7-8.4) | 5.2 (4-6.4) | 6.9 (4.9-9) | NLR family, pyrin domain containing 2 |
| 203349_s_at | ETV5 | 1.781 |  | -0.055 |  | 7.2 (5.6-8.7) | 8.1 (6.9-9.4) | 5.9 (5-6.7) | 8.7 (8-9.5) | 7.1 (5.4-8.8) | 8.3 (7.4-9.1) | ets variant 5 |
| 203913_s_at | HPGD | 1.842 |  | -0.055 | -0.017 | 4.6 (2.8-6.5) | 5.3 (3.3-7.2) | 3.4 (3.2-3.6) | 5.7 (3.9-7.5) | 3.7 (3-4.4) | 5.6 (3.5-7.6) | hydroxyprostaglandin dehydrogenase 15-(NAD) |
| 227792_at | ITPRIPL2 | 1.883 |  | -0.055 |  | 4.6 (2.9-6.3) | 5.5 (3.1-7.9) | 3.6 (3.5-3.7) | 7.2 (5.2-9.2) | 5.6 (3-8.2) | 4.6 (2.9-6.2) | inositol 1,4,5-trisphosphate receptor interacting protein-like 2 |
| 201426_s_at | VIM | 3.025 |  | -0.050 |  | 10 (7.2-12.8) | 12.4 (10.3-14.5) | 8.4 (6-10.8) | 11.8 (9.7-13.9) | 10.7 (7.9-13.6) | 12.2 (10.1-14.2) | vimentin |
| 207777_s_at | SP140 | 1.951 |  | -0.048 |  | 6.5 (5.3-7.7) | 6.6 (5.1-8.2) | 5.4 (4.3-6.5) | 7.3 (6.4-8.2) | 5.6 (4.2-7.1) | 6.7 (5.7-7.7) | SP140 nuclear body protein |
| 201599_at | OAT | 1.981 |  | -0.047 |  | 9.9 (7.6-12.1) | 10.8 (10.3-11.3) | 9.9 (8-11.8) | 11.1 (10.5-11.8) | 10.2 (7.9-12.4) | 10.6 (9.8-11.3) | ornithine aminotransferase |
| 212192_at | KCTD12 | 2.416 |  | -0.046 |  | 5.6 (3.5-7.7) | 6.5 (4.1-8.9) | 4.2 (3-5.4) | 7.8 (6.4-9.2) | 6.3 (4.2-8.5) | 6.5 (4.2-8.7) | potassium channel tetramerisation domain containing 12 |
| 204836_at | GLDC | 2.207 |  | -0.043 |  | 7.7 (5.5-9.9) | 9.5 (7.5-11.5) | 6.9 (5.3-8.6) | 9.7 (7.8-11.6) | 8 (5.4-10.6) | 8.6 (6.3-11) | glycine dehydrogenase (decarboxylating) |
| 225673_at | MYADM | 2.228 |  | -0.039 |  | 6.9 (4.8-9) | 6.8 (4.8-8.9) | 4.8 (4.2-5.3) | 7.8 (5.8-9.7) | 7.3 (5.1-9.4) | 7.8 (6.1-9.4) | myeloid-associated differentiation marker |
| 201300_s_at | PRNP | 1.953 |  | -0.037 |  | 9.7 (8.9-10.6) | 9.8 (9.2-10.5) | 8.7 (7.5-9.8) | 10.1 (9.7-10.4) | 8.9 (7.5-10.3) | 9.3 (7.3-11.2) | prion protein |
| 201859_at | SRGN | 2.366 |  | -0.037 |  | 11.2 (8.9-13.5) | 10.8 (9.6-12) | 9.5 (7.2-11.8) | 11.4 (10.2-12.6) | 9.9 (7.6-12.3) | 11.5 (10.2-12.7) | serglycin |
| 202336_s_at | PAM | 2.332 |  | -0.036 |  | 7.1 (5-9.2) | 7.7 (6.2-9.3) | 6.1 (4.2-8) | 8 (6.5-9.5) | 6.5 (3.9-9.1) | 8.5 (7.7-9.2) | peptidylglycine alpha-amidating monooxygenase |
| 225626_at | PAG1 | 2.420 |  | -0.034 |  | 7.4 (4.4-10.3) | 8.1 (5.5-10.6) | 7.5 (4.6-10.3) | 8.6 (6.5-10.7) | 7.4 (4.7-10.2) | 8.6 (5.9-11.2) | phosphoprotein associated with glycosphingolipid microdomains 1 |
| 227404_s_at | LOC100653132 | 2.085 |  | -0.032 |  | 8.6 (7-10.3) | 7.5 (5.4-9.5) | 7.2 (5.3-9.1) | 9.3 (7.9-10.6) | 8.6 (6.1-11) | 7.6 (5.9-9.3) | uncharacterized LOC100653132 |
| 204646_at | DPYD | 2.502 |  | -0.031 |  | 6.3 (4.1-8.5) | 7.9 (7.1-8.6) | 6.1 (4.6-7.7) | 8.2 (7.5-8.8) | 7.2 (5.3-9.2) | 6.9 (4.9-8.8) | dihydropyrimidine dehydrogenase |
| 238462_at | UBASH3B | 2.315 |  | -0.031 |  | 4.3 (2.5-6) | 5.6 (3.6-7.6) | 4.1 (2.8-5.4) | 5.9 (3.9-7.9) | 4.8 (3.1-6.5) | 5.2 (3.2-7.1) | ubiquitin associated and SH3 domain containing B |
| 212063_at | CD44 | 3.519 |  | -0.030 |  | 8.3 (6.3-10.3) | 7.8 (4.6-10.9) | 6.5 (4.3-8.7) | 9.1 (6.5-11.7) | 7.2 (4.2-10.2) | 7.3 (4.3-10.3) | CD44 molecule (Indian blood group) |
| 205269_at | LCP2 | 1.584 |  | -0.029 |  | 5.4 (3.5-7.4) | 4.9 (3-6.9) | 3.7 (2.6-4.7) | 6.4 (4.8-8) | 4.2 (2.6-5.8) | 5.8 (3.8-7.8) | lymphocyte cytosolic protein 2 (SH2 domain containing leukocyte protein of 76kDa) |
| 224588_at | XIST | 3.276 |  | -0.029 | -0.052 | 6.6 (3.5-9.7) | 7.8 (4.8-10.8) | 5.4 (3.2-7.5) | 7.4 (4.6-10.1) | 5.6 (3.1-8) | 9 (6.2-11.8) | X (inactive)-specific transcript (non-protein coding) |
| 219874_at | SLC12A8 | 1.843 |  | -0.027 |  | 5.5 (3.9-7.2) | 5.8 (4.4-7.2) | 4.9 (4.6-5.2) | 6.8 (5.1-8.6) | 5.5 (4.2-6.8) | 6.3 (4.6-8) | solute carrier family 12 (potassium/chloride transporters), member 8 |
| 222108_at | AMIGO2 | 2.539 |  | -0.024 |  | 5.5 (2.9-8) | 5 (3.2-6.8) | 4.8 (2.7-6.9) | 5.4 (3.7-7.1) | 4.8 (2.7-7) | 4.7 (2.6-6.7) | adhesion molecule with Ig-like domain 2 |
| 203964_at | NMI | 2.641 |  | -0.024 |  | 8 (6-10) | 8.7 (6.4-11) | 7.2 (5.1-9.2) | 9.7 (8.5-10.8) | 8 (5.6-10.3) | 8.9 (7.1-10.7) | N-myc (and STAT) interactor |
| 214012_at | ERAP1 | 1.206 |  | -0.023 |  | 5.5 (4.8-6.2) | 6.1 (4.5-7.8) | 4.6 (3.8-5.4) | 6.7 (5.8-7.6) | 5.8 (4.7-6.9) | 6.2 (4.9-7.5) | endoplasmic reticulum aminopeptidase 1 |
| 200905_x_at | HLA-E | 1.424 |  | -0.023 |  | 10.2 (8.8-11.7) | 11.3 (10.7-11.9) | 9.8 (8.8-10.8) | 11.7 (11.2-12.2) | 10.6 (8.9-12.2) | 11.4 (11-11.8) | major histocompatibility complex, class I, E |
| 204205_at | APOBEC3G | 1.766 |  | -0.022 |  | 8.3 (5.9-10.6) | 8.5 (6.9-10.1) | 7.6 (5.9-9.3) | 9.8 (8.8-10.7) | 8.6 (7.1-10.1) | 8.8 (6.7-11) | apolipoprotein B mRNA editing enzyme, catalytic polypeptide-like 3G |
| 203767_s_at | STS | 1.961 |  | -0.021 |  | 7.2 (4.4-9.9) | 8 (7-9) | 7 (5.2-8.7) | 9 (7.9-10.1) | 8.5 (7.4-9.6) | 8 (5.9-10) | steroid sulfatase (microsomal), isozyme S |
| 209031_at | CADM1 | 2.899 |  | -0.020 |  | 8.1 (5.7-10.6) | 9.1 (6.4-11.7) | 7.7 (5.8-9.7) | 8.7 (5.9-11.4) | 8.4 (5.5-11.2) | 9.4 (7.3-11.4) | cell adhesion molecule 1 |
| 213375_s_at | N4BP2L1 | 1.697 |  | -0.019 |  | 6.4 (5.3-7.4) | 7.4 (6.2-8.7) | 5.8 (4.7-6.8) | 7.5 (6.5-8.6) | 6.7 (5.1-8.4) | 7.2 (6.4-8) | NEDD4 binding protein 2-like 1 |
| 206687_s_at | PTPN6 | 1.585 |  | -0.018 |  | 8.9 (7.4-10.3) | 9.1 (7.8-10.5) | 8 (7-9.1) | 9.9 (8.8-11) | 8.2 (6.9-9.5) | 9.4 (8.3-10.6) | protein tyrosine phosphatase, non-receptor type 6 |
| 209205_s_at | LMO4 | 1.216 |  | -0.018 |  | 8.6 (7.7-9.5) | 9.7 (8.8-10.7) | 8 (7.4-8.7) | 9.8 (9.2-10.5) | 8.9 (7.9-9.9) | 10.1 (9.2-11) | LIM domain only 4 |
| 226837_at | SPRED1 | 1.419 |  | -0.018 |  | 6.1 (4.4-7.7) | 7.5 (6.5-8.5) | 5.5 (4-7) | 7.8 (7.2-8.3) | 6.8 (5.4-8.1) | 7.7 (7.2-8.2) | sprouty-related, EVH1 domain containing 1 |
| 213060_s_at | CHI3L2 | 2.544 |  | -0.017 |  | 6.7 (4.9-8.6) | 6 (4-8) | 7 (5.1-8.9) | 7.1 (5-9.1) | 6.8 (5.2-8.4) | 6.8 (4.4-9.3) | chitinase 3-like 2 |
| 202499_s_at | SLC2A3 | 1.423 |  | -0.017 |  | 5.4 (4-6.7) | 5.7 (4.4-7) | 4.9 (4.2-5.7) | 6.4 (5.3-7.6) | 4.9 (4.1-5.7) | 5.9 (4.6-7.2) | solute carrier family 2 (facilitated glucose transporter), member 3 |
| 213888_s_at | TRAF3IP3 | 2.058 |  | -0.015 |  | 7.6 (5.7-9.5) | 7.5 (5.5-9.5) | 7 (5.3-8.7) | 8.6 (7-10.2) | 7.7 (5.8-9.6) | 8.3 (6.6-10) | TRAF3 interacting protein 3 |
| 205518_s_at | CMAHP | 1.243 |  | -0.014 |  | 5.4 (3.8-6.9) | 5.7 (4.4-7) | 4.2 (3.7-4.7) | 6.5 (5.3-7.6) | 4.9 (3.8-5.9) | 6.2 (4.9-7.6) | cytidine monophospho-N-acetylneuraminic acid hydroxylase, pseudogene |
| 225589_at | SH3RF1 | 1.400 |  | -0.014 |  | 4.6 (3.1-6.1) | 6.3 (5.2-7.4) | 4.3 (3.3-5.2) | 6.6 (5.5-7.8) | 5.6 (3.5-7.6) | 6.1 (4.7-7.5) | SH3 domain containing ring finger 1 |
| 203132_at | RB1 | 1.466 |  | -0.012 |  | 8 (6.7-9.3) | 9.7 (8.7-10.7) | 8.2 (7.2-9.2) | 9.9 (9-10.7) | 8.8 (7.6-10) | 9.1 (7.8-10.5) | retinoblastoma 1 |
| 202733_at | P4HA2 | 2.241 |  | -0.012 |  | 6.9 (4.9-8.9) | 6.3 (4.8-7.8) | 5.4 (4.7-6.1) | 7 (5.2-8.8) | 6.6 (4.9-8.3) | 7.5 (5.5-9.5) | prolyl 4-hydroxylase, alpha polypeptide II |
| 203275_at | IRF2 | 1.239 |  | -0.012 |  | 7.7 (6.7-8.8) | 8.6 (8.2-8.9) | 7.3 (6.3-8.2) | 8.8 (8.5-9.2) | 8.1 (6.7-9.4) | 8.5 (8.2-8.8) | interferon regulatory factor 2 |
| 215118_s_at | IGHA1 | 1.986 |  | -0.011 |  | 7.1 (4.5-9.6) | 5.7 (4.2-7.1) | 5.1 (4.6-5.6) | 7.3 (4.7-9.9) | 6.3 (3.8-8.9) | 7 (4.8-9.3) | Immunoglobulin heavy constant alpha 1 |
| 219667_s_at | BANK1 | 2.581 |  | -0.008 |  | 5.5 (3.9-7.1) | 6.6 (4.1-9.2) | 5.6 (3.6-7.6) | 6.7 (4.5-8.8) | 4.6 (3.9-5.3) | 5.7 (4-7.4) | B-cell scaffold protein with ankyrin repeats 1 |
| 219304_s_at | PDGFD | 2.490 |  | -0.008 |  | 6.3 (3.8-8.7) | 5.4 (3.8-7.1) | 6.3 (4.2-8.4) | 5.8 (4-7.6) | 5.6 (3.8-7.5) | 6.3 (4.2-8.5) | platelet derived growth factor D |
| 225541_at | RPL22L1 | 1.119 |  | -0.007 |  | 11 (10-11.9) | 11.7 (10.4-12.9) | 10.6 (9.9-11.4) | 12 (11.3-12.8) | 10.9 (10.3-11.4) | 11.6 (10.3-13) | ribosomal protein L22-like 1 |
| 1553856_s_at | P2RY10 | 1.502 |  | -0.006 |  | 7.3 (6.1-8.6) | 7 (5.2-8.8) | 5.9 (5-6.8) | 7.7 (6.2-9.1) | 6.5 (5.5-7.5) | 7.8 (6.3-9.3) | purinergic receptor P2Y, G-protein coupled, 10 |
| 219563_at | LINC00341 | 1.636 |  | -0.005 |  | 9.7 (8.5-10.8) | 10 (8.6-11.3) | 8.8 (7.8-9.7) | 10.4 (9.4-11.4) | 9 (7.4-10.5) | 10.3 (9.2-11.4) | long intergenic non-protein coding RNA 341 |
| 203216_s_at | MYO6 | 2.470 |  | -0.004 |  | 6.4 (4.4-8.4) | 6.6 (4.5-8.7) | 5.4 (3.7-7.1) | 6.8 (4.7-8.8) | 5.5 (3.5-7.4) | 6.8 (4.8-8.7) | myosin VI |
| 221601_s_at | FAIM3 | 2.091 |  | -0.002 |  | 7.6 (5.9-9.2) | 8.2 (5.9-10.6) | 6.5 (5.6-7.5) | 8.8 (6.9-10.7) | 7.2 (5.4-9) | 7.9 (5.8-9.9) | Fas apoptotic inhibitory molecule 3 |
| 209474_s_at | ENTPD1 | 2.283 |  | -0.002 |  | 6.5 (4.7-8.3) | 5.8 (3.9-7.7) | 4.9 (4-5.7) | 6.4 (4.4-8.4) | 5.5 (3.6-7.3) | 5.5 (3.5-7.5) | ectonucleoside triphosphate diphosphohydrolase 1 |
| 227189_at | CPNE5 | 2.453 |  | -0.002 |  | 6.7 (5-8.3) | 6 (4.5-7.5) | 6.4 (4.8-8.1) | 7.1 (5.1-9) | 6.2 (4.2-8.1) | 7.6 (5.8-9.4) | copine V |
| 221727_at | SUB1 | 1.303 |  | -0.001 |  | 8.9 (7.5-10.4) | 10.5 (9.4-11.6) | 8.6 (7.4-9.8) | 10.2 (9-11.4) | 9.6 (8.2-11) | 9.7 (8.5-11) | SUB1 homolog (S. cerevisiae) |
| 1554240_a_at | ITGAL | 2.289 |  | -0.001 |  | 7.5 (6.1-8.9) | 7.4 (5.3-9.5) | 7.3 (5.8-8.9) | 8.1 (6.1-10.1) | 6.9 (4.4-9.4) | 7.6 (5.9-9.2) | integrin, alpha L (antigen CD11A (p180), lymphocyte function-associated antigen 1; alpha polypeptide) |
| 202478_at | TRIB2 | 2.030 |  | -0.001 |  | 7.7 (5.9-9.6) | 7.7 (6.2-9.3) | 7.6 (6-9.2) | 8.7 (7.4-10.1) | 8.7 (7.2-10.2) | 6.9 (5.5-8.3) | tribbles homolog 2 (Drosophila) |
| 209829_at | FAM65B | 1.591 |  | 0.000 |  | 7.6 (5.6-9.7) | 6.1 (4-8.3) | 6.4 (4.6-8.1) | 7.9 (5.9-9.9) | 6.8 (4.9-8.7) | 6.6 (4.3-9) | family with sequence similarity 65, member B |
| 220330_s_at | SAMSN1 | 2.500 |  |  | -0.042 | 5.7 (3.8-7.6) | 4.2 (2.7-5.8) | 4.8 (3.5-6.1) | 4.8 (2.8-6.7) | 4.1 (3-5.3) | 6.2 (3.7-8.7) | SAM domain, SH3 domain and nuclear localization signals 1 |
| 204249_s_at | LMO2 | 2.362 |  |  | -0.035 | 6.7 (4.4-9) | 6.2 (4.7-7.8) | 5.8 (4.7-7) | 6.9 (4.8-9) | 5.2 (4.5-5.8) | 7.5 (5-10) | LIM domain only 2 (rhombotin-like 1) |
| 205488_at | GZMA | 1.744 |  |  | -0.028 | 4.3 (4.3-4.4) | 5.2 (3.3-7.1) | 4.3 (4.3-4.4) | 5.1 (3.4-6.8) | 4.3 (4.1-4.5) | 5.3 (3.3-7.2) | granzyme A (granzyme 1, cytotoxic T-lymphocyte-associated serine esterase 3) |
| 223220_s_at | PARP9 | 1.808 |  |  | -0.023 | 7.5 (6.1-8.9) | 7.1 (5.4-8.8) | 6.4 (5.4-7.4) | 7.4 (5.7-9) | 5.8 (5.1-6.5) | 8 (6.6-9.5) | poly (ADP-ribose) polymerase family, member 9 |
| 225415_at | DTX3L | 1.917 |  |  | -0.022 | 7.6 (5.8-9.4) | 7.6 (5.4-9.8) | 6.1 (4.7-7.6) | 7.8 (6-9.6) | 6 (4.5-7.6) | 8.5 (7.1-9.9) | deltex 3-like (Drosophila) |
| 201739_at | SGK1 | 2.373 |  |  | -0.019 | 9.7 (7.7-11.8) | 8.9 (7.2-10.6) | 8.6 (7.6-9.6) | 9.4 (7.4-11.4) | 7.6 (6.3-8.9) | 10.5 (8.9-12.1) | serum/glucocorticoid regulated kinase 1 |
| 1554733_at | LOC728175 | 2.302 |  |  | -0.018 | 4.7 (3.3-6.1) | 5.4 (3.2-7.6) | 3.9 (3.1-4.7) | 4.8 (3.2-6.4) | 3.6 (3.2-4.1) | 5.6 (3.6-7.6) | uncharacterized LOC728175 |
| 1556096_s_at | UNC13C | 3.044 |  |  | -0.012 | 7.3 (4.5-10.1) | 5.7 (3.1-8.4) | 6 (3.5-8.5) | 6.1 (3.5-8.8) | 5.1 (2.7-7.4) | 8.3 (5.7-10.9) | unc-13 homolog C (C. elegans) |
| 209101_at | CTGF | 1.820 |  |  | -0.010 | 3.6 (3.4-3.7) | 4.7 (2.7-6.6) | 3.6 (3.5-3.7) | 4.5 (2.8-6.2) | 3.5 (3.3-3.7) | 4.5 (2.5-6.5) | connective tissue growth factor |
| 227711_at | GTSF1 | 3.212 |  |  | -0.009 | 9.3 (6.6-12) | 9.3 (6.5-12.2) | 8.5 (5.8-11.2) | 9.2 (6.9-11.6) | 8 (5.4-10.7) | 10.1 (7.9-12.3) | gametocyte specific factor 1 |
| 227856_at | C4orf32 | 2.393 |  |  | -0.007 | 7.9 (5.5-10.4) | 9.2 (8.3-10.1) | 7.7 (5.7-9.7) | 8.9 (8.2-9.7) | 7.5 (5.4-9.7) | 9.5 (8.5-10.5) | chromosome 4 open reading frame 32 |
| 209480_at | HLA-DQB1 | 2.678 |  |  | -0.004 | 5.7 (3.4-8) | 5.9 (3.8-8.1) | 5.9 (3.9-7.9) | 5.9 (3.7-8.1) | 4.7 (3.6-5.8) | 6.8 (4.3-9.4) | major histocompatibility complex, class II, DQ beta 1 |
| 203305_at | F13A1 | 1.846 |  |  | -0.001 | 5.5 (4.3-6.7) | 5.8 (4.1-7.5) | 5.4 (4.4-6.5) | 5.4 (3.9-7) | 5.3 (4.1-6.5) | 5.6 (3.9-7.4) | coagulation factor XIII, A1 polypeptide |
| 230983_at | FAM129C | 2.021 |  |  | 0.000 | 6.6 (5.1-8.1) | 7.3 (4.9-9.7) | 7.3 (5.7-8.8) | 7.2 (5.3-9.1) | 7.6 (5.4-9.8) | 6.2 (5-7.5) | family with sequence similarity 129, member C |
| 221760_at | MAN1A1 | 2.345 |  |  | 0.001 | 10.3 (9.4-11.2) | 9.9 (7.4-12.3) | 9.7 (7.9-11.5) | 10 (7.9-12.1) | 11.1 (9.8-12.4) | 9.8 (8-11.5) | mannosidase, alpha, class 1A, member 1 |
| 206108_s_at | SRSF6 | 1.547 |  |  | 0.004 | 8.6 (7.5-9.7) | 7.9 (6.5-9.4) | 8.9 (8-9.7) | 8 (6.8-9.3) | 9 (7.9-10) | 7.3 (6.2-8.4) | serine/arginine-rich splicing factor 6 |
| 225763_at | RCSD1 | 2.781 |  |  | 0.005 | 9.2 (7.7-10.8) | 10.1 (9-11.3) | 9.9 (8.8-11.1) | 10.1 (9-11.2) | 10.6 (10-11.2) | 9.3 (8.1-10.5) | RCSD domain containing 1 |
| 220051_at | PRSS21 | 1.930 |  |  | 0.006 | 6.5 (4.3-8.7) | 6 (3.9-8) | 7.5 (5.4-9.5) | 5.8 (4-7.6) | 7.1 (4.9-9.3) | 4.7 (4.5-4.8) | protease, serine, 21 (testisin) |
| 212442_s_at | CERS6 | 2.159 |  |  | 0.007 | 7.2 (4.8-9.6) | 7.5 (5.1-9.9) | 7.4 (5.2-9.6) | 7.7 (5.6-9.8) | 8.6 (6.4-10.7) | 6.7 (4.5-9) | ceramide synthase 6 |
| 226905_at | FAM101B | 2.573 |  |  | 0.009 | 7.1 (4.5-9.7) | 6.6 (4.1-9.1) | 7.2 (5.3-9.1) | 6.2 (4-8.4) | 7.8 (5.3-10.4) | 6.6 (3.4-9.7) | family with sequence similarity 101, member B |
| 217979_at | TSPAN13 | 2.705 |  |  | 0.012 | 9.3 (7.7-11) | 9.4 (7.1-11.7) | 9.6 (8.2-10.9) | 9.7 (7.8-11.6) | 10.3 (9.4-11.3) | 7.6 (5.1-10.1) | tetraspanin 13 |
| 219335_at | ARMCX5 | 1.872 |  |  | 0.026 | 4.5 (3-6.1) | 5.6 (3.8-7.5) | 5.2 (3.5-6.8) | 5.2 (3.6-6.8) | 7 (5.7-8.4) | 4.2 (2.9-5.6) | armadillo repeat containing, X-linked 5 |
| 216379_x_at | CD24 | 3.195 |  |  | 0.040 | 6.2 (3.1-9.3) | 7.6 (4.3-10.8) | 7.2 (4.2-10.2) | 7.2 (4.3-10.2) | 8.2 (4.7-11.7) | 6.2 (3.2-9.2) | CD24 molecule |
| 219737_s_at | PCDH9 | 2.601 |  | 0.000 |  | 7 (4.3-9.7) | 7 (4.9-9.2) | 7.7 (5.5-9.9) | 6.5 (4.9-8.1) | 7.8 (5.4-10.2) | 6 (4.7-7.3) | protocadherin 9 |
| 206018_at | FOXG1 | 1.686 |  | 0.000 |  | 4.2 (2.4-6) | 3.8 (3.1-4.5) | 4.6 (3-6.2) | 3.6 (3.5-3.7) | 3.8 (3.1-4.5) | 3.5 (3.4-3.7) | forkhead box G1 |
| 1553613_s_at | FOXC1 | 1.382 |  | 0.000 |  | 4.4 (3-5.8) | 3.8 (3.4-4.1) | 4.7 (3.5-6) | 3.9 (3.5-4.3) | 4.5 (3.1-5.8) | 3.7 (3.4-4.1) | forkhead box C1 |
| 1255_g_at | GUCA1A | 1.958 |  | 0.000 |  | 4.3 (2.1-6.5) | 3.3 (3.3-3.4) | 4.3 (2.4-6.1) | 3.3 (3.2-3.4) | 3.3 (3.2-3.5) | 3.3 (3.3-3.4) | guanylate cyclase activator 1A (retina) |
| 200771_at | LAMC1 | 1.769 |  | 0.001 |  | 7 (5.5-8.5) | 7.4 (5.4-9.3) | 8.3 (8-8.6) | 6.6 (4.7-8.4) | 8.2 (6.3-10) | 6.1 (4.5-7.7) | laminin, gamma 1 (formerly LAMB2) |
| 238605_at | NOL4 | 1.774 |  | 0.001 |  | 3.9 (2.2-5.6) | 3.2 (3.1-3.3) | 4.6 (3-6.2) | 3.2 (3.1-3.3) | 4.3 (2.6-6) | 3.2 (3.2-3.3) | nucleolar protein 4 |
| 209806_at | HIST1H2BK | 1.830 |  | 0.001 |  | 11.3 (9.6-13.1) | 10.9 (9.6-12.3) | 11 (9.5-12.4) | 10.1 (8.7-11.4) | 11.4 (9.8-13) | 10.6 (8.8-12.4) | histone cluster 1, H2bk |
| 208579_x_at | H2BFS | 1.513 |  | 0.002 |  | 10.9 (9.4-12.4) | 10.5 (9.5-11.5) | 10.7 (9.6-11.9) | 9.8 (8.9-10.7) | 11.1 (9.8-12.3) | 10.3 (8.9-11.7) | H2B histone family, member S (pseudogene) /// histone cluster 1, H2bk |
| 218454_at | PLBD1 | 1.956 |  | 0.002 |  | 5.4 (4-6.8) | 5.1 (3.7-6.5) | 5.8 (4.2-7.4) | 4.5 (4.4-4.7) | 5.5 (3.7-7.3) | 4.6 (4.3-4.8) | phospholipase B domain containing 1 |
| 203504_s_at | ABCA1 | 1.915 |  | 0.002 |  | 5.1 (3.6-6.5) | 5.3 (3.7-6.9) | 6.1 (4.6-7.7) | 4.8 (3.6-6) | 6.1 (4.6-7.7) | 5.4 (3.8-7.1) | ATP-binding cassette, sub-family A (ABC1), member 1 |
| 204059_s_at | ME1 | 1.848 |  | 0.005 |  | 6.7 (3.9-9.4) | 5.9 (3.9-7.9) | 7 (4.5-9.4) | 5.2 (3.3-7.1) | 4.8 (2.9-6.6) | 6.3 (3.9-8.6) | malic enzyme 1, NADP(+)-dependent, cytosolic |
| 240091_at | PSMA8 | 2.317 |  | 0.007 |  | 5.4 (3.3-7.5) | 4.5 (3.6-5.4) | 5.9 (3.9-8) | 4.3 (3.5-5.1) | 4.5 (3.7-5.2) | 4.7 (3.5-6) | proteasome (prosome, macropain) subunit, alpha type, 8 |
| 201540_at | FHL1 | 1.617 |  | 0.008 |  | 7.2 (4.9-9.5) | 7.6 (4.9-10.2) | 8.7 (6.5-11) | 7.1 (4.8-9.4) | 9.2 (6.6-11.9) | 7.2 (5.2-9.3) | four and a half LIM domains 1 |
| 206637_at | P2RY14 | 1.631 |  | 0.009 |  | 3.7 (2.2-5.3) | 3.2 (2.7-3.6) | 3.7 (2.4-5.1) | 3.2 (2.8-3.6) | 3.7 (2.2-5.3) | 3.3 (2.8-3.7) | purinergic receptor P2Y, G-protein coupled, 14 |
| 204730_at | RIMS3 | 1.668 |  | 0.011 |  | 8.3 (7-9.6) | 6.9 (5.2-8.6) | 8.8 (7.6-9.9) | 7 (5.7-8.3) | 8 (6.1-9.9) | 7.4 (6.2-8.7) | regulating synaptic membrane exocytosis 3 |
| 235773_at | ZIK1 | 1.541 |  | 0.011 |  | 6.6 (5-8.2) | 5.2 (4.2-6.1) | 6.4 (5-7.7) | 4.8 (4.2-5.3) | 6 (4.4-7.6) | 5.6 (4.5-6.7) | zinc finger protein interacting with K protein 1 homolog (mouse) |
| 213050_at | COBL | 1.820 |  | 0.015 |  | 6 (3.8-8.2) | 4.4 (4-4.9) | 5.6 (4-7.2) | 4.2 (3.9-4.5) | 5.1 (3.3-6.8) | 5 (3.6-6.4) | cordon-bleu homolog (mouse) |
| 237206_at | MYOCD | 2.716 |  | 0.016 |  | 4.2 (2.7-5.7) | 3.7 (3.4-4) | 4.1 (2.8-5.4) | 3.6 (3.4-3.9) | 4.2 (2.7-5.7) | 3.8 (3.5-4.1) | myocardin |
| 222774_s_at | NETO2 | 2.306 |  | 0.018 |  | 8.1 (7.4-8.8) | 6.8 (5-8.7) | 7.8 (6.4-9.1) | 7.1 (5.7-8.6) | 7.4 (5.9-8.9) | 6.8 (5.2-8.5) | neuropilin (NRP) and tolloid (TLL)-like 2 |
| 231851_at | RAVER2 | 1.840 |  | 0.018 |  | 6.3 (4.8-7.9) | 6.7 (5.2-8.3) | 6.6 (5-8.2) | 5.7 (4.2-7.3) | 6.2 (4.4-7.9) | 6.3 (4.8-7.8) | ribonucleoprotein, PTB-binding 2 |
| 202806_at | DBN1 | 1.879 |  | 0.019 |  | 7.8 (6.1-9.5) | 7.5 (5.7-9.2) | 8.4 (7.2-9.6) | 6.6 (5.2-8) | 7.8 (6.2-9.4) | 6.7 (5-8.4) | drebrin 1 |
| 215767_at | ZNF804A | 1.807 |  | 0.019 |  | 7.3 (5.1-9.5) | 6.7 (5.5-7.9) | 8.1 (6.5-9.6) | 6 (4.6-7.4) | 7 (4.7-9.3) | 6.5 (5.3-7.7) | zinc finger protein 804A |
| 219255_x_at | IL17RB | 2.515 |  | 0.019 |  | 7.3 (5.6-9.1) | 5.9 (4.6-7.2) | 7.4 (5.7-9) | 5.5 (4.6-6.4) | 6.8 (5.1-8.4) | 5.4 (4.8-6.1) | interleukin 17 receptor B |
| 228988_at | ZNF711 | 2.440 |  | 0.021 |  | 5.1 (3-7.3) | 5.5 (3.5-7.6) | 6.5 (4.6-8.3) | 5.1 (3.4-6.7) | 6.2 (4.2-8.2) | 6.2 (4-8.3) | zinc finger protein 711 |
| 225532_at | CABLES1 | 2.071 |  | 0.022 |  | 8.7 (6.8-10.6) | 7.6 (5.8-9.4) | 8.9 (7.3-10.5) | 7.4 (6.1-8.8) | 8.4 (6.7-10.1) | 8.9 (7.2-10.5) | Cdk5 and Abl enzyme substrate 1 |
| 41037_at | TEAD4 | 2.115 |  | 0.023 |  | 6 (4.4-7.5) | 5.3 (3.8-6.7) | 6.3 (4.7-7.8) | 4.6 (3.7-5.6) | 5.7 (4.2-7.3) | 5.3 (4.1-6.5) | TEA domain family member 4 |
| 212761_at | TCF7L2 | 1.914 |  | 0.026 |  | 5.4 (3.8-7.1) | 5.8 (4.2-7.4) | 6.2 (5-7.5) | 5.4 (3.9-6.9) | 6 (4.4-7.6) | 5.2 (3.7-6.7) | transcription factor 7-like 2 (T-cell specific, HMG-box) |
| 204891_s_at | LCK | 2.821 |  | 0.026 |  | 7.8 (5.2-10.3) | 6.1 (4.3-8) | 8.4 (6.1-10.7) | 6.7 (4.5-9) | 7.1 (4.6-9.6) | 7.8 (5-10.6) | lymphocyte-specific protein tyrosine kinase |
| 231579_s_at | TIMP2 | 1.854 |  | 0.030 |  | 7.3 (4.8-9.9) | 7.6 (4.7-10.4) | 7.8 (5.6-9.9) | 6.4 (3.7-9.1) | 8.1 (5.2-10.9) | 7.5 (4.7-10.3) | TIMP metallopeptidase inhibitor 2 |
| 213436_at | CNR1 | 3.058 |  | 0.031 |  | 6.5 (3.6-9.4) | 5 (2.9-7.2) | 7.4 (4.6-10.1) | 4.8 (3.4-6.2) | 6.5 (3.6-9.4) | 5.4 (3.3-7.4) | cannabinoid receptor 1 (brain) |
| 228297_at |  | 2.216 |  | 0.033 |  | 5.1 (2.6-7.5) | 4.4 (2.6-6.2) | 5.3 (3.2-7.4) | 3.7 (2.8-4.7) | 4.9 (2.5-7.3) | 4.9 (2.9-6.8) |  |
| 218858_at | DEPTOR | 3.380 |  | 0.033 |  | 8.4 (6.1-10.6) | 7.8 (5-10.6) | 9.9 (8.1-11.7) | 7.3 (4.7-9.9) | 8.9 (6-11.7) | 9 (6.5-11.4) | DEP domain containing MTOR-interacting protein |
| 207039_at | CDKN2A | 1.985 |  | 0.034 |  | 7.6 (6.3-9) | 6.8 (4.8-8.8) | 8.3 (7.1-9.5) | 6.2 (4.9-7.6) | 7.3 (5.6-9) | 7.2 (5.8-8.6) | cyclin-dependent kinase inhibitor 2A |
| 229656_s_at | EML6 | 2.420 |  | 0.035 |  | 8.4 (6.4-10.4) | 7.4 (6.5-8.3) | 9.2 (7.6-10.8) | 6.9 (5.8-8) | 8.1 (6.4-9.9) | 7.2 (5.9-8.4) | echinoderm microtubule associated protein like 6 |
| 224918_x_at | MGST1 | 3.501 |  | 0.036 |  | 8 (5-11.1) | 7.4 (4.4-10.4) | 9.1 (6.4-11.9) | 7.3 (4.7-9.9) | 7.5 (4.6-10.5) | 7.2 (4.3-10.1) | microsomal glutathione S-transferase 1 |
| 202016_at | MEST | 2.593 |  | 0.038 | 0.003 | 8.8 (6.8-10.9) | 7.5 (4.9-10) | 10.3 (9.5-11.2) | 7.5 (5.4-9.6) | 9 (7.1-10.9) | 6.8 (5.1-8.4) | mesoderm specific transcript homolog (mouse) |
| 220595_at | PDZRN4 | 2.233 |  | 0.041 |  | 4.1 (2.4-5.8) | 3.5 (3.1-4) | 5.2 (3.3-7.1) | 3.4 (3.2-3.5) | 4.2 (2.7-5.6) | 3.7 (3-4.4) | PDZ domain containing ring finger 4 |
| 208999_at | SEPT8 | 1.520 |  | 0.042 |  | 7.6 (6.7-8.5) | 7.7 (6.5-8.9) | 7.9 (6.9-8.9) | 6.6 (5.6-7.5) | 7.4 (6.3-8.6) | 7.3 (6.2-8.4) | septin 8 |
| 226190_at | MAP3K13 | 2.685 |  | 0.063 |  | 9.4 (7.8-11.1) | 8.5 (6.4-10.5) | 9.1 (7.4-10.9) | 7.3 (5.1-9.5) | 9 (7-11) | 8.1 (5.7-10.6) | mitogen-activated protein kinase kinase kinase 13 |
| 204992_s_at | PFN2 | 2.331 |  | 0.064 | 0.053 | 7.8 (6.1-9.5) | 6.6 (5-8.2) | 9.5 (8.8-10.1) | 6.7 (5.4-8) | 8.9 (7.4-10.3) | 6 (5.2-6.7) | profilin 2 |
| 212915_at | PDZRN3 | 2.240 |  | 0.064 |  | 4.5 (2.4-6.6) | 3.1 (3-3.2) | 5 (2.9-7) | 3.1 (3-3.2) | 3.8 (2.4-5.1) | 3.2 (2.9-3.6) | PDZ domain containing ring finger 3 |
| 225129_at | CPNE2 | 2.066 |  | 0.066 |  | 7.4 (6.1-8.6) | 7.1 (5.4-8.8) | 7.7 (6.5-8.9) | 6.4 (5.2-7.5) | 7 (5.4-8.6) | 6.4 (5.1-7.7) | copine II |
| 201839_s_at | EPCAM | 2.028 |  | 0.073 |  | 6.8 (4-9.7) | 5.1 (3.5-6.8) | 7.7 (5.3-10.1) | 4.1 (3.6-4.6) | 5 (3-7) | 5 (3.1-6.9) | epithelial cell adhesion molecule |
| 231984_at | MTAP | 2.176 | 0.001 | 0.048 |  | 8.3 (7.9-8.7) | 6.6 (5-8.2) | 8.5 (8.1-8.9) | 6.2 (4.7-7.7) | 7.2 (5.6-8.8) | 7.8 (6.7-8.8) | methylthioadenosine phosphorylase |
| 230673_at | PKHD1L1 | 2.334 | 0.001 |  |  | 6.7 (5.1-8.4) | 4.8 (3.3-6.3) | 6.3 (4.3-8.2) | 5.6 (4.2-7.1) | 5.7 (3.7-7.7) | 6.6 (4.7-8.4) | polycystic kidney and hepatic disease 1 (autosomal recessive)-like 1 |
| 229829_at | LINC00526 | 1.481 | 0.002 |  |  | 7.8 (7-8.5) | 6.4 (5.7-7.1) | 7.4 (6.6-8.2) | 6.5 (5.7-7.2) | 7.5 (6.4-8.5) | 7.3 (6.2-8.4) | long intergenic non-protein coding RNA 526 |
| 219518_s_at | ELL3 | 2.274 | 0.002 |  |  | 8 (5.2-10.8) | 6.5 (4.4-8.6) | 8.5 (5.9-11.1) | 6.9 (4.8-9) | 7.2 (4.8-9.7) | 7.5 (4.7-10.3) | elongation factor RNA polymerase II-like 3 |
| 206641_at | TNFRSF17 | 2.294 | 0.003 |  |  | 11.3 (9.7-13) | 10 (7.1-12.9) | 10.9 (9.7-12.1) | 9.9 (7-12.7) | 10.8 (8.8-12.8) | 10.6 (8.4-12.8) | tumor necrosis factor receptor superfamily, member 17 |
| 204141_at | TUBB2A | 1.986 | 0.003 |  |  | 9.4 (7.6-11.2) | 7.5 (6.6-8.4) | 8.9 (6.9-10.9) | 8.2 (6.8-9.7) | 9 (7.5-10.4) | 8.3 (6.9-9.7) | tubulin, beta 2A class IIa |
| 210715_s_at | SPINT2 | 1.841 | 0.004 |  |  | 9.8 (8.8-10.8) | 7.2 (5.4-9) | 7.8 (6-9.7) | 7.6 (6.4-8.9) | 7.3 (5.5-9.2) | 8.2 (6.3-10.1) | serine peptidase inhibitor, Kunitz type, 2 |
| 234250_at | LOC100506405 | 1.407 | 0.006 |  |  | 5.4 (4-6.8) | 4.5 (4.3-4.7) | 5.3 (4.1-6.6) | 4.5 (4.4-4.7) | 5 (3.8-6.2) | 4.6 (4.5-4.8) | uncharacterized LOC100506405 |
| 231391_at | CTXN3 | 1.798 | 0.007 |  |  | 4.2 (2.3-6.2) | 3.3 (3.2-3.4) | 4.1 (2.4-5.8) | 3.4 (3.3-3.5) | 3.4 (3.2-3.5) | 3.3 (3.2-3.4) | cortexin 3 |
| 238429_at | TMEM71 | 2.010 | 0.008 |  |  | 5.2 (3-7.4) | 3.6 (3.4-3.7) | 4.7 (2.8-6.5) | 3.4 (3.3-3.6) | 4.4 (2.3-6.5) | 4 (3-5.1) | transmembrane protein 71 |
| 225060_at | LRP11 | 2.254 | 0.008 |  |  | 7.2 (5.5-8.9) | 5.6 (4.1-7.1) | 6.9 (5.2-8.5) | 6.3 (4.6-8) | 5.8 (3.6-8) | 6 (4.6-7.5) | low density lipoprotein receptor-related protein 11 |
| 218872_at | TESC | 1.693 | 0.009 |  |  | 8.3 (7.8-8.9) | 6.7 (5.6-7.7) | 7.9 (7.1-8.7) | 6.5 (5.5-7.6) | 6.9 (5.6-8.3) | 7.5 (6.6-8.3) | tescalcin |
| 206163_at | MAB21L1 | 1.450 | 0.010 |  |  | 4.3 (3.3-5.4) | 3.5 (3.3-3.7) | 4.7 (3.3-6) | 3.5 (3.4-3.7) | 3.8 (3-4.6) | 3.6 (3.4-3.7) | mab-21-like 1 (C. elegans) |
| 210427_x_at | ANXA2 | 2.115 | 0.010 |  |  | 12.1 (11.4-12.8) | 11.3 (10-12.6) | 11.1 (9.6-12.7) | 11.5 (10.2-12.7) | 11.5 (10.5-12.4) | 10.6 (8.5-12.7) | annexin A2 |
| 1552531_a_at | NLRP11 | 2.461 | 0.011 |  |  | 9.1 (7.1-11.2) | 7.2 (5.7-8.7) | 7.8 (5.5-10) | 6.7 (5.7-7.8) | 8 (5.9-10) | 8.6 (6.7-10.6) | NLR family, pyrin domain containing 11 |
| 201462_at | SCRN1 | 2.529 | 0.011 | 0.025 | 0.008 | 9.1 (7.2-11) | 6.9 (4.7-9.1) | 9.5 (8.3-10.7) | 6.9 (5.1-8.7) | 8.9 (7.1-10.6) | 6.6 (4.3-9) | secernin 1 |
| 209278_s_at | TFPI2 | 1.613 | 0.011 |  |  | 4.4 (2.7-6.1) | 3.8 (3.2-4.3) | 4.3 (2.8-5.8) | 3.7 (3.3-4.1) | 4.3 (2.5-6.1) | 3.8 (3.3-4.3) | tissue factor pathway inhibitor 2 |
| 216733_s_at | GATM | 2.721 | 0.016 |  |  | 9.6 (7.3-11.9) | 7.7 (6-9.4) | 7.2 (4.8-9.6) | 8 (6.4-9.6) | 6.6 (4.5-8.8) | 9 (7-11) | glycine amidinotransferase (L-arginine:glycine amidinotransferase) |
| 206337_at | CCR7 | 2.859 | 0.016 |  |  | 6.8 (4.7-8.9) | 5.9 (3.9-7.8) | 5.7 (4.2-7.3) | 6.5 (4.6-8.5) | 5.9 (3.7-8.1) | 6.8 (4.4-9.1) | chemokine (C-C motif) receptor 7 |
| 229850_at | KDSR | 1.574 | 0.017 |  |  | 7.8 (6.7-9) | 6.2 (5.7-6.8) | 7.1 (5.5-8.7) | 6.4 (6-6.8) | 6.4 (5.3-7.4) | 6.5 (5.7-7.3) | 3-ketodihydrosphingosine reductase |
| 210538_s_at | BIRC3 | 1.988 | 0.017 |  |  | 10.4 (9.5-11.3) | 7.3 (5-9.6) | 9.5 (8.8-10.3) | 7.9 (5.8-10.1) | 8.1 (6.2-10) | 9.3 (6.8-11.8) | baculoviral IAP repeat containing 3 |
| 201909_at | RPS4Y1 | 3.627 | 0.019 |  |  | 8.7 (5.6-11.8) | 7.1 (4.9-9.2) | 8.5 (5.6-11.4) | 7 (5.1-8.8) | 7.9 (5-10.8) | 8.6 (5.9-11.3) | ribosomal protein S4, Y-linked 1 |
| 204409_s_at | EIF1AY | 3.570 | 0.019 | 0.002 |  | 6 (2.9-9.1) | 4.4 (2.3-6.5) | 5.8 (3-8.7) | 4.3 (2.4-6.1) | 5.3 (2.5-8.2) | 5.1 (2.7-7.6) | eukaryotic translation initiation factor 1A, Y-linked |
| 226063_at | VAV2 | 1.900 | 0.021 |  |  | 7.9 (6.6-9.1) | 6.2 (4.8-7.7) | 7.7 (6.9-8.6) | 6.9 (5.2-8.5) | 6.5 (5.1-8) | 6.6 (4.9-8.2) | vav 2 guanine nucleotide exchange factor |
| 211675_s_at | MDFIC | 3.133 | 0.022 |  |  | 10.5 (9.9-11.1) | 8.4 (6.5-10.3) | 9 (6.9-11.1) | 8.7 (7-10.5) | 8.9 (7-10.8) | 8.5 (5.8-11.2) | MyoD family inhibitor domain containing |
| 203232_s_at | ATXN1 | 1.527 | 0.023 |  |  | 8.5 (7.7-9.3) | 6.9 (5.4-8.3) | 7.5 (6.6-8.4) | 7.1 (5.5-8.7) | 7.3 (5.7-8.9) | 7.5 (5.8-9.2) | ataxin 1 |
| 228377_at | KLHL14 | 2.684 | 0.026 | 0.031 |  | 6.3 (3.8-8.8) | 4.5 (3.3-5.8) | 6.5 (4.3-8.8) | 4.4 (3.3-5.6) | 5.3 (2.8-7.8) | 4.2 (3.1-5.2) | kelch-like 14 (Drosophila) |
| 204430_s_at | SLC2A5 | 2.626 | 0.028 |  |  | 8.5 (6.6-10.3) | 5.7 (3.8-7.5) | 7.9 (5.6-10.3) | 7.2 (5-9.4) | 7.4 (4.9-10) | 6.8 (4.7-9) | solute carrier family 2 (facilitated glucose/fructose transporter), member 5 |
| 200872_at | S100A10 | 2.534 | 0.033 |  | 0.002 | 9.5 (7.5-11.4) | 8.2 (6.4-10) | 9.1 (7.4-10.8) | 9.1 (7.3-10.8) | 9.3 (7.4-11.2) | 7.3 (5.9-8.7) | S100 calcium binding protein A10 |
| 201565_s_at | ID2 | 2.001 | 0.038 |  |  | 10.9 (10.4-11.4) | 8.1 (6-10.2) | 8.7 (6.1-11.3) | 9.4 (8.1-10.8) | 9.5 (7.3-11.7) | 10.2 (9.5-10.9) | inhibitor of DNA binding 2, dominant negative helix-loop-helix protein |
| 217388_s_at | KYNU | 2.312 | 0.040 |  |  | 7.2 (5.1-9.3) | 4.6 (3.2-6.1) | 6.2 (3.9-8.5) | 6 (4.2-7.8) | 6.3 (4.3-8.3) | 6.1 (4.2-7.9) | kynureninase |
| 205780_at | BIK | 2.686 | 0.040 |  |  | 9.6 (7.9-11.2) | 7.6 (5.5-9.6) | 8.6 (7.1-10.1) | 8.8 (6.6-11) | 7.7 (6-9.5) | 9.6 (7.9-11.3) | BCL2-interacting killer (apoptosis-inducing) |
| 218723_s_at | RGCC | 3.029 | 0.041 |  |  | 8.4 (5.7-11.1) | 5.9 (4-7.7) | 8.4 (5.4-11.4) | 6.9 (5.3-8.6) | 6.9 (3.6-10.2) | 6.6 (4.5-8.7) | regulator of cell cycle |
| 220068_at | VPREB3 | 2.299 | 0.045 |  |  | 8.1 (6.1-10.2) | 7.1 (5.5-8.6) | 8.9 (6.4-11.3) | 7.3 (5.9-8.8) | 8.4 (5.8-11) | 7.8 (5.9-9.7) | pre-B lymphocyte 3 |
| 214669_x_at | IGKC | 2.969 | 0.056 |  |  | 11.8 (9.3-14.2) | 8.4 (6.8-10) | 11.1 (8.5-13.7) | 10.9 (8.7-13.1) | 10.8 (8-13.7) | 9.6 (7.3-11.8) | Immunoglobulin kappa constant |
| 205681_at | BCL2A1 | 2.767 | 0.057 |  |  | 7.7 (4.6-10.9) | 5.3 (3.8-6.7) | 6.9 (4.4-9.4) | 6.2 (4.2-8.3) | 6.1 (3.9-8.2) | 6.8 (4-9.5) | BCL2-related protein A1 |
| 208949_s_at | LGALS3 | 2.836 | 0.063 |  |  | 10.2 (8-12.3) | 7.2 (4.6-9.8) | 8.1 (6.2-10) | 8.6 (5.8-11.5) | 8.2 (5.5-10.9) | 7.8 (4.7-10.8) | lectin, galactoside-binding, soluble, 3 |
| 206126_at | CXCR5 | 2.329 | 0.069 | 0.035 |  | 7.4 (5-9.8) | 5.2 (4.8-5.7) | 7.3 (5.3-9.3) | 5.8 (4.4-7.2) | 6.1 (4.5-7.7) | 6.3 (4.6-8) | chemokine (C-X-C motif) receptor 5 |
| 203562_at | FEZ1 | 2.248 | 0.072 |  |  | 6.7 (4.9-8.6) | 4.4 (4.1-4.7) | 5.2 (4.4-6) | 5.8 (3.9-7.7) | 5.5 (4.3-6.7) | 5.9 (3.7-8) | fasciculation and elongation protein zeta 1 (zygin I) |
| 203685_at | BCL2 | 2.928 | 0.080 |  |  | 10.6 (9.7-11.6) | 8.7 (7-10.4) | 9.2 (6.7-11.6) | 8.7 (7.1-10.3) | 8.9 (6.7-11.2) | 9.2 (7.4-11) | B-cell CLL/lymphoma 2 |

Table S3. The gene list for the REGS predictors for each of the three drugs. The columns from left to right: 1) the Affymetrix GeneChip HG-U133 Plus 2.0 array probe-set id, 2) the gene symbol, 3) standard deviation for the 14 DLBCL cell lines, 4-6) weight in linear predictor for C, H, and O, 7-9) correlation between gene expression and measured resistance for C, H, and O, and 10) Gene title.

| Probeset | Gene Symbol | sd | C | H | O | Correlation C | Correlation H | Correlation O | Gene Title |
| --- | --- | --- | --- | --- | --- | --- | --- | --- | --- |
| disease |  |  | 57.29 | 44.33 | 32.79 |  |  |  |  |
| (Intercept) |  |  | 262.73 | 262.43 | 91.44 |  |  |  |  |
| 221933_at | NLGN4X | 2.31 | -5.26 | -0.06 |  | -0.72 (-0.87, -0.44) | -0.54 (-0.77, -0.2) | -0.45 (-0.72, -0.06) | neuroligin 4, X-linked |
| 202157_s_at | CELF2 | 2.13 | -4.60 |  |  | -0.25 (-0.59, 0.17) | 0.06 (-0.33, 0.44) | 0.28 (-0.14, 0.61) | CUGBP, Elav-like family member 2 |
| 224499_s_at | AICDA | 3.24 | -3.14 |  |  | -0.64 (-0.83, -0.32) | -0.28 (-0.6, 0.12) | -0.4 (-0.69, 0.01) | activation-induced cytidine deaminase |
| 242794_at | MAML3 | 2.13 | -2.99 | -0.84 |  | -0.58 (-0.8, -0.23) | -0.54 (-0.77, -0.2) | -0.23 (-0.58, 0.19) | mastermind-like 3 (Drosophila) |
| 226743_at | SLFN11 | 2.01 | -2.69 | -0.13 |  | -0.67 (-0.85, -0.37) | -0.62 (-0.81, -0.3) | -0.4 (-0.69, 0.01) | schlafen family member 11 |
| 231577_s_at | GBP1 | 2.05 | -2.08 | -1.76 |  | -0.59 (-0.8, -0.24) | -0.67 (-0.84, -0.38) | -0.31 (-0.64, 0.1) | guanylate binding protein 1, interferon-inducible |
| 212827_at | IGHM | 3.80 | -1.69 |  |  | -0.47 (-0.74, -0.09) | -0.31 (-0.62, 0.09) | -0.58 (-0.8, -0.23) | immunoglobulin heavy constant mu |
| 221675_s_at | CHPT1 | 2.38 | -1.66 |  |  | -0.28 (-0.61, 0.14) | 0.11 (-0.29, 0.48) | 0.09 (-0.33, 0.47) | choline phosphotransferase 1 |
| 217894_at | KCTD3 | 1.92 | -0.87 |  |  | -0.13 (-0.51, 0.29) | 0.24 (-0.16, 0.57) | 0.2 (-0.22, 0.56) | potassium channel tetramerisation domain containing 3 |
| 213241_at | PLXNC1 | 2.36 | -0.66 |  |  | -0.05 (-0.44, 0.36) | -0.12 (-0.48, 0.28) | 0.09 (-0.32, 0.48) | plexin C1 |
| 209470_s_at | GPM6A | 2.82 | -0.30 |  |  | -0.54 (-0.78, -0.18) | -0.39 (-0.67, 0) | -0.26 (-0.6, 0.16) | glycoprotein M6A |
| 210517_s_at | AKAP12 | 3.24 | -0.26 | -0.53 |  | -0.35 (-0.66, 0.06) | -0.25 (-0.58, 0.15) | -0.25 (-0.59, 0.17) | A kinase (PRKA) anchor protein 12 |
| 224588_at | XIST | 3.28 | -0.13 | -1.01 | -0.79 | -0.23 (-0.58, 0.2) | -0.3 (-0.61, 0.1) | -0.44 (-0.72, -0.04) | X (inactive)-specific transcript (non-protein coding) |
| 205173_x_at | CD58 | 3.18 |  | -2.63 |  | 0.21 (-0.21, 0.57) | -0.17 (-0.52, 0.24) | 0.08 (-0.33, 0.47) | CD58 molecule |
| 201300_s_at | PRNP | 1.95 |  | -2.44 |  | 0.03 (-0.38, 0.43) | -0.24 (-0.57, 0.16) | 0.18 (-0.24, 0.54) | prion protein |
| 203913_s_at | HPGD | 1.84 |  | -1.93 |  | -0.05 (-0.44, 0.36) | -0.38 (-0.67, 0) | -0.27 (-0.61, 0.15) | hydroxyprostaglandin dehydrogenase 15-(NAD) |
| 206632_s_at | APOBEC3B | 2.25 |  | -1.73 |  | 0.23 (-0.19, 0.58) | 0.07 (-0.32, 0.45) | 0.3 (-0.12, 0.63) | apolipoprotein B mRNA editing enzyme, catalytic polypeptide-like 3B |
| 201426_s_at | VIM | 3.02 |  | -1.36 |  | 0.14 (-0.28, 0.51) | -0.08 (-0.46, 0.31) | 0.16 (-0.26, 0.53) | vimentin |
| 218625_at | NRN1 | 2.39 |  | -1.34 | -0.01 | -0.42 (-0.7, -0.02) | -0.5 (-0.74, -0.14) | -0.47 (-0.73, -0.08) | neuritin 1 |
| 201599_at | OAT | 1.98 |  | -1.31 |  | 0.07 (-0.34, 0.46) | -0.11 (-0.48, 0.29) | 0.18 (-0.24, 0.55) | ornithine aminotransferase |
| 215001_s_at | GLUL | 3.36 |  | -1.25 |  | -0.41 (-0.7, 0) | -0.37 (-0.66, 0.02) | -0.05 (-0.44, 0.36) | glutamate-ammonia ligase |
| 205269_at | LCP2 | 1.58 |  | -1.13 |  | 0.11 (-0.31, 0.49) | -0.36 (-0.66, 0.03) | -0.08 (-0.47, 0.34) | lymphocyte cytosolic protein 2 (SH2 domain containing leukocyte protein of 76kDa) |
| 219049_at | CSGALNACT1 | 1.87 |  | -1.12 |  | -0.35 (-0.66, 0.06) | -0.52 (-0.76, -0.17) | 0.09 (-0.32, 0.48) | chondroitin sulfate N-acetylgalactosaminyltransferase 1 |
| 221690_s_at | NLRP2 | 2.57 |  | -1.08 |  | -0.05 (-0.45, 0.36) | -0.26 (-0.59, 0.14) | -0.38 (-0.68, 0.03) | NLR family, pyrin domain containing 2 |
| 206310_at | SPINK2 | 2.86 |  | -1.06 | -0.81 | -0.05 (-0.45, 0.36) | -0.28 (-0.6, 0.12) | -0.46 (-0.73, -0.07) | serine peptidase inhibitor, Kazal type 2 (acrosin-trypsin inhibitor) |
| 204836_at | GLDC | 2.21 |  | -0.98 |  | 0.12 (-0.3, 0.5) | -0.05 (-0.43, 0.35) | 0.28 (-0.14, 0.61) | glycine dehydrogenase (decarboxylating) |
| 204205_at | APOBEC3G | 1.77 |  | -0.93 |  | -0.2 (-0.56, 0.22) | -0.42 (-0.7, -0.04) | 0.06 (-0.35, 0.46) | apolipoprotein B mRNA editing enzyme, catalytic polypeptide-like 3G |
| 204646_at | DPYD | 2.50 |  | -0.90 |  | -0.09 (-0.48, 0.32) | -0.36 (-0.66, 0.03) | 0.26 (-0.17, 0.6) | dihydropyrimidine dehydrogenase |
| 210732_s_at | LGALS8 | 1.83 |  | -0.86 |  | -0.36 (-0.66, 0.05) | -0.67 (-0.84, -0.38) | -0.51 (-0.76, -0.14) | lectin, galactoside-binding, soluble, 8 |
| 204249_s_at | LMO2 | 2.36 |  | -0.85 |  | -0.25 (-0.6, 0.17) | -0.54 (-0.77, -0.2) | -0.63 (-0.82, -0.3) | LIM domain only 2 (rhombotin-like 1) |
| 202336_s_at | PAM | 2.33 |  | -0.77 |  | 0.14 (-0.28, 0.51) | 0 (-0.39, 0.38) | 0.02 (-0.39, 0.42) | peptidylglycine alpha-amidating monooxygenase |
| 229390_at | FAM26F | 2.50 |  | -0.63 |  | -0.22 (-0.57, 0.2) | -0.36 (-0.66, 0.03) | 0.1 (-0.31, 0.49) | family with sequence similarity 26, member F |
| 208892_s_at | DUSP6 | 2.61 |  | -0.38 |  | -0.18 (-0.54, 0.24) | -0.25 (-0.58, 0.16) | 0.15 (-0.27, 0.52) | dual specificity phosphatase 6 |
| 225626_at | PAG1 | 2.42 |  | -0.37 |  | -0.41 (-0.7, -0.01) | -0.61 (-0.81, -0.29) | -0.49 (-0.75, -0.11) | phosphoprotein associated with glycosphingolipid microdomains 1 |
| 205668_at | LY75 | 2.30 |  | -0.31 |  | -0.06 (-0.45, 0.35) | -0.29 (-0.61, 0.11) | -0.14 (-0.51, 0.28) | lymphocyte antigen 75 |
| 238462_at | UBASH3B | 2.32 |  | -0.22 |  | -0.21 (-0.57, 0.21) | -0.37 (-0.66, 0.02) | -0.17 (-0.54, 0.25) | ubiquitin associated and SH3 domain containing B |
| 227792_at | ITPRIPL2 | 1.88 |  | -0.18 |  | -0.13 (-0.5, 0.29) | -0.46 (-0.72, -0.08) | 0.2 (-0.22, 0.56) | inositol 1,4,5-trisphosphate receptor interacting protein-like 2 |
| 219563_at | LINC00341 | 1.64 |  | -0.13 |  | 0.04 (-0.37, 0.44) | -0.37 (-0.66, 0.02) | -0.24 (-0.59, 0.18) | long intergenic non-protein coding RNA 341 |
| 225673_at | MYADM | 2.23 |  | -0.10 |  | 0.17 (-0.25, 0.54) | -0.21 (-0.55, 0.19) | 0.11 (-0.3, 0.49) | myeloid-associated differentiation marker |
| 219304_s_at | PDGFD | 2.49 |  | -0.03 |  | -0.13 (-0.51, 0.29) | -0.26 (-0.59, 0.15) | -0.38 (-0.68, 0.02) | platelet derived growth factor D |
| 201739_at | SGK1 | 2.37 |  |  | -1.06 | 0.15 (-0.27, 0.52) | -0.04 (-0.42, 0.35) | -0.48 (-0.74, -0.1) | serum/glucocorticoid regulated kinase 1 |
| 201160_s_at | CSDA | 3.85 |  |  | -0.63 | 0.01 (-0.39, 0.41) | 0.26 (-0.15, 0.58) | -0.1 (-0.48, 0.32) | cold shock domain protein A |
| 205488_at | GZMA | 1.74 |  |  | -0.60 | -0.3 (-0.63, 0.12) | -0.42 (-0.7, -0.04) | -0.38 (-0.68, 0.03) | granzyme A (granzyme 1, cytotoxic T-lymphocyte-associated serine esterase 3) |
| 201242_s_at | ATP1B1 | 3.20 |  |  | -0.54 | -0.05 (-0.45, 0.36) | 0.05 (-0.34, 0.43) | -0.41 (-0.7, 0) | ATPase, Na+/K+ transporting, beta 1 polypeptide |
| 211597_s_at | HOPX | 3.38 |  |  | -0.45 | -0.18 (-0.55, 0.24) | -0.37 (-0.66, 0.02) | -0.54 (-0.77, -0.17) | HOP homeobox |
| 216733_s_at | GATM | 2.72 |  |  | -0.28 | 0.13 (-0.29, 0.5) | -0.04 (-0.42, 0.35) | -0.3 (-0.63, 0.11) | glycine amidinotransferase (L-arginine:glycine amidinotransferase) |
| 220330_s_at | SAMSN1 | 2.50 |  |  | -0.11 | -0.09 (-0.47, 0.33) | -0.31 (-0.63, 0.08) | -0.65 (-0.83, -0.34) | SAM domain, SH3 domain and nuclear localization signals 1 |
| 1554733_at | LOC728175 | 2.30 |  |  | -0.10 | -0.26 (-0.6, 0.16) | -0.37 (-0.66, 0.02) | -0.43 (-0.71, -0.03) | uncharacterized LOC728175 |
| 209480_at | HLA-DQB1 | 2.68 |  |  | -0.05 | -0.04 (-0.44, 0.37) | -0.04 (-0.42, 0.35) | -0.39 (-0.69, 0.02) | major histocompatibility complex, class II, DQ beta 1 |
| 227856_at | C4orf32 | 2.39 |  |  | 0.00 | -0.21 (-0.57, 0.21) | -0.15 (-0.51, 0.25) | -0.35 (-0.66, 0.06) | chromosome 4 open reading frame 32 |
| 225763_at | RCSD1 | 2.78 |  |  | 0.03 | 0.06 (-0.36, 0.45) | 0.01 (-0.38, 0.4) | 0.39 (-0.02, 0.68) | RCSD domain containing 1 |
| 217979_at | TSPAN13 | 2.70 |  |  | 0.05 | -0.03 (-0.42, 0.38) | -0.21 (-0.55, 0.19) | 0.35 (-0.07, 0.66) | tetraspanin 13 |
| 215016_x_at | DST | 2.09 |  |  | 0.11 | 0.53 (0.17, 0.77) | 0.45 (0.08, 0.71) | 0.61 (0.28, 0.81) | dystonin /// dystonin-like |
| 216379_x_at | CD24 | 3.19 |  |  | 0.15 | -0.52 (-0.76, -0.14) | -0.41 (-0.69, -0.03) | -0.25 (-0.59, 0.17) | CD24 molecule |
| 1555989_at |  | 1.90 |  |  | 0.55 | 0.03 (-0.38, 0.43) | 0.05 (-0.35, 0.43) | 0.39 (-0.01, 0.69) |  |
| 219335_at | ARMCX5 | 1.87 |  |  | 0.57 | 0.07 (-0.35, 0.46) | 0.23 (-0.17, 0.57) | 0.62 (0.3, 0.82) | armadillo repeat containing, X-linked 5 |
| 221234_s_at | BACH2 | 2.36 |  |  | 0.77 | -0.36 (-0.67, 0.05) | -0.17 (-0.53, 0.23) | 0.14 (-0.28, 0.51) | BTB and CNC homology 1, basic leucine zipper transcription factor 2 |
| 220595_at | PDZRN4 | 2.23 |  | 0.02 |  | 0.08 (-0.33, 0.47) | 0.21 (-0.19, 0.56) | -0.1 (-0.49, 0.31) | PDZ domain containing ring finger 4 |
| 204992_s_at | PFN2 | 2.33 |  | 0.04 | 0.78 | 0.22 (-0.2, 0.57) | 0.49 (0.13, 0.74) | 0.52 (0.15, 0.76) | profilin 2 |
| 225391_at | LOC93622 | 1.73 |  | 0.07 |  | 0.37 (-0.04, 0.67) | 0.32 (-0.07, 0.63) | 0.39 (-0.01, 0.69) | Morf4 family associated protein 1-like 1 pseudogene |
| 205000_at | DDX3Y | 3.15 |  | 0.10 |  | 0.04 (-0.37, 0.44) | 0.04 (-0.35, 0.42) | -0.17 (-0.54, 0.25) | DEAD (Asp-Glu-Ala-Asp) box polypeptide 3, Y-linked |
| 204913_s_at | SOX11 | 2.78 |  | 0.13 |  | -0.07 (-0.46, 0.34) | 0.18 (-0.22, 0.53) | -0.28 (-0.61, 0.14) | SRY (sex determining region Y)-box 11 |
| 219737_s_at | PCDH9 | 2.60 |  | 0.28 |  | -0.24 (-0.58, 0.18) | -0.14 (-0.5, 0.26) | -0.06 (-0.45, 0.35) | protocadherin 9 |
| 225129_at | CPNE2 | 2.07 |  | 0.29 |  | -0.03 (-0.43, 0.38) | 0.19 (-0.21, 0.54) | -0.04 (-0.44, 0.37) | copine II |
| 203680_at | PRKAR2B | 2.99 |  | 0.36 |  | -0.13 (-0.51, 0.29) | 0.08 (-0.31, 0.46) | -0.18 (-0.55, 0.24) | protein kinase, cAMP-dependent, regulatory, type II, beta |
| 231579_s_at | TIMP2 | 1.85 |  | 0.42 |  | 0.36 (-0.05, 0.66) | 0.58 (0.25, 0.79) | 0.5 (0.12, 0.75) | TIMP metallopeptidase inhibitor 2 |
| 225532_at | CABLES1 | 2.07 |  | 0.46 |  | 0.28 (-0.14, 0.62) | 0.49 (0.13, 0.74) | -0.01 (-0.41, 0.4) | Cdk5 and Abl enzyme substrate 1 |
| 207039_at | CDKN2A | 1.99 |  | 0.78 |  | 0.27 (-0.15, 0.61) | 0.5 (0.14, 0.74) | 0.12 (-0.29, 0.5) | cyclin-dependent kinase inhibitor 2A |
| 213436_at | CNR1 | 3.06 |  | 0.80 |  | -0.07 (-0.46, 0.34) | -0.11 (-0.48, 0.29) | -0.25 (-0.59, 0.18) | cannabinoid receptor 1 (brain) |
| 229656_s_at | EML6 | 2.42 |  | 0.81 |  | 0.08 (-0.33, 0.47) | 0.26 (-0.15, 0.58) | -0.04 (-0.43, 0.37) | echinoderm microtubule associated protein like 6 |
| 1552531_a_at | NLRP11 | 2.46 |  | 0.86 |  | 0.29 (-0.12, 0.62) | 0.2 (-0.2, 0.55) | -0.21 (-0.56, 0.21) | NLR family, pyrin domain containing 11 |
| 225386_s_at | HNRPLL | 2.48 |  | 1.19 |  | 0.46 (0.07, 0.73) | 0.73 (0.47, 0.87) | 0.32 (-0.09, 0.64) | heterogeneous nuclear ribonucleoprotein L-like |
| 226190_at | MAP3K13 | 2.68 |  | 1.40 |  | 0.42 (0.02, 0.7) | 0.57 (0.23, 0.78) | 0.45 (0.06, 0.73) | mitogen-activated protein kinase kinase kinase 13 |
| 209524_at | HDGFRP3 | 2.80 |  | 1.44 |  | -0.19 (-0.55, 0.24) | 0.03 (-0.37, 0.41) | -0.08 (-0.47, 0.34) | hepatoma-derived growth factor, related protein 3 |
| 228297_at |  | 2.22 |  | 1.81 |  | 0.18 (-0.24, 0.54) | 0.32 (-0.08, 0.63) | -0.12 (-0.5, 0.3) |  |
| 201909_at | RPS4Y1 | 3.63 | 0.22 |  |  | 0.06 (-0.36, 0.45) | 0.02 (-0.37, 0.4) | -0.14 (-0.51, 0.28) | ribosomal protein S4, Y-linked 1 |
| 205681_at | BCL2A1 | 2.77 | 0.27 |  |  | -0.13 (-0.51, 0.29) | -0.32 (-0.63, 0.07) | -0.57 (-0.79, -0.22) | BCL2-related protein A1 |
| 1568752_s_at | RGS13 | 3.30 | 0.34 |  |  | -0.17 (-0.54, 0.25) | -0.26 (-0.59, 0.14) | -0.43 (-0.71, -0.03) | regulator of G-protein signaling 13 |
| 204730_at | RIMS3 | 1.67 | 0.49 |  |  | 0.06 (-0.35, 0.46) | 0.04 (-0.35, 0.42) | -0.2 (-0.56, 0.22) | regulating synaptic membrane exocytosis 3 |
| 206126_at | CXCR5 | 2.33 | 0.53 |  |  | -0.05 (-0.44, 0.36) | -0.11 (-0.48, 0.29) | -0.48 (-0.74, -0.1) | chemokine (C-X-C motif) receptor 5 |
| 206337_at | CCR7 | 2.86 | 0.87 |  |  | -0.23 (-0.58, 0.19) | -0.4 (-0.68, -0.02) | -0.54 (-0.77, -0.17) | chemokine (C-C motif) receptor 7 |
| 204141_at | TUBB2A | 1.99 | 0.95 |  |  | 0.18 (-0.24, 0.54) | -0.11 (-0.47, 0.29) | -0.18 (-0.55, 0.24) | tubulin, beta 2A class IIa |
| 201565_s_at | ID2 | 2.00 | 0.98 |  |  | 0.24 (-0.18, 0.58) | -0.13 (-0.49, 0.28) | -0.23 (-0.58, 0.19) | inhibitor of DNA binding 2, dominant negative helix-loop-helix protein |
| 203685_at | BCL2 | 2.93 | 1.28 |  |  | 0.37 (-0.04, 0.67) | 0.12 (-0.28, 0.49) | 0.07 (-0.34, 0.46) | B-cell CLL/lymphoma 2 |
| 217967_s_at | FAM129A | 2.65 | 1.64 |  |  | 0.37 (-0.04, 0.67) | 0.02 (-0.37, 0.4) | 0.26 (-0.16, 0.6) | family with sequence similarity 129, member A |
| 200872_at | S100A10 | 2.53 | 1.74 |  |  | 0.23 (-0.19, 0.58) | 0.12 (-0.28, 0.48) | 0.43 (0.03, 0.71) | S100 calcium binding protein A10 |
| 214669_x_at | IGKC | 2.97 | 2.24 | 1.10 |  | 0.26 (-0.16, 0.6) | 0.1 (-0.3, 0.47) | 0.02 (-0.39, 0.42) | Immunoglobulin kappa constant |
| 1556096_s_at | UNC13C | 3.04 | 2.96 |  |  | 0.53 (0.16, 0.77) | 0.25 (-0.15, 0.58) | 0.01 (-0.4, 0.41) | unc-13 homolog C (C. elegans) |
| 218723_s_at | RGCC | 3.03 | 3.66 |  |  | 0.02 (-0.39, 0.42) | -0.18 (-0.53, 0.22) | -0.28 (-0.62, 0.14) | regulator of cell cycle |

Table S4: Cox proportional hazards analyses **of the association between PFS and OS and the classification of the clinical cohorts for CHO and the three individual drugs.** In the multivariate analysis the Cox proportional hazards regression is adjusted for IPI. The estimated HR’s compare patients classified as resistant to patients classified as sensitive.

|  | **Univariate** | | |  | | **Multivariate** | | |
| --- | --- | --- | --- | --- | --- | --- | --- | --- |
|  | N | HR (95% CI) | P-value |  | N | | HR (95% CI) | P-value |
| **CHO** |  |  |  |  |  | |  |  |
| IDRC (PFS) | 470 | 2.42 (1.63,3.59) | 1.24e-05 |  | 424 | | 2.24 (1.46,3.44) | 0.0002 |
| LLMPP (PFS) | 220 | 2.01 (0.96,4.19) | 0.0633 |  | 180 | | 2.43 (1.05,5.59) | 0.0374 |
| MDFCI (OS) | 67 | 14.04 (1.82,108.09) | 0.0112­ |  | 63 | | 13.90 (1.80,107.43) | 0.0117 |
| **Cyclophosphamide (C)** | | | | | | | |  |
| IDRC (PFS) | 470 | 1.03 (0.72,1.47) | 0.864 |  | 424 | | 0.98 (0.68,1.42) | 0.92 |
| LLMPP (PFS) | 220 | 0.77 (0.41,1.43) | 0.403 |  | 180 | | 1.06 (0.53,2.12) | 0.859 |
| MDFCI (OS) | 67 | 0.66 (0.23,1.89) | 0.441 |  | 63 | | 0.85 (0.28,2.53) | 0.767 |
| **Doxorubicin (H)** | | | | | | | | |
| IDRC (PFS) | 470 | 2.58 (1.72,3.86) | 4.37e-06 |  | 424 | | 2.52 (1.64,3.87) | 2.65e-05 |
| LLMPP (PFS) | 220 | 2.28 (1.10,4.73) | 0.0269 |  | 180 | | 2.52 (1.13,5.64) | 0.0237 |
| MDFCI (OS) | 67 | 4.56 (1.29,16.19) | 0.0188 |  | 63 | | 4.05 (1.13,14.51) | 0.0318 |
| **Vincristine (O)** | | | | | | | | |
| IDRC (PFS) | 470 | 1.80 (1.23,2.64) | 0.00241 |  | 424 | | 1.50 (1.01,2.23) | 0.0454 |
| LLMPP (PFS) | 220 | 3.05 (1.51,6.15) | 0.00184 |  | 180 | | 2.56 (1.22,5.39) | 0.0134 |
| MDFCI (OS) | 67 | 5.67 (1.24,25.96) | 0.0253 |  | 63 | | 5.05 (1.08,23.47) | 0.039 |

Table S5. Cox proportional hazards analyses **of the association between PFS and OS and the predicted resistance indices for CHO and the three individual drugs.** In the multivariate analyses the Cox proportional hazards regressions are adjusted for IPI. The estimated HR’s are based on an increase of 10 in the *AUC_0_*.

|  | **Univariate** | | |  | | **Multivariate** | | |
| --- | --- | --- | --- | --- | --- | --- | --- | --- |
|  | N | HR (95% CI) | P-value |  | N | | HR (95% CI) | P-value |
| **CHO** |  |  |  |  |  | |  |  |
| IDRC (PFS) | 470 | 1.24 (1.09,1.42) | 0.00153 |  | 424 | | 1.49 (1.19,1.87) | 0.00055 |
| LLMPP (PFS) | 220 | 1.18 (0.98,1.44) | 0.0876 |  | 220 | | 1.18 (0.98,1.44) | 0.0876 |
| MDFCI (OS) | 67 | 1.91 (1.28,2.87) | 0.00166 |  | 63 | | 2.49 (1.22,5.05) | 0.0117 |
| **Cyclophosphamide (C)** | | | | | | | | |
| IDRC (PFS) | 470 | 0.98 (0.92,1.03) | 0.371 |  | 424 | | 0.98 (0.93,1.04) | 0.598 |
| LLMPP (PFS) | 220 | 0.95 (0.87,1.05) | 0.315 |  | 180 | | 0.96 (0.87,1.07) | 0.511 |
| MDFCI (OS) | 67 | 1.06 (0.86,1.31) | 0.598 |  | 63 | | 1.14 (0.90,1.43) | 0.284 |
| **Doxorubicin (H)** | | | | | | | | |
| IDRC (PFS) | 470 | 1.11 (1.04,1.17) | 0.000572 |  | 424 | | 1.09 (1.03,1.16) | 0.00595 |
| LLMPP (PFS) | 220 | 1.10 (1.00,1.20) | 0.0438 |  | 180 | | 1.11 (1.00,1.23) | 0.0433 |
| MDFCI (OS) | 67 | 1.32 (1.14,1.54) | 0.000318 |  | 63 | | 1.34 (1.15,1.57) | 0.00023 |
| **Vincristine (O)** | | | | | | | | |
| IDRC (PFS) | 470 | 1.56 (1.26,1.94) | 5.48E-05 |  | 424 | | 1.49 (1.19,1.87) | 0.00055 |
| LLMPP (PFS) | 220 | 1.99 (1.36,2.92) | 0.000399 |  | 180 | | 1.83 (1.22,2.74) | 0.00341 |
| MDFCI (OS) | 67 | 2.73 (1.29,5.80) | 0.00872 |  | 63 | | 2.49 (1.22,5.05) | 0.0117 |
